# Design and synthesis of novel quinazolinone-based derivatives as EGFR inhibitors with antitumor activity

**DOI:** 10.1080/14756366.2022.2118735

**Published:** 2022-09-22

**Authors:** Amr Sonousi, Rasha A. Hassan, Eman O. Osman, Amr M. Abdou, Soha H. Emam

**Affiliations:** a Pharmaceutical Organic Chemistry Department, Faculty of Pharmacy, Cairo University, Cairo, Egypt; b Department of Microbiology and Immunology, National Research Centre, Dokki, Giza, Egypt

**Keywords:** Quinazolin-4(3*H*)-one, antiproliferative activity, molecular modelling, EGFR inhibition, cell cycle analysis

## Abstract

Nineteen new quinazolin-4(3*H*)-one derivatives **3a–g** and **6a–l** were designed and synthesised to inhibit EGFR. The antiproliferative activity of the synthesised compounds was tested *in vitro* against 60 different human cell lines. The most potent compound **6d** displayed superior sub-micromolar antiproliferative activity towards NSC lung cancer cell line NCI-H460 with GI_50_ = 0.789 µM. It also showed potent cytostatic activity against 40 different cancer cell lines (TGI range: 2.59–9.55 µM). Compound **6d** potently inhibited EGFR with IC_50_ = 0.069 ± 0.004 µM in comparison to erlotinib with IC_50_ value of 0.045 ± 0.003 µM. Compound **6d** showed 16.74-fold increase in total apoptosis and caused cell cycle arrest at G1/S phase in breast cancer HS 578T cell line. Moreover, the most potent derivatives were docked into the EGFR active site to determine their binding mode and confirm their ability to satisfy the pharmacophoric features required for EGFR inhibition.

## Introduction

Cancer is a widespread and lethal noncommunicable disease with rapidly growing incidence and mortality worldwide^1^. Breast cancer is the leading cause of death among women with a reported incidence of approximately 2.3 million and 685,000 deaths worldwide in 2020^2^.

**Figure 1.**
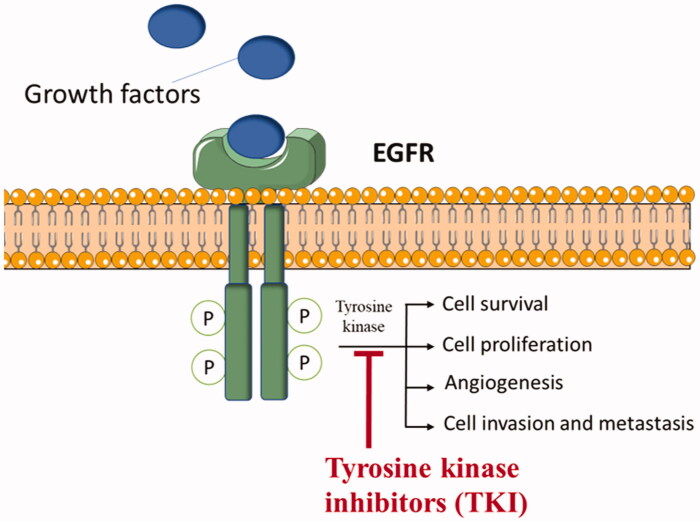
An illustrative diagram showing the mechanism of action of TKIs as anticancer.

Molecular targeting therapy approach, that targets a crucial cancer-related enzyme or receptor, increases the tumour specificity and decreases the side effects^3–5^. The epidermal growth factor receptor (EGFR) is mainly involved in the growth factor signalling. Abnormal signalling and overexpression of this receptor enhance downstream effects such as cell survival, cell proliferation and angiogenesis which lead to uncontrolled cell proliferation and metastasis, which ultimately promote tumour growth (Figure 1)^6^. The EGFR is one of the members of ErbB tyrosine kinase receptors family^7^, and consists of two domains; an extracellular receptor domain connected via a transmembrane region to an intracellular domain with tyrosine kinase function^8^. Inhibition of EGFR by tyrosine kinase inhibitors (TKIs) delays these downstream effects and lead to inhibition of tumour growth. Many breast cancers express 2 × 10^6^ EGFR molecules per cell which is more than 20-fold the expression of EGFR in normal cells^9^^,^^10^. This overexpression of EGFR in breast cancer as well as other cancer cells including colon and lung cancers made this a potential molecular target for inhibition^11–14^.

Three generations of small-molecule EGFR TKIs have been developed so far^15^^,^^16^. The first-generation, such as gefitinib and erlotinib usually with 4-anilinoquinazoline motifs, achieves initial good responses in the treatment of cancer patients with overexpression of EGFR but unfortunately, resistance to this class was acquired by most patients within 1 year^17^. The second-generation of EGFR TKIs, such as neratinib and afatinib, were developed to overcome the acquired resistance^18^^,^^19^, however their associated side effects^20^^,^^21^ lead to the design of a novel class of third-generation EGFR TKIs, including osimertinib, rociletinib and olmutinib. This class has been designed and developed to overcome resistance and reduce side effects^22^. The aim of this study is to design, synthesise and evaluate compounds with the same pharmacophoric features as the third generation EGFR TKIs.

**Figure 2.**
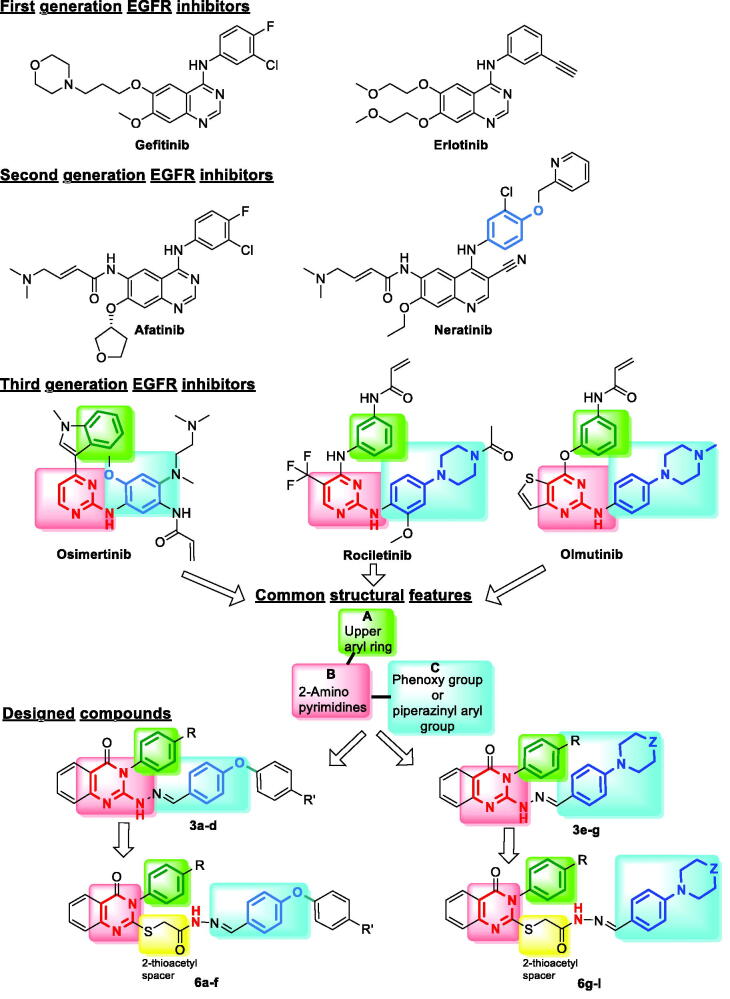
The three generations of EGFR inhibitors and the designed target compounds.

In previous studies in our lab, we synthesised different TKIs with high therapeutic index^23–27^. In the present study, the design of the new compounds started by finding common pharmacophoric structural features of the newer generations of EGFR TKI without including the acrylamide moiety. The acrylamide moiety in the second and the third generations gives strong covalent binding to the SH group in Cys797 residue of the receptor. However, these compounds are prone to drug resistance when this irreversible covalent interaction is lost due to C797S mutation^28^^,^^29^. The common structural features were found to be (A) an upper aryl ring attached to (B) 2-aminopyrimidines or 2-amino arylfusedpyrimidines and the 2-amino is attached to either C) phenoxy group or piperazinyl aryl group (Figure 2). The design of the novel compounds was done to mimic these structural features of the third generation EGFR TKIs to have 2-aminoarylquinazoline linked to an upper aryl ring and the 2-amino is attached to either substituted phenoxy group (**3a–d**) or piperazinyl aryl (or its isostere) (**3e–g**). We also designed congener compounds with a 2-thioacetyl spacer between the pyrimidine ring and either phenoxy group **(6a–f)** or piperazinyl aryl **(6g–l)** groups to investigate the effect of this spacer on the activity of the compounds (Figure 2). The designed compounds were synthesised and subjected to *in vitro* screening of their cytotoxic activity against a panel of 60 different cancer cell lines. The compounds were investigated for their ability to inhibit EGFR, their effect on cancer cell cycle and their ability to induce apoptosis. Molecular modelling studies were done as well to rationalise the biological activity of the synthesised compounds.

## Experimental

### Chemistry

#### General

Melting points were obtained on a Griffin apparatus and were uncorrected. Microanalyses for C, H and N were carried out at the Regional Centre for Mycology and Biotechnology, Faculty of Pharmacy, Al-Azhar University. IR spectra were recorded on Shimadzu IR 435 spectrophotometer (Shimadzu Corp., Kyoto, Japan) Faculty of Pharmacy, Cairo University, Cairo, Egypt, and values were represented in cm^−1^. ^1^H NMR spectra were carried out on Bruker 400 MHz (Bruker Corp., Billerica, MA, USA) spectrophotometer, Faculty of Pharmacy, Cairo University, Cairo, Egypt. The chemical shifts were recorded in ppm on *δ* scale, coupling constants (*J*) were given in Hz and peak multiplicities are designed as follows: s, singlet; d, doublet; dd, doublet of doublets; t, triplet; m, multiplet ^13 ^C NMR spectra were carried out on Bruker 100 MHz spectrophotometer, Faculty of Pharmacy, Cairo University, Cairo, Egypt. Mass spectra were recorded with Advion expression^®^ CMS, Nawah Scientific, Cairo, Egypt. Progress of the reactions were monitored by TLC using precoated aluminium sheet silica gel MERCK 60 F 254 and was visualised by UV lamp. The original NMR spectra of the investigated compounds are provided as supporting information. Compounds **1a,b**^30^^,^^31^, **2a,b**^31^, **4a,b**^32^^,^^33^ and **5a,b**^32^^,^^33^ were prepared as reported.

##### General procedure for the preparation of 2–(2-(4–(4-substituted) benzylidene) hydrazinyl)-3-phenylquinazolin-4(*3H*)-one (3a-g)

To a mixture of 2-hydrazino-quinazolinone **2a,b** (5 mmol) and the appropriate aryloxybenzaldehydes or dialkylaminobenzaldehydes (5 mmol) in absolute ethanol (50 mL), few drops of acetic acid were added, and the reaction mixture was refluxed for 6 h then cooled. The precipitate, formed upon cooling, was collected by filtration, washed with water crystallised from ethanol to give the corresponding hydrazone derivative **3a-g.**

###### 2–(2-(4–(4-chlorophenoxy)benzylidene)hydrazinyl)-3-phenylquinazolin-4(3H)-one (3a)

Yellow solid: 89% yield; mp 158–164 °C; IR (KBr, cm^−1^) 3444 (NH), 3078 (CH aromatic), 2924, 2854 (CH aliphatic), 1651 (C=O), 1593 (C=N); ^1^H NMR (400 MHz, DMSO-d_6_) δ 10.58 (NH, D_2_O exchangeable), 8.06 (s, 1H, CH=N), 7.96 (d, *J*=8.8 Hz, 2H, ArH), 7.91 (d, *J*=7.2 Hz, 1H, ArH), 7.69–7.66 (m, 2H, ArH), 7.52–7.41 (m, 5H, ArH), 7.35–7.32 (m, 2H, ArH), 7.19–7.15 (m, 1H, ArH), 7.10–7.05 (m, 4H, ArH); ^13 ^C NMR (100 MHz, DMSO-d_6_) δ 161.1, 158.0, 155.6, 152.8, 151.8, 140.4, 136.9, 135.5, 131.4, 130.4, 130.2, 129.7, 129.3, 128.4, 128.0, 127.8, 122.3, 121.0, 118.9, 116.1, 114.8; Anal. Calcd for C_27_H_19_ClN_4_O_2_: C, 69.45; H, 4.10; N, 12.00; found C, 69.19; H, 4.28; N, 12.26; MS (ESI) *m/z*: 465 [M − H]^−^.

###### 2–(2-(4–(4-bromophenoxy)benzylidene)hydrazinyl)-3-phenylquinazolin-4(3H)-one (3b)

White solid: 92% yield; mp 198–200 °C; IR (KBr, cm^−1^) 3332 (NH), 3051 (CH aromatic), 2974, 2943 (CH aliphatic), 1685 (C=O), 1616 (C=N); ^1^H NMR (400 MHz, DMSO-d_6_) δ 10.59 (NH, D_2_O exchangeable), 8.06 (s, 1H, CH=N), 7.96 (d, *J*=8.4 Hz, 2H, ArH), 7.91 (d, *J*=7.6 Hz, 1H, ArH), 7.71–7.66 (m, 2H, ArH), 7.57 (d, *J*=8.4 Hz, 2H, ArH), 7.50 (t, *J*=7.6 Hz, 2H, ArH), 7.41–7.40 (m, 1H, ArH), 7.34 (d, *J*=7.6 Hz, 2H, ArH), 7.18–7.14 (m, 1H, ArH), 7.07 (d, *J*=8.0 Hz, 2H, ArH), 7.03 (d, *J*=8.0 Hz, 2H, ArH); ^13 ^C NMR (100 MHz, DMSO-d_6_) δ 161.1, 157.8, 156.1, 152.8, 151.8, 140.3, 136.9, 135.6, 133.4, 131.4, 130.2, 129.7, 129.3, 128.4, 127.8, 122.3, 121.4, 119.0, 116.1, 115.9, 114.8.; Anal. Calcd for C_27_H_19_BrN_4_O_2_: C, 63.42; H, 3.75; N, 10.96, found C, 63.19; H, 3.87; N, 11.20.

###### 2–(2-(4–(4-methoxyphenoxy)benzylidene)hydrazinyl)-3-phenylquinazolin-4(3H)-one (3c)

Yellow solid: 80% yield; mp 185–188 °C; IR (KBr, cm^−1^) 3332 (NH), 3094 (CH aromatic), 2962, 2846 (CH aliphatic), 1681 (C=O), 1600 (C=N); ^1^H NMR (400 MHz, DMSO-d_6_) δ 10.57 (NH, D_2_O exchangeable), 8.03 (s, 1H, CH=N), 7.92–7.89 (m, 3H, ArH), 7.69–7.66 (m, 2H, ArH), 7.51–7.47 (m, 2H, ArH), 7.44–7.39 (m, 1H, ArH), 7.35–7.32 (m, 2H, ArH), 7.18–7.14 (m, 1H, ArH), 7.06–7.03 (m, 2H, ArH), 7.01–6.97 (m, 2H, ArH), 6.97–6.93 (m, 2H, ArH), 3.76 (s, 3H, OCH_3_); ^13 ^C NMR (100 MHz, DMSO-d_6_) δ 161.1, 159.8, 156.4, 153.0, 151.6, 149.2, 140.4, 136.9, 135.5, 130.2, 130.0, 129.7, 129.3, 128.4, 127.8, 122.2, 121.5, 117.3, 116.1, 115.6, 114.8, 55.9; Anal. Calcd for C_28_H_22_N_4_O_3_: C, 72.71; H, 4.79; N, 12.11; found C, 72.53; H, 4.91; N, 12.38; MS (ESI) *m/z*: 463 [M + H^+^].

###### 2–(2-(4–(4-chlorophenoxy)benzylidene)hydrazinyl)-3–(4-chlorophenyl)quinazolin-4(3H)-one (3d)

White solid: 85% yield; mp 212–214 °C; IR (KBr, cm^−1^) 3340 (NH), 3059 (CH aromatic), 2958, 2924 (CH aliphatic), 1693 (C=O), 1612 (C=N); ^1^H NMR (400 MHz, DMSO-d_6_) δ 10.61 (NH, D_2_O exchangeable), 8.09 (s, 1H, CH=N), 7.96 (d, *J*=8.4 Hz, 2H, ArH), 7.91 (d, *J*=7.6 Hz, 1H, ArH), 7.70–7.66 (m, 2H, ArH), 7.57–7.54 (m, 2H, ArH), 7.48–7.44 (m, 2H, ArH), 7.42–7.39 (m, 2H, ArH), 7.19–7.15 (m, 1H, ArH), 7.10–7.06 (m, 4H, ArH); ^13 ^C NMR (100 MHz, DMSO-d_6_) δ 161.0, 158.0, 155.6, 153.0, 151.7, 140.4, 135.8, 135.6, 133.0, 131.7, 131.4, 130.4, 130.2, 129.4, 128.0, 127.8, 122.3, 121.0, 118.9, 116.1, 114.7; Anal. Calcd for C_27_H_18_Cl_2_N_4_O_2_: C, 64.68; H, 3.62; N, 11.17, found C, 64.90; H, 3.85; N, 11.39.

###### 3-phenyl-2–(2-(4-(piperidin-1-yl)benzylidene)hydrazinyl)quinazolin-4(3H)-one (3e)

Pale yellow solid: 82% yield; mp 160–166 °C; IR (KBr, cm^−1^) 3332 (NH), 3051 (CH aromatic), 2924, 2846 (CH aliphatic), 1681 (C=O), 1600 (C=N); ^1^H NMR (400 MHz, DMSO-d_6_) δ 10.49 (NH, D_2_O exchangeable), 7.93 (s, 1H, CH=N), 7.90 (d, *J*=7.6 Hz, 1H, ArH), 7.73 (d, *J*=8.4 Hz, 2H, ArH), 7.67 (d, *J*=3.6 Hz, 2H, ArH), 7.51–7.47 (m, 2H, ArH), 7.43–7.40 (m, 1H, ArH), 7.34–7.32 (m, 2H, ArH), 7.16–7.12 (m, 1H, ArH), 6.92 (d, *J*=8.8 Hz, 2H, ArH), 3.24 (t, *J*=4.4 Hz, 4H, 2CH_2_ piperidine), 1.62–1.53 (m, 6H, 3CH_2_ piperidine); ^13 ^C NMR (100 MHz, DMSO-d_6_) δ 161.1, 154.0, 152.7, 150.8, 140.5, 137.0, 135.5, 129.7, 129.6, 129.3, 128.3, 127.8, 125.1, 122.0, 116.0, 114.9, 114.7, 49.0, 25.5, 24.4; Anal. Calcd for C_26_H_25_N_5_O: C, 73.74; H, 5.95; N, 16.54, found C, 73.79; H, 5.80; N, 16.41; MS (ESI) *m/z*: 446 [M + Na^+^].

###### 2–(2-(4-morpholinobenzylidene)hydrazinyl)-3-phenylquinazolin-4(3H)-one (3f)

White solid: 79% yield; mp 242–244 °C; IR (KBr, cm^−1^) 3325 (NH), 3092 (CH aromatic), 2939, 2894 (CH aliphatic), 1680 (C=O), 1604 (C=N); ^1^H NMR (400 MHz, DMSO-d_6_) δ 10.50 (NH, D_2_O exchangeable), 7.94 (s, 1H, CH=N), 7.90 (d, *J*=7.6 Hz, 1H, ArH), 7.77 (d, *J*=8.4 Hz, 2H, ArH), 7.67 (d, *J*=4.0 Hz, 2H, ArH), 7.51–7.47 (m, 2H, ArH), 7.43–7.39 (m, 1H, ArH), 7.34–7.31 (m, 2H, ArH), 7.16–7.12 (m, 1H, ArH), 6.96 (d, *J*=8.8 Hz, 2H, ArH), 3.74 (t, *J*=4.6 Hz, 4H, 2CH_2_ morpholine), 3.19 (t, *J*=4.6 Hz, 4H, 2CH_2_ morpholine); ^13 ^C NMR (100 MHz, DMSO-d_6_) δ 161.2, 153.9, 152.4, 151.0, 140.4, 136.8, 135.6, 129.6, 129.5, 129.4, 128.4, 127.8, 126.0, 122.1, 116.0, 114.6, 66.4, 48.0; Anal. Calcd for C_25_H_23_N_5_O_2_: C, 70.57; H, 5.45; N, 16.46; found C, 70.78; H, 5.71; N, 16.70; MS (ESI) *m/z*: 448 [M + Na^+^].

###### 2–(2-(4–(4-methylpiperazin-1-yl)benzylidene)hydrazinyl)-3-phenylquinazolin-4(3H)-one (3g)

Yellow solid: 85% yield; mp 208–210 °C; IR (KBr, cm^−1^) 3325 (NH), 3093 (CH aromatic), 2974, 2890 (CH aliphatic), 1678 (C=O), 1612 (C=N); ^1^H NMR (400 MHz, DMSO-d_6_) δ 10.50 (NH, D_2_O exchangeable), 7.93 (s, 1H, CH=N), 7.89 (d, *J*=8.0 Hz, 1H, ArH), 7.75 (d, *J*=8.0 Hz, 2H, ArH), 7.66 (d, *J*=4.0 Hz, 2H, ArH), 7.51–7.47 (m, 2H, ArH), 7.43–7.39 (m, 1H, ArH), 7.34–7.31 (m, 2H, ArH), 7.16–7.12 (m, 1H, ArH), 6.96–6.93 (m, 2H, ArH), 3.22 (t, *J*=4.8 Hz, 4H, 2CH_2_ piperazine), 2.44 (t, *J*=4.8 Hz, 4H, 2CH_2_ piperazine), 2.22 (s, 3H, NCH_3_); ^13 ^C NMR (100 MHz, DMSO-d_6_) δ 161.1, 153.9, 152.3, 150.9, 140.5, 136.9, 135.5, 129.7, 129.7, 129.5, 129.3, 128.3, 127.8, 125.7, 122.0, 116.0, 114.8, 114.7, 112.0, 54.9, 47.7, 46.2; Anal. Calcd for C_26_H_26_N_6_O: C, 71.21; H, 5.98; N, 19.16, found C, 71.37; H, 6.12; N, 19.04; MS (ESI) *m/z*: 461 [M + Na^+^].

##### General procedure for the preparation of *N*'-(4–(4-substituted)benzylidene)-2-((4-oxo-3-aryl-3,4-dihydroquinazolin-2-yl)thio)acetohydrazide derivatives (6a-l)

To a mixture of acetohydrazide derivatives **5a,b** (5 mmol) and the appropriate aryloxybenzaldehydes or dialkylaminobenzaldehydes (5 mmol) in ethanol (50 mL), few drops of acetic acid were added, and the reaction mixture was refluxed for 6 h then cooled. The precipitate was filtered, washed with water crystallised from ethanol to give the corresponding acetohydrazide derivatives **6a-l**.

###### N'-(4–(4-chlorophenoxy)benzylidene)-2-((4-oxo-3-phenyl-3,4-dihydroquinazolin-2-yl)thio)acetohydrazide (6a)

White solid: 88% yield; mp 204–206 °C; IR (KBr, cm^−1^) 3448, 3194 (OH/NH), 3066 (CH aromatic), 2970, 2943 (CH aliphatic), 1681 (C=O), 1608 (C=N); ^1^H NMR (400 MHz, DMSO-d_6_) δ 11.73, 11.61 (2 s, 1H, OH/NH, D_2_O exchangeable), 8.24, 8.04 (2 s, 1H, CH=N), 8.10–8.07 (m, 1H, ArH), 7.86–7.77 (m, 1H, ArH), 7.73–7.70 (m, 2H, ArH), 7.61–7.58 (m, 3H, ArH), 7.51–7.44 (m, 6H, ArH), 7.11–7.04 (m, 4H, ArH), 4.46, 3.99 (2 s, 2H, CH_2_C=O); ^13 ^C NMR (100 MHz, DMSO-d_6_) δ 169.1, 163.9, 161.1, 161.1, 158.5, 158.3, 157.4, 157.2, 155.3, 155.2, 147.6, 146.5, 143.1, 136.3, 136.2, 135.4, 130.5, 130.4, 130.0, 130.0, 129.9, 129.5, 129.2, 128.4, 128.3, 127.1, 126.5, 121.5, 121.3, 120.0, 120.0, 119.1, 118.9, 35.9, 34.7; Anal. Calcd for C_29_H_21_ClN_4_O_3_S: C, 64.38; H, 3.91; N, 10.36, found C, 64.51; H, 4.17; N, 10.62; MS (ESI) *m/z*: 563 [M + Na^+^].

###### N'-(4–(4-bromophenoxy)benzylidene)-2-((4-oxo-3-phenyl-3,4-dihydroquinazolin-2-yl)thio) acetohydrazide (6b)

White solid: 82% yield; mp 224–226 °C; IR (KBr, cm^−1^) 3444, 3182 (OH/NH), 3074 (CH aromatic), 2954, 2912 (CH aliphatic), 1681 (C=O), 1608 (C=N); ^1^H NMR (400 MHz, DMSO-d_6_) δ 11.73, 11.61 (2 s, 1H, OH/NH, D_2_O exchangeable), 8.24, 8.04 (2 s, 1H, CH=N), 8.10–8.06 (m, 1H, ArH), 7.86–7.77 (m, 1H, ArH), 7.73–7.70 (m, 2H, ArH), 7.64–7.55 (m, 5H, ArH), 7.51–7.44 (m, 4H, ArH), 7.08–7.03 (m, 4H, ArH), 4.46, 3.99 (2 s, 2H, CH_2_C=O); ^13 ^C NMR (100 MHz, DMSO-d_6_) δ 169.2, 164.1, 161.2, 161.1, 158.4, 158.1, 157.4, 157.2, 155.8, 155.7, 147.5, 147.5, 146.5, 143.2, 136.2, 136.1, 135.4, 133.4, 130.5, 130.5, 130.1, 130.0, 130.0, 129.8, 129.5, 129.2, 127.1, 126.6, 126.5, 126.4, 121.9, 121.7, 119.9, 119.9, 119.2, 119.0, 116.3, 116.2, 35.8, 34.6; Anal. Calcd for C_29_H_21_BrN_4_O_3_S: C, 59.49; H, 3.62; N, 9.57, found C, 59.70; H, 3.85; N, 9.70; MS (ESI) *m/z*: 583 [M − H]^−^ and 585 [M + 2 − H]^−^.

###### N'-(4–(4-methoxyphenoxy)benzylidene)-2-((4-oxo-3-phenyl-3,4-dihydroquinazolin-2-yl)thio)acetohydrazide (6c)

Pale yellow solid: 83% yield; mp 128–130 °C; IR (KBr, cm^−1^) 3441, 3182 (OH/NH), 3070 (CH aromatic), 2947, 2912 (CH aliphatic), 1681 (C=O), 1604 (C=N); ^1^H NMR (400 MHz, DMSO-d_6_) δ 11.68, 11.57 (2 s, 1H, OH/NH, D_2_O exchangeable), 8.21, 8.01 (2 s, 1H, CH=N), 8.09–8.07 (m, 1H, ArH), 7.86–7.77 (m, 1H, ArH), 7.67–7.64 (m, 2H, ArH), 7.61–7.55 (m, 3H, ArH), 7.51–7.44 (m, 4H, ArH), 7.07–7.04 (m, 2H, ArH), 7.02–6.98 (m, 2H, ArH), 6.94 (t, *J*=8.8 Hz, 2H, ArH), 4.45, 3.99 (2 s, 2H, CH_2_C=O), 3.77, 3.76 (2 s, 3H, OCH_3_); ^13 ^C NMR (100 MHz, DMSO-d_6_) δ 169.1, 163.8, 161.1, 160.3, 160.0, 157.4, 157.2, 156.5, 149.0, 148.9, 147.6, 146.7, 143.3, 136.3, 136.2, 135.4, 130.5, 130.4, 130.0, 129.9, 129.4, 129.1, 128.9, 127.1, 126.5, 121.8, 121.7, 120.0, 120.0, 117.6, 117.5, 115.7, 55.9, 35.9, 34.7; Anal. Calcd for C_30_H_24_N_4_O_4_S: C, 67.15; H, 4.51; N, 10.44, found C, 67.38; H, 4.72; N, 10.62; MS (ESI) *m/z*: 559 [M + Na^+^].

###### N'-(4–(4-chlorophenoxy)benzylidene)-2-((3–(4-chlorophenyl)-4-oxo-3,4-dihydroquinazolin-2-yl)thio)acetohydrazide (6d)

White solid: 90% yield; mp 212–214 °C; IR (KBr, cm^−1^) 3433, 3178 (OH/NH), 3089 (CH aromatic), 2978, 2947 (CH aliphatic), 1678 (C=O), 1608 (C=N); ^1^H NMR (400 MHz, DMSO-d_6_) δ 11.72, 11.63 (2 s, 1H, OH/NH, D_2_O exchangeable), 8.24, 8.03 (2 s, 1H, CH=N), 8.09–8.06 (m, 1H, ArH), 7.86–7.78 (m, 1H, ArH), 7.73–7.66 (m, 4H, ArH), 7.61–7.54 (m, 2H, ArH), 7.49–7.45 (m, 4H, ArH), 7.11 (dd, *J*=8.8, 2.0 Hz, 2H, ArH), 7.06 (dd, *J*=8.4, 4.0 Hz, 2H, ArH), 4.47, 4.01 (2 s, 2H, CH_2_C=O); ^13 ^C NMR (100 MHz, DMSO-d_6_) δ 169.1, 159.2, 158.3, 155.3, 147.5, 135.5, 135.3, 135.2, 131.9, 130.5, 130.2, 130.1, 129.5, 129.2, 128.4, 128.3, 127.1, 126.5, 126.5, 121.5, 121.4, 119.1, 118.9, 35.5, 34.7; Anal. Calcd for C_29_H_20_Cl_2_N_4_O_3_S: C, 60.53; H, 3.50; N, 9.74, found C, 60.77; H, 3.68; N, 9.95; MS (ESI) *m/z*: 597 [M + Na^+^] and 599 [M + 2 + Na^+^].

###### N'-(4–(4-bromophenoxy)benzylidene)-2-((3–(4-chlorophenyl)-4-oxo-3,4-dihydroquinazolin-2-yl)thio)acetohydrazide (6e)

Pale yellow solid: 89% yield; mp 138–140 °C; IR (KBr, cm^−1^) 3448, 3182 (OH/NH), 3074 (CH aromatic), 2958, 2912 (CH aliphatic), 1681 (C=O), 1608 (C=N); ^1^H NMR (400 MHz, DMSO-d_6_) δ 11.71, 11.64 (2 s, 1H, OH/NH, D_2_O exchangeable), 8.24, 8.03 (2 s, 1H, CH=N), 8.09–8.03 (m, 1H, ArH), 7.86–7.78 (m, 1H, ArH), 7.73–7.66 (m, 4H, ArH), 7.61–7.54 (m, 4H, ArH), 7.52–7.44 (m, 2H, ArH), 7.08–7.03 (m, 4H, ArH), 4.47, 4.01 (2 s, 2H, CH_2_C=O); ^13 ^C NMR (100 MHz, DMSO-d_6_) δ 169.0, 163.8, 161.1, 161.1, 158.4, 158.2, 157.0, 156.8, 155.9, 155.8, 147.5, 146.5, 143.2, 135.4, 135.3, 135.2, 135.1, 133.4, 131.9, 130.1, 130.1, 130.1, 129.5, 129.2, 127.1, 126.6, 126.5, 121.9, 121.7, 120.0, 119.9, 119.2, 119.0, 116.3, 116.2, 35.9, 34.8; Anal. Calcd for C_29_H_20_BrClN_4_O_3_S: C, 56.19; H, 3.25; N, 9.04, found C, 56.34; H, 3.49; N, 9.31; MS (ESI) *m/z*: 617 [M − H]^−^ and 619 [M + 2 − H]^−^.

###### 2-((3–(4-chlorophenyl)-4-oxo-3,4-dihydroquinazolin-2-yl)thio)-N'-(4–(4-methoxy phenoxy)benzylidene)acetohydrazide (6f)

White solid: 82% yield; mp 234–236 °C; IR (KBr, cm^−1^) 3441, 3178 (OH/NH), 3070 (CH aromatic), 2970, 2939 (CH aliphatic), 1680 (C=O), 1608 (C=N); ^1^H NMR (400 MHz, DMSO-d_6_) δ 11.68, 11.58 (2 s, 1H, OH/NH, D_2_O exchangeable), 8.21, 8.00 (2 s, 1H, CH=N), 8.09–8.06 (m, 1H, ArH), 7.86–7.78 (m, 1H, ArH), 7.70–7.61 (m, 4H, ArH), 7.56 (t, *J*=8.0 Hz, 2H, ArH), 7.52–7.44 (m, 2H, ArH), 7.05 (d, *J*=8.8 Hz, 2H, ArH), 7.01–6.98 (m, 2H, ArH), 6.96–6.93 (m, 2H, ArH), 4.46, 4.00 (2 s, 2H, CH_2_C=O), 3.78, 3.77 (2 s, 3H, OCH_3_); ^13 ^C NMR (100 MHz, DMSO-d_6_) δ 169.0, 161.1, 160.1, 156.5, 149.0, 147.5, 143.4, 135.5, 135.2, 131.9, 130.1, 129.4, 129.1, 128.8, 127.1, 126.5, 121.8, 121.7, 119.9, 117.6, 117.5, 115.7, 55.9, 35.9, 34.7.; Anal. Calcd for C_30_H_23_ClN_4_O_4_S: C, 63.10; H, 4.06; N, 9.81, found C, 63.28; H, 4.29; N, 9.97; MS (ESI) *m/z*: 593 [M + Na^+^]. and 595 [M + 2 + Na^+^].

###### 2-((4-oxo-3-phenyl-3,4-dihydroquinazolin-2-yl)thio)-N'-(4-(piperidin-1-yl)benzylidene) acetohydrazide (6g)

Pale yellow solid: 78% yield; mp 164–166 °C; IR (KBr, cm^−1^) 3441, 3178 (OH/NH), 3074 (CH aromatic), 2943, 2858 (CH aliphatic), 1681 (C=O), 1604 (C=N); ^1^H NMR (400 MHz, DMSO-d_6_) δ 11.50, 11.39 (2 s, 1H, OH/NH, D_2_O exchangeable), 8.10 (d, *J*=2.4 Hz, 1H, ArH), 8.08, 7.92 (2 s, 1H, CH=N), 7.85–7.78 (m, 1H, ArH), 7.62–7.59 (m, 3H, ArH), 7.53–7.45 (m, 6H, ArH), 6.93 (t, *J*=8.0 Hz, 2H, ArH), 4.45, 3.97 (2 s, 2H, CH_2_C=O), 3.24 (brs, 4H, 2CH_2_ piperidine), 1.57 (brs, 6H, 3CH_2_ piperidine); ^13 ^C NMR (100 MHz, DMSO-d_6_) δ 168.8, 163.6, 161.2, 161.2, 157.4, 157.2, 152.8, 152.7, 147.8, 147.5, 144.5, 136.2, 136.1, 135.4, 130.5, 130.5, 130.1, 129.8, 128.9, 128.5, 127.0, 126.6, 126.5, 123.6, 123.4, 119.9, 115.1, 115.0, 48.9, 35.8, 34.7, 25.4, 24.4; Anal. Calcd for C_28_H_27_N_5_O_2_S: C, 67.58; H, 5.47; N, 14.07, found C, 67.40; H, 5.69; N, 14.31; MS (ESI) *m/z*: 520 [M + Na^+^].

###### N'-(4-morpholinobenzylidene)-2-((4-oxo-3-phenyl-3,4-dihydroquinazolin-2-yl)thio)acetohydrazide (6h)

White solid: 91% yield; mp 236–242 °C; IR (KBr, cm^−1^) 3479, 3224 (OH/NH), 3062 (CH aromatic), 2954, 2916 (CH aliphatic), 1681 (C=O), 1604 (C=N); ^1^H NMR (400 MHz, DMSO-d_6_) δ 11.53, 11.43 (2 s, 1H, OH/NH, D_2_O exchangeable), 8.12, 7.93 (2 s, 1H, CH=N), 8.08 (d, *J*=7.6 Hz, 1H, ArH), 7.86–7.79 (m, 1H, ArH), 7.62–7.59 (m, 4H, ArH), 7.55–7.52 (m, 2H, ArH), 7.50–7.45 (m, 3H, ArH), 6.97 (t, *J*=7.6 Hz, 2H, ArH), 4.45, 3.97 (2 s, 2H, CH_2_C=O), 3.73 (q, *J*=4.8 Hz, 4H, 2CH_2_ morpholine), 3.20 (t, *J*=4.8 Hz, 4H, 2CH_2_ morpholine); ^13 ^C NMR (100 MHz, DMSO-d_6_) δ 168.8, 163.6, 161.1, 157.5, 152.7, 152.5, 147.6, 144.2, 136.3, 136.2, 135.4, 130.4, 130.0, 129.9, 128.8, 128.4, 127.1, 126.5, 124.8, 124.7, 119.9, 114.9, 114.8, 66.4, 48.0, 47.9, 35.8, 34.7; Anal. Calcd for C_27_H_25_N_5_O_3_S: C, 64.91; H, 5.04; N, 14.02; found C, 64.75; H, 5.23; N, 14.29; MS (ESI) *m/z*: 522 [M + Na^+^].

###### N'-(4–(4-methylpiperazin-1-yl)benzylidene)-2-((4-oxo-3-phenyl-3,4-dihydroquinazolin-2-yl)thio) acetohydrazide (6i)

Pale yellow solid: 82% yield; mp 120–122 °C; IR (KBr, cm^−1^) 3441, 3163 (OH/NH), 3074 (CH aromatic), 2943, 2862 (CH aliphatic), 1681 (C=O), 1604 (C=N); ^1^H NMR (400 MHz, DMSO-d_6_) δ 11.53, 11.42 (2 s, 1H, OH/NH, D_2_O exchangeable), 8.12, 7.93 (2 s, 1H, CH=N), 8.08 (d, *J*=7.2 Hz, 1H, ArH), 7.88–7.76 (m, 2H, ArH), 7.59 (brs, 4H, ArH), 7.52–7.48 (m, 4H, ArH), 6.95–6.94 (m, 2H, ArH), 4.45, 3.97 (2 s, 2H, CH_2_C=O), 3.23 (brs, 4H, 2CH_2_ piperazine), 2.45 (brs, 4H, 2CH_2_ piperazine), 2.22 (s, 3H, NCH_3_); ^13 ^C NMR (100 MHz, DMSO-d_6_) δ 172.6, 168.8, 163.5, 161.2, 161.1, 157.5, 157.3, 152.5, 152.4, 147.6, 144.3, 136.3, 136.2, 135.4, 130.5, 130.4, 130.0, 129.9, 128.8, 128.4, 127.1, 126.5, 126.4, 124.4, 124.3, 120.0, 115.0, 114.9, 54.8, 47.5, 47.4, 46.1, 35.9, 34.8, 21.6; Anal. Calcd for C_28_H_28_N_6_O_2_S: C, 65.60; H, 5.51; N, 16.39; found C, 65.43; H, 5.67; N, 16.58.

###### 2-((3–(4-chlorophenyl)-4-oxo-3,4-dihydroquinazolin-2-yl)thio)-N'-(4-(piperidin-1-yl)benzylidene) acetohydrazide (6j)

Pale yellow solid: 85% yield; mp 242–244 °C; IR (KBr, cm^−1^) 3448, 3178 (OH/NH), 3089 (CH aromatic), 2935, 2854 (CH aliphatic), 1681 (C=O), 1604 (C=N); ^1^H NMR (400 MHz, DMSO-d_6_) δ 11.49, 11.41 (2 s, 1H, OH/NH, D_2_O exchangeable), 8.09 (s, 1H, ArH), 8.07, 7.90 (2 s, 1H, CH=N), 7.86–7.80 (m, 1H, ArH), 7.68 (dd, *J*=8.4, 2.8 Hz, 2H, ArH), 7.62–7.54 (m, 3H, ArH), 7.51–7.46 (m, 3H, ArH), 6.96–6.92 (m, 2H, ArH), 4.45, 3.99 (2 s, 2H, CH_2_C=O), 3.26 (brs, 4H, 2CH_2_ piperidine), 1.58 (brs, 6H, 3CH_2_ piperidine); ^13 ^C NMR (100 MHz, DMSO-d_6_) δ 168.6, 163.3, 161.1, 161.1, 157.1, 156.9, 152.8, 152.7, 147.8, 147.5, 144.4, 135.5, 135.2, 135.2, 135.2, 135.1, 131.9, 130.1, 130.1, 128.9, 128.5, 127.1, 126.6, 126.5, 123.6, 123.5, 119.9, 115.1, 115.0, 48.9, 48.8, 35.9, 34.8, 25.4, 24.4.; Anal. Calcd for C_28_H_26_ClN_5_O_2_S: C, 63.21; H, 4.93; N, 13.16; found C, 63.40; H, 5.19; N, 13.46; MS (ESI) *m/z*: 554 [M + Na^+^].

###### 2-((3–(4-chlorophenyl)-4-oxo-3,4-dihydroquinazolin-2-yl)thio)-N'-(4-morpholino benzylidene) acetohydrazide (6k)

White solid: 75% yield; mp 240–242 °C; IR (KBr, cm^−1^) 3444, 3255 (OH/NH), 3097 (CH aromatic), 2962, 2858 (CH aliphatic), 1666 (C=O), 1604 (C=N); ^1^H NMR (400 MHz, DMSO-d_6_) δ 11.53, 11.45 (2 s, 1H, OH/NH, D_2_O exchangeable), 8.12, 7.93 (2 s, 1H, CH=N), 8.08 (d, *J*=8.0 Hz, 1H, ArH), 7.86–7.79 (m, 1H, ArH), 7.69–7.67 (m, 2H, ArH), 7.57–7.55 (m, 2H, ArH), 7.54–7.50 (m, 3H, ArH), 7.48–7.46 (m, 1H, ArH), 6.99–6.96 (m, 2H, ArH), 4.46, 3.99 (2 s, 2H, CH_2_C=O), 3.73 (q, *J*=4.8 Hz, 4H, 2CH_2_ morpholine), 3.20 (t, *J*=4.8 Hz, 4H, 2CH_2_ morpholine); ^13 ^C NMR (100 MHz, DMSO-d_6_) δ 168.7, 163.4, 161.1, 157.2, 152.7, 152.5, 147.5, 144.2, 135.5, 135.2, 135.2, 131.9, 130.1, 128.8, 128.4, 127.1, 126.5, 124.8, 119.9, 114.9, 114.8, 66.4, 48.0, 47.9, 40.6, 35.9, 34.8; Anal. Calcd for C_27_H_24_ClN_5_O_3_S: C, 60.72; H, 4.53; N, 13.11; found C, 60.98; H, 4.31; N, 13.40; MS (ESI) *m/z*: 532 [M -H]^−^ and 534 [M + 2 -H]^−^.

###### 2-((3–(4-chlorophenyl)-4-oxo-3,4-dihydroquinazolin-2-yl)thio)-N'-(4–(4-methylpiperazin-1-yl) benzylidene)acetohydrazide (6l):

White solid: 86% yield; mp 204–206 °C; IR (KBr, cm^−1^) 3444, 3167 (OH/NH), 3078 (CH aromatic), 2939, 2843 (CH aliphatic), 1674 (C=O), 1604 (C=N); ^1^H NMR (400 MHz, DMSO-d_6_) δ 11.52, 11.43 (2 s, 1H, OH/NH, D_2_O exchangeable), 8.11, 7.92 (2 s, 1H, CH=N), 8.08 (d, *J*=7.6 Hz, 1H, ArH), 7.86–7.79 (m, 1H, ArH), 7.68 (dd, *J*=8.4, 2.4 Hz, 2H, ArH), 7.57–7.54 (m, 2H, ArH), 7.53–7.45 (m, 4H, ArH), 6.97–6.94 (m, 2H, ArH), 4.46, 3.99 (2 s, 2H, CH_2_C=O), 3.23 (brs, 4H, 2CH_2_ piperazine), 2.45–2.43 (m, 4H, 2CH_2_ piperazine), 2.22, 2.21 (2 s, 3H, NCH_3_); ^13 ^C NMR (100 MHz, DMSO-d_6_) δ 168.7, 163.3, 161.1, 161.1, 157.2, 156.9, 152.6, 152.4, 147.6, 147.5, 144.3, 135.5, 135.2, 135.2, 135.1, 131.9, 130.1, 130.1, 128.8, 128.4, 127.1, 126.6, 126.5, 124.3, 124.2, 120.0, 119.9, 115.0, 114.9, 54.9, 47.6, 47.5, 46.2, 35.9, 34.9; Anal. Calcd for C_28_H_27_ClN_6_O_2_S: C, 61.47; H, 4.97; N, 15.36; found C, 61.39; H, 5.13; N, 15.58; MS (ESI) *m/z*: 547 [M + H^+^] and 549 [M + 2 + H^+^].

## Biological evaluation

### Biological assays

The biological assays were carried out according to the previously reported procedures and have been provided in the Supplementary Materials; antiproliferative activity screening and five dose concentrations assay by NCI^34–37^, *in vitro* EGFR inhibitory assay^38^, cell cycle analysis^39^, apoptosis assay^40^, caspase-3 enzyme assay^41^.

### Molecular modelling studies

The crystallographic structure of EGFR protein (PDB: 1M17) was obtained from the protein data bank website, (http://www.pdb.org) with resolution of 2.60 Å. All the molecular modelling studies were carried out using Molecular Operating Environment (MOE 2020.09; Chemical Computing Group, Canada) as the computational software. The hydrogen atoms were added, the protonation states of the amino acid residues were assigned, and the partial charges of atoms were added using Protonate 3D algorithm. Compounds were modelled using MOE builder, and the structure was energy minimised using the MMFF94x force field. Using the MOE induced-fit Dock tool, docking studies of the synthesised compound into the active site was done and the final docked complexes of ligand–enzyme was selected according to the criteria of binding energy score combined with geometrical matching quality.

### Statistical analysis

Data are represented as mean ± *SD*. Significant differences between groups were analysed by using Graphpad Prism 9.1.0. Differences were considered significant at *p* < 0.05.

## Result and discussion

### Chemistry

**Scheme 1.**
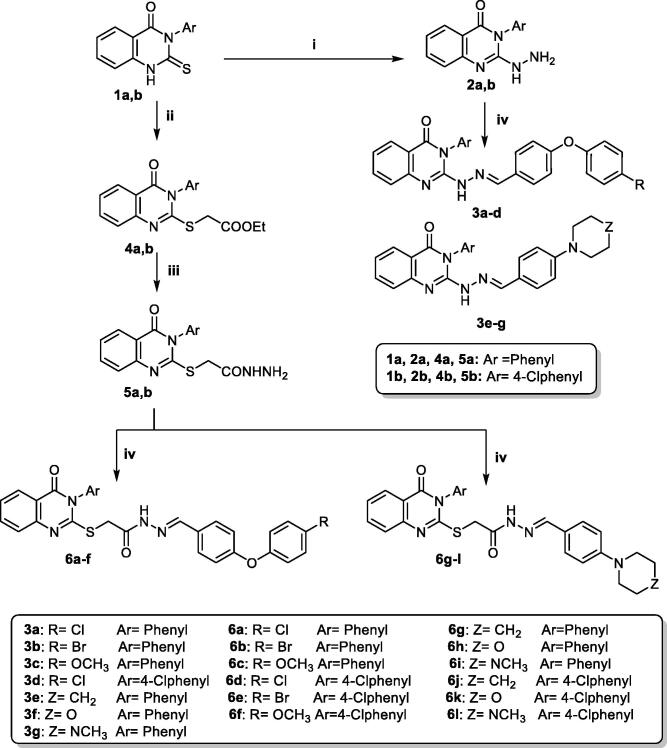
Synthesis of quinazolin-4(*3H*)-one derivatives **3a–g** and **6a–l**. **Reagents and conditions:** (i) NH_2_NH_2_.H_2_O, ethanol, reflux, 12 h; (ii) Ethyl bromooacetate, dry acetone, anhydrous K_2_CO_3_, RT, 8 h; (iii) NH_2_NH_2_.H_2_O, ethanol, reflux, 6 h; (iv) 4-Substituted benzaldehyde, ethanol, drops of glacial acetic acid, reflux, 6 h.

Nineteen new quinazolin-4(3*H*)-one derivatives **3a–g** and **6a–l** were synthesised according to Scheme 1. The key starting compounds 3-phenyl-2-thioxo-2,3-dihydroquinazolin-4(*1H*)-one (**1a**) and 3–(4-chlorophenyl)-2-thioxo-2,3-dihydroquinazolin-4(*1H*)-one (**1b**) were obtained in good yields by reacting anthranilic acid with the appropriate aryl isothiocyanate in refluxing ethanol in presence of triethylamine as catalyst^30^^,^^31^. Compounds **1a,b** were then treated with hydrazine hydrate 99% as reported to yield 2-hydrazinyl-3-phenylquinazolin-4(*3H*)-one (**2a**) and 3–(4-chlorophenyl)-2-hydrazinylquinazolin-4(*3H*)-one (**2b**)^31^. Furthermore, heating the obtained hydrazine derivatives **2a,b** with 4-substituted benzaldehyde derivatives in ethanol as solvent in the presence of few drops of glacial acetic acid^42^ afforded the novel target compounds **3a–g**. The ^1^H NMR spectra of the compounds **3a–g** showed D_2_O exchangeable singlet signal due to NH at the range δ 10.49–10.61 ppm. Moreover, singlet signal at the range δ 7.93–8.09 ppm was present due to the characteristic azomethine protons.

**Figure 3.**
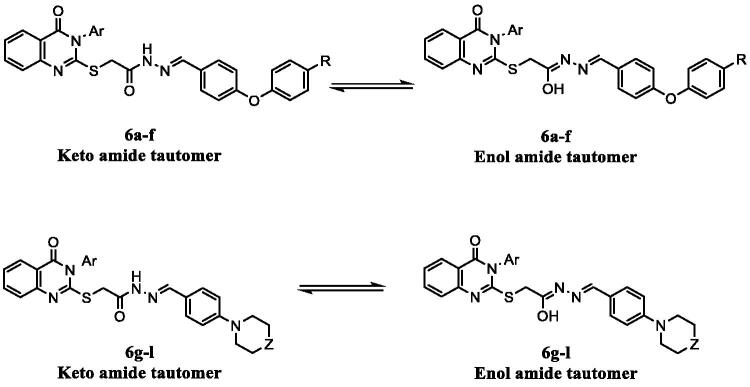
Tautomerism in hydrazone derivatives **6a–f** and **6g–l**.

On the other hand, alkylation of 2-thioxo-2,3-dihydroquinazolin-4(*1H*)-one derivatives **1a,b** with ethyl bromoacetate at room temperature in dry acetone containing anhydrous potassium carbonate afforded ethyl 2-((4-oxo-3-aryl-3,4-dihydroquinazolin-2-yl)thio)acetate **4a,b**^32^^,^^33^. The ester derivatives **4a,b** were further reacted with hydrazine hydrate 99% to afford the corresponding acetohydrazide derivatives 2-((4-oxo-3-aryl-3,4-dihydroquinazolin-2-yl)thio)acetohydrazide **5a,b**^32^^,^^33^. Compounds **5a,b** were finally reacted with 4-substituted benzaldehyde derivatives to yield the desired hydrazone derivatives **6a–l**. The ^1^H NMR spectra of derivatives **6a–l** revealed that the hydrazones existed in a keto amide/enol amide mixture (Figure 3)^43^. ^1^H NMR spectra of series **6a–l** showed two singlet signals with an integration of 2H due to S**CH_2_**C=O protons at the ranges of δ 4.45–4.47 ppm and δ 3.97–4.01 ppm corresponding to keto amide and enol amide tautomers, respectively. Moreover, two singlet signals with an integration of 1H in ^1^H NMR spectra of **6a–l** appeared due to the azomethine protons of both tautomers at the ranges of δ 8.07–8.24 and δ 7.90–8.04 ppm. Additionally, two D_2_O exchangeable peaks due to OH proton in enol and NH in keto tautomers with 1H total integration appeared at the ranges of δ 11.49–11.73 ppm and δ 11.39–11.64 ppm, respectively. On the other hand, the appearance of the OCH_3_ group in derivatives **6c** and **6f** as two singlet signals with total integration of 3H at δ 3.77–3.78 ppm and δ 3.76–3.77 ppm is in concordance with the presence of tautomerism. ^13 ^C NMR spectra of derivatives **6a–l** showed two peaks at the ranges δ 35.8–35.9 ppm and δ 34.6–34.9 ppm corresponding to S**C**H_2_C=O in enol and keto tautomers, respectively.

## Biological evaluation

### Evaluation of antiproliferative activity against a panel of 60 human cancer cell lines

Two series of substituted quinazolinone derivatives **3a–g** and **6a–l** were evaluated for their antiproliferative activity at a single dose (10 μM) using 60 human cancer cell lines, by the National Cancer Institute (NCI), USA. The screening was achieved under the Developmental Therapeutic Program (DTP)^34–37^. NCI cell lines include leukaemia, melanoma, and cancers of the breast, kidney, ovarian, colon, central nervous system (CNS), prostate, and non-small cell (NSC) lung. The growth inhibition percentage (GI%) representing the *in vitro* antiproliferative activity was illustrated in Table 1.

**Table 1.** *In vitro* growth inhibition % (GI%) of the synthesised compounds **6a–6l** against a panel of 60 tumour cell lines at 10 μM.

Subpanel	6a	6b	6c	6d	6e	6f	6g	6h	6i	6j	6k	6l
Leukaemia												
CCRF-CEM	49.57	43.1	–	> 100	–	36.46	–	–	–	–	–	–
HL-60(TB)	–	–	–	–	–	–	–	–	–	–	–	–
K-562	38.79	37.52	–	**77.02**	–	41.57	26.76	–	–	26.42	–	–
MOLT-4	41.34	44.47	–	> 100	–	38.29	28.47	–	–	–	–	–
RPMI-8226	26.75	31.73	–	**75.28**	–	14.91	17.56	–	–	–	–	–
SR	29.66	26.64	–	67.08	–	27.19	24.73	–	–	25.23	–	–
NSC lung cancer												
A549/ATCC	58.27	**57.81**	–	**83.53**	–	52.97	15.14	–	–	50.65	–	–
EKVX	40.95	39.26	–	59.16	–	30.59	20.43	–	–	17.94	–	–
HOP-62	40.92	36.11	–	67.44	–	26.68	–	–	–	**81.63**	–	–
HOP-92	33.3	26.96	–	**97.41**	–	22.66	19.49	18.69	–	37.67	–	–
NCI-H226	27.88	22.43	–	24.44	–	20.29	–	–	–	61.77	–	–
NCI-H322M	15.51	–	–	–	–	15.35	–	–	–	23.03	–	–
NCI-H460	67.86	**71.98**	–	> 100	–	**88.8**	–	–	–	**90.26**	–	–
NCI-H522	50.38	51.53	–	**79.34**	13.4	48.7	15.3	–	–	–	–	–
Colon cancer												
COLO-205	50.76	55.61	–	**85.4**	–	27.18	–	–	–	17.87	–	–
HCC-2998	–	–	–	**79.7**	–	16.65	–	–	–	–	–	–
HCT-116	**94.38**	**92.19**	–	> 100	–	**86.66**	15.51	–	–	63.1	–	–
HCT-15	39.16	36.28	–	**89.62**	–	43.95	16.21	–	–	32.2	–	–
HT29	40.85	61.82	–	**98.24**	–	**83.93**	–	–	–	39.1	–	–
KM12	16.31	23.73	–	**82.6**	–	22.83	–	–	–	3.59	–	–
SW-620	56.93	**70.37**	–	**97.38**	–	55.6	–	–	–	34.12	–	–
CNS cancer												
SF-268	–	15.72	–	44.24	–	42.06	–	–	–	65.26	–	–
SF-295	16.22	16.28	–	35.28	–	32.32	–	–	–	23.56	–	–
SF-539	31.48	31.3	–	30.33	–	67.83	–	–	–	58.67	–	–
SNB-19	66.78	60.15	–	**71.51**	–	38.45	–	–	–	56.75	–	–
SNB-75	39.91	42.17	–	42.06	–	58.37	19.86	–	–	20.14	–	–
U251	**84.83**	**78.53**	–	> 100	–	63.53	–	–	–	49.41	–	–
Melanoma												
LOX IMIV	**86.02**	**79.01**	–	> 100	–	**85.33**	–	–	–	56.49	–	15.15
MALME-3M	48.74	45.07	–	> 100	–	36.13	–	–	–	35.79	–	–
M14	51.16	43.73	NT	> 100	NT	NT	–	NT	NT	NT	NT	NT
MDA-MB-435	15.38	19.23	–	**74.95**	–	–	–	–	–	–	–	–
SK-MEL-2	13.13	–	–	45.68	–	–	–	–	–	–	–	–
SK-MEL-28	37.58	29.11	–	**96.83**	–	–	–	–	–	15.16	–	–
SK-MEL-5	28.84	40.93	–	**89.11**	–	21.98	–	–	–	–	–	–
UACC-257	–	–	–	41.0	–	–	–	–	–	–	–	–
UACC-62	33.42	31.24	–	**99.57**	–	38.11	–	–	–	26.53	–	–
Ovarian cancer												
IGROV1	50.89	34.45	–	**79.42**	–	51.35	–	–	–	44.51	–	–
OVCAR-3	–	–	–	–	–	> 100	–	–	–	67.19	–	–
OVCAR-4	26.25	18.26	–	**98.3**	–	27.45	–	–	–	41.44	–	–
OVCAR-5	18.55	–	–	29.36	–	18.76	–	–	–	23.12	–	–
OVCAR-8	40.84	36.67	–	**86.57**	–	–	–	–	–	34.7	–	–
NCI/ADR-RES	47.27	44.83	–	**84.26**	–	48.89	–	–	–	60.15	–	–
SK-OV-3	16.8	15.49	–	66.17	–	33.7	–	–	–	69.91	–	–
Renal cancer												
786-0	46.79	54.01	–	> 100	–	**74.84**	–	–	–	**92.8**	–	–
A498	34.29	17.71	17.18	> 100	15.7	27.11	–	15.82	10.22	21.52	–	32.61
ACHN	25.52	–	–	36.52	–	**92.17**	–	–	–	**82.62**	–	–
CAKI-1	40.81	29.24	NT	66.99	NT	NT	21.97	NT	NT	NT	NT	NT
RXF 393	57.96	37.84	NT	> 100	NT	NT	–	NT	NT	NT	NT	NT
SN12C	**73.08**	59.26	–	54.75	–	47.29	–	–	–	37.62	–	–
TK-10	–	–	–	60.33	–	–	–	–	–	–	–	–
UO-31	18.82	–	–	–	20.82	52.26	23.31	–	–	46.97	–	–
Prostate cancer												
PC-3	41.33	28.25	–	**99.2**	–	36.32	18.52	–	–	21.44	–	–
DU-145	61.56	61.67	–	**86.91**	–	50.84	–	–	–	**70.89**	–	–
Breast cancer												
MCF7	35.46	31.12	–	**84.47**	–	50.72	16.22	–	–	32.51	–	–
MDA-MB-231/ATCC	50.77	49.15	–	69.61	–	53.27	—	–	–	28.05	–	–
HS 578T	41.12	39.2	–	**78.62**	–	> 100	–	–	–	62.12	–	–
BT-549	30.09	26.93	–	> 100	–	–	–	–	–	–	–	–
T-47D	17.5	17.61	–	69.86	–	62.15	–	–	–	25.43	–	–
MDA-MB-468	28.41	34.89	–	> 100	–	33.16	–	–	–	20.54	–	–
Mean	**37.29**	**34.78**	**< 0**	**80.23**	**< 0**	**42.46**	**4.28**	**< 0**	**< 0**	**34.19**	**< 0**	**< 0**

–: growth inhibition below 15%.; NT: not tested.

Bold numbers: growth inhibition above 70%.

The series **3a–g**, representing 2-(substitutedbenzylidene)hydrazinyl quinazolin-4(*3H*)-ones showed low to no activity against most of the investigated cancer cell lines (Supplementary data). In contrast, five derivatives of the acetohydrazide series **6a–l** displayed potent broad-spectrum antiproliferative activity against most of the examined cancer cell lines with mean growth percentage of range 34.19 − 80.23%.

4-chlorophenoxybenzylidene acetohydrazide derivative **6a** exhibited broad-spectrum antiproliferative activity with mean GI% of 37.29. It showed significantly potent antiproliferative activity against colon cancer HCT-116 (GI% 94.38); CNS cancer U251 (GI% 84.83); melanoma LOX IMIV (GI% 86.02) and renal cancer SN12C (GI% 73.08). It had also demonstrated excellent-to-moderate antiproliferative activity against 33 cancer cell lines with GI% in the range of 67.86% to 30.09%. Moreover, compound **6b** with 4-bromophenoxybenzylidene acetohydrazide demonstrated broad-spectrum antiproliferative activity with mean growth inhibition of 34.78%. It showed significantly potent antiproliferative activity against NSC lung cancer NCI-H460; colon cancer (HCT-116 and SW-620); CNS cancer U251 and melanoma LOX IMIV with GI% 71.98%, 92.19%, 70.37%, 78.53% and 79.01%, respectively. It revealed excellent-to-moderate antiproliferative activity against 29 cancer cell lines with growth inhibition in the range of 61.82–31.2%.

Compound **6c** with 4-methoxyphenoxy substitution showed no activity against all tested cancer cell lines. Compound **6d** with (4-chlorophenoxy)benzylidene)-3–(4-chlorophenyl)quinazolinone demonstrated significantly potent to excellent broad-spectrum antiproliferative activity with mean GI% 80.23. It was accordingly evaluated at five dose concentrations by NCI to define its dose-response behaviour and determine its GI_50_, TGI, and LC_50_ values. (Values were represented in Table 2). Compound **6d** showed significantly potent antiproliferative activity against 24 cancer cell lines with GI% ranging between 99.57% and 71.51%. Also, compound **6d** showed significantly potent antiproliferative activity with inhibition percentage above 90% with NSC lung cancer HOP-92 (GI% 97.41); colon cancer (HT29 and SW-620 with GI% 98.24 and 97.38, respectively); melanoma (SK-MEL-28 and UACC-62 with GI% 96.83 and 99.57 respectively); ovarian cancer OVCAR-4 (GI% 98.3) and prostate cancer PC-3 (GI% 99.2). Additionally, compound **6d** displayed lethal activity against 13 of the tested cancer cell lines with growth inhibition above 100%.

**Table 2.** The values of GI_50_, TGI and LC_50_ of compound **6d** at 5 doses against 60 cell line panel.

	6d		
Compound	GI_50_	TGI	LC_50_
Leukaemia			
CCRF-CEM	3.09	48.5	>100
HL-60(TB)	16.0	37.0	85.7
K-562	4.15	20.2	>100
MOLT-4	2.29	9.23	44.7
RPMI-8226	2.98	15.8	>100
SR	1.84	6.86	31.9
NSC lung cancer			
A549/ATCC	3.25	14.0	83.3
EKVX	3.1	13.9	63.6
HOP-62	1.69	4.05	9.69
HOP-92	2.07	5.62	22.8
NCI-H226	2.46	6.59	33.2
NCI-H322M	3.02	15.0	90.8
NCI-H460	**0.789**	2.6	7.78
NCI-H522	2.34	8.31	42.6
Colon cancer			
COLO 205	2.79	8.74	91.6
HCC-2998	1.92	3.81	7.57
HCT-116	1.55	3.74	9.03
HCT-15	2.41	7.48	28.5
HT29	2.14	11.1	>100
KM12	2.04	5.0	26.6
SW-620	1.81	7.84	42.4
CNS cancer			
SF-268	3.85	23.9	>100
SF-295	3.61	13.3	36.4
SF-539	2.54	6.32	21.5
SNB-19	3.36	12.7	53.2
SNB-75	3.99	29.2	>100
U251	1.65	3.69	8.23
Melanoma			
LOX IMVI	1.29	2.59	5.2
MALME-3M	1.38	2.77	5.55
M14	1.58	3.47	7.58
MDA-MB-435	1.93	4.56	13.0
SK-MEL-2	2.86	8.93	41.3
SK-MEL-28	1.51	3.01	6.01
SK-MEL-5	1.75	3.51	7.07
UACC-257	4.62	22.5	>100
UACC-62	1.59	3.18	6.38
Ovarian cancer			
IGROV1	1.51	3.28	7.12
OVCAR-3	2.75	9.55	38.9
OVCAR-4	1.89	4.57	17.5
OVCAR-5	2.27	6.44	32.2
OVCAR-8	2.41	6.56	>100
NCI/ADR-RES	1.64	3.56	7.76
SK-OV-3	3.26	11.9	49.4
Renal cancer			
786-0	2.51	6.41	26.5
A498	1.94	4.34	9.74
ACHN	4.36	15.2	39.0
CAKI-1	3.06	12.0	35.4
RXF 393	1.7	3.14	5.81
SN12C	1.74	4.14	9.84
TK-10	5.97	40.2	>100
UO-31	7.47	21.1	47.1
Prostate cancer			
PC-3	2.58	9.32	>100
DU-145	3.52	20.4	>100
Breast cancer			
MCF7	2.01	5.5	25.6
MDA-MB-231/ATCC	1.79	3.9	8.51
HS 578T	2.17	6.36	>100
BT-549	3.28	12.2	36.2
T-47D	2.09	5.76	49.5
MDA-MB-468	2.08	5.08	18.8

Replacing 4-chlorophenoxy with 4-bromophenoxy in derivative **6e** resulted in abolishing the antiproliferative activity. On the other hand, derivative **6f** with 4-methoxyphenoxy substitution displayed antiproliferative activity (mean GI% of 42.46). Compound **6f** exhibited significantly potent antiproliferative activity against 6 cancer cell lines representing NSC lung cancer NCI-H460; colon cancer (HCT-116 and HT29); melanoma LOX IMIV and renal cancer (786–0 and ACHN) with GI% 88.8%, 86.66%, 83.93%, 85.33%, 74.84% and 92.17%, respectively. It also showed moderate activity against 27 cell lines with growth inhibition percentage ranging from 67.83 to 30.59%. In addition, compound **6f** showed lethal activity against ovarian cancer OVCAR-3 and breast cancer HS 578 T with growth inhibition above 100%.

Derivative **6j** with *N'*-(4-(piperidin-1-yl)benzylidene)-3–(4-chlorophenyl)-quinazolinone acetohydrazide showed antiproliferative activity with mean GI% of 34.19. It showed significantly potent antiproliferative activity against 5 cell lines including NSC lung cancer (HOP-62 and NCI-H460); renal cancer (786–0 and ACHN) and prostate DU-145 with GI% 81.63%, 90.26%, 92.8%, 82.62% and 70.89% respectively. It showed moderate antiproliferative activity against 23 cell lines with GI% range of 69.91–32.2%. Finally, derivatives **6 h**, **6i**, **6k** and **6 l** did not show activity against all cell lines.

**Figure 4.**
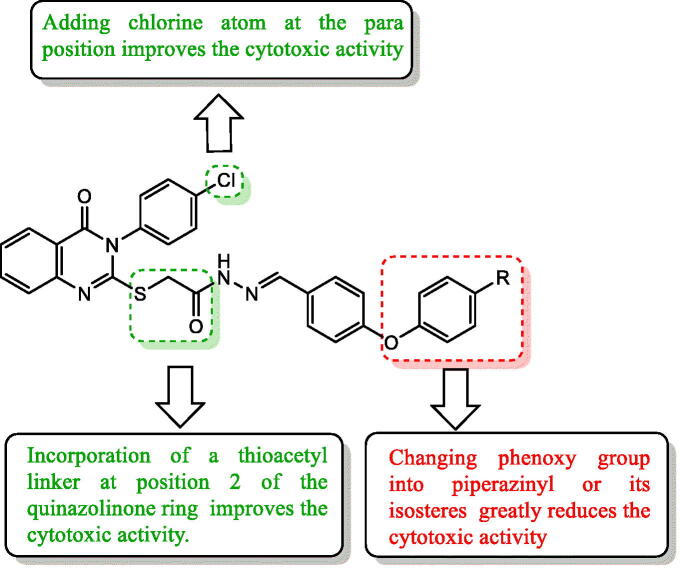
Structure activity relationship for synthesised quinazolinone derivatives correlated with their cytotoxic activity.

The most remarkable effects to be concluded in the structure variations of the synthesised derivatives and their antiproliferative activity are represented in Figure 4. The incorporation of thioacetyl linker at position 2 of the quinazolinone ring was found to be essential for the antiproliferative activity as most of the synthesised compounds with thioacetyl bridge in series **6a–l** were found to have of high anticancer activity while compounds **3a–g**, lacking the thioacetyl bridge, showed no promising results. This may be due to the increased flexibility introduced by thioacetyl spacer, which enables the compounds to fit better into the receptor^44^.

In series **6a–l**, general better antiproliferative activity were displayed by compounds bearing chloro atom at the para-position of the quinazolinone 3-phenyl ring as demonstrated by the relatively high GI percentage values of compounds **6d** (mean GI% = 80.23%), **6f** (mean GI% = 42.46%) and **6j** (mean GI% = 34.19%) over their unsubstituted analogues **6a (**mean GI% = 37.29%), **6c** (mean GI% = 0%) and **6 g** (mean GI% = 4.28%), respectively. This observation was in accordance with the previously reported study in other related compounds^45^. It was also observed that the substituted phenoxy derivatives **6a–f** have generally better GI% values than the corresponding piperazinyl (or its isostere) aryl group **6g–l**.

### Evaluation of in vitro antiproliferative activity of compound 6d at 5 dose concentrations

Compound **6d** was found to be the most effective anticancer agent in this study based on preliminary single dose (10 μM) screening results. It demonstrated promising efficacy against a variety of cancer cell lines, with mean growth inhibition of 80.23% (Table 1). Accordingly, compound **6d** was further subjected to five dose concentrations assay (0.01, 0.1, 1, 10 and 100 μM) to detect its dose-response behaviour and calculate the values of GI_50_ (the dose that inhibit 50% of cell growth in comparison to control), TGI (the dose that completely inhibit growth), and LC_50_ (the dose that kill 50% of the cells). The values are presented in Table 2. The obtained results showed that, compound **6d** displayed superior sub-micromolar activity towards NSC lung cancer cell line NCI-H460 with GI_50_= 0.789 µM, however it showed high lethality with this cell line (LC_50_= 7.78 µM). Compound **6d** exhibited also potent and broad-spectrum antiproliferative activity against most of the tested cancer cell lines, with GI_50_ values in the range of 1.29–5.97 µM, in addition to moderate antiproliferative activity against leukaemia cell line HL-60(TB) with GI_50_= 16.0 µM. Compound **6d** also exhibited high cytostatic activity (TGI range: 2.59–9.55 µM) against 40 cancer cell lines. Moreover, **6d** demonstrated good to moderate cytostatic activity against the rest of cell lines with TGI range of 11.1–48.5 µM. Compound **6d** exhibited remarkable differences between its cytotoxic indicator (LC_50_) and cytostatic markers (GI_50_ and TGI) against colon cancer HT29; ovarian cancer OVCAR-8; prostate cancer PC-3 and breast cancer HS 578T, which indicates a wide therapeutic index.

### In vitro EGFR kinase inhibitory assay

**Figure 5.**
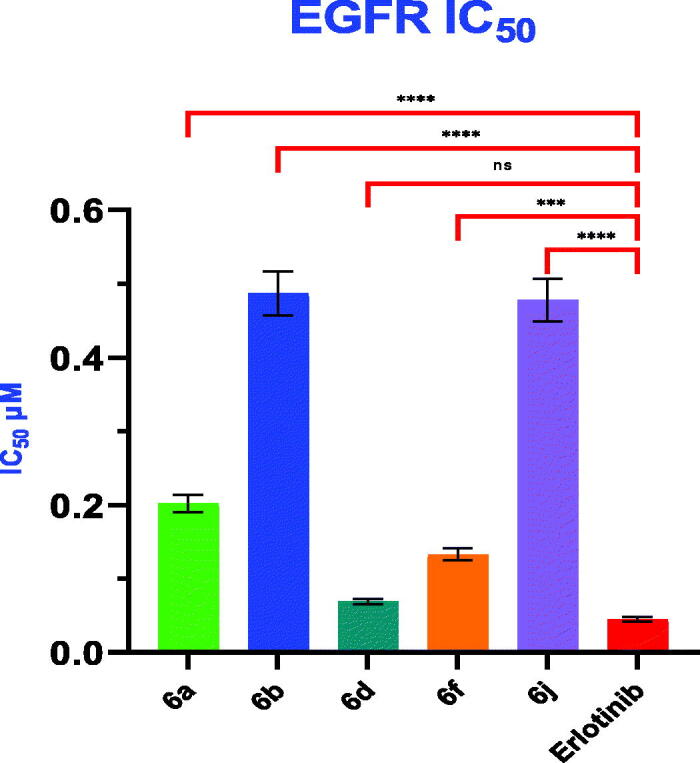
Graphical illustration for IC_50_ inhibition of EGFR of compounds **6a, 6b, 6d, 6f** and **6j** compared to erlotinib. Statistical significance was analysed by one-way ANOVA and Tukey’s multiple comparisons test (ns: non-significant, ***p < 0.001, ****p < 0.0001).

The most potent compounds **6a, 6b, 6d, 6f** and **6j**, with promising antiproliferative activity with mean inhibition range of 34.19–80.23%, were assayed for their EGFR inhibition. The results were summarised in Table 3 and Figure 5 as 50% inhibition concentration value (IC_50_) calculated from the concentration inhibition response curve. In this assay, erlotinib is used as positive control. Compound **6d** potently inhibited EGFR with IC_50_ = 0.069 **±** 0.004 µM in comparison to erlotinib with IC_50_ value of 0.045 **±** 0.003 µM. It was obvious that there was non-significant difference between IC_50_ values scored by both compound **6d** and erlotinib. Compound **6f** with IC_50_ = 0.133 **±** 0.008 µM, showed nearly half the potency of EGFR inhibition compared to compound **6d**. Moreover, compound **6b** was nearly equipotent in EGFR inhibition with compound **6j** with IC_50_ values of 0.487 **±** 0.030 and 0.478 **±** 0.029 µM, respectively. Compound **6d**, showed nearly three times the potency of EGFR inhibition compared to compound **6a** with IC_50_ = 0.202 **±** 0.012 µM. The antiproliferative activity of the compounds correlated with their high ability to inhibit EGFR. These biological results indicate that compound **6d** is a promising antiproliferative agent with EGFR inhibitory activity.

**Table 3.** EGFR kinase inhibitory activity of compounds **6a, 6b, 6d, 6f** and **6j** compared with erlotinib

Compound	EGFR IC_50_ (µM ± SD)^a^
**6a**	0.202 **±** 0.012
**6b**	0.487 **±** 0.030
**6d**	0.069 **±** 0.004
**6f**	0.133 **±** 0.008
**6j**	0.478 **±** 0.029
**Erlotinib**	0.045 **±** 0.003

^a^IC_50_ are presented as mean of three independent experiments.

#### Cell cycle analysis

**Figure 6.**
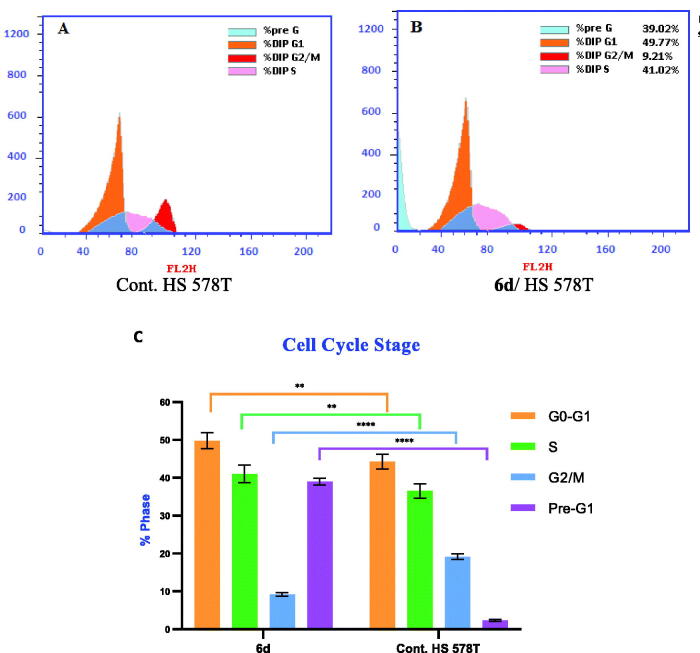
Cell cycle analysis in breast HS 578T cell line. (A) Control HS 578T; (B) Treatment with **6d**; (C) Graphical diagram for distribution of cell cycle analysis in treated and untreated control cells. Statistical significance was analysed by two-way ANOVA and Tukey’s multiple comparisons test (**p < 0.01, ****p < 0.0001).

Most cytotoxic drugs exert their antiproliferative activity by disturbing some checkpoints in the cell cycle. These checkpoints are distinct stages in the cell cycle whose disruption causes growth termination^46^. The cell cycle distribution was measured by DNA flow cytometric analysis, upon incubation of HS578T cells treated with compound **6d** for 24 h at its IC_50_ concentration (2.17 µM). The effect of compound **6d** on the cell population in different cell phases was recorded and presented in Figure 6. The results were compared to the cell cycle analysis of breast cancer HS 578 T cells as untreated control. The proportions of cells in both the G0-G1 phase and the S phase were significantly increased by 1.12-fold. Moreover, significant decrease in the cell population occurred at the G2/M phase with 52%. In comparison to control, the cell population in the pre-G1 phase significantly increased by 16.74-fold. These results showed that, compound **6d** caused induction of cell cycle arrest in breast cancer HS 578 T cells at the G1/S phase.

### Apoptosis assay

**Figure 7.**
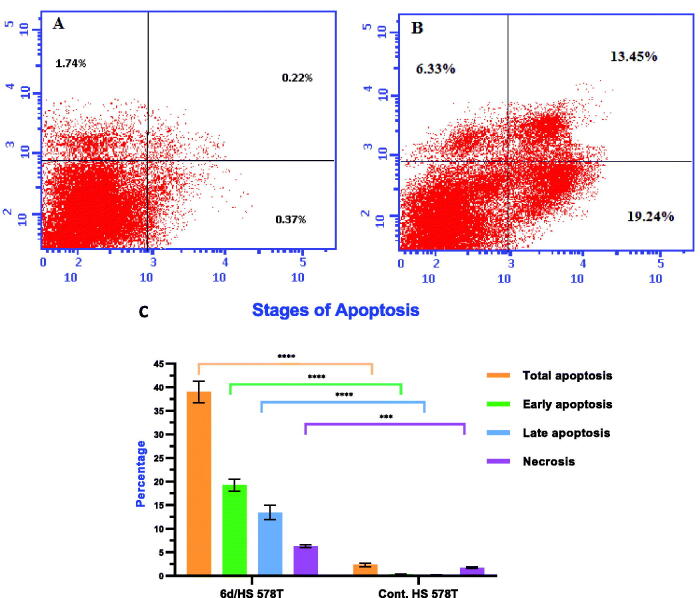
Effect of compound **6d** on Annexin V positive staining percentage in breast cancer HS 578T cells. (A) Control HS 578T; (B) Cells treated with **6d**; (C) Graphical diagram for percentage of apoptotic and necrotic cells in treated cells and untreated control cells. Statistical significance was analysed by two-way ANOVA and Tukey’s multiple comparisons test (***p < 0.001, ****p < 0.0001).

Annexin-V flow cytometry assay is an effective technique to determine apoptosis and for discrimination of programmed apoptosis and non-specific necrosis^26^^,^^47^. Annexin V is a protein with high affinity for phosphatidylserine (PS), a cell membrane component that translocate from the plasma membrane to the surface of the cell during apoptosis. Annexin-V and propidium iodide (PI) dual staining allows for the differentiation of live cells, early/late apoptotic cells, and necrotic cells. Fluorescent Annexin V conjugate can be used to identify PS on the cell surface. PI only enters dead cells and stains DNA^48^. The effect of compound **6d** on cell apoptosis was examined in breast cancer HS 578T cell line using a dual staining assay. The percentage of apoptotic cells increased significantly in early (from 0.37% to 19.24%) and late (from 0.22% to 13.45%) apoptotic phases (Table 4 and Figure 7). Compound **6d** increased total apoptosis significantly by 16.74-fold compared to the control.

**Table 4.** Annexin V/PI dual staining assay in breast HS 578T cells; Distribution of apoptotic cells after treatment with compound **6d**.

Comp.	Apoptosis			Necrosis
	Total	Early	Late	
6d	39.02	19.24	13.45	6.33
Control	2.33	0.37	0.22	1.74

#### Evaluation of caspase-3 activation

**Figure 8.**
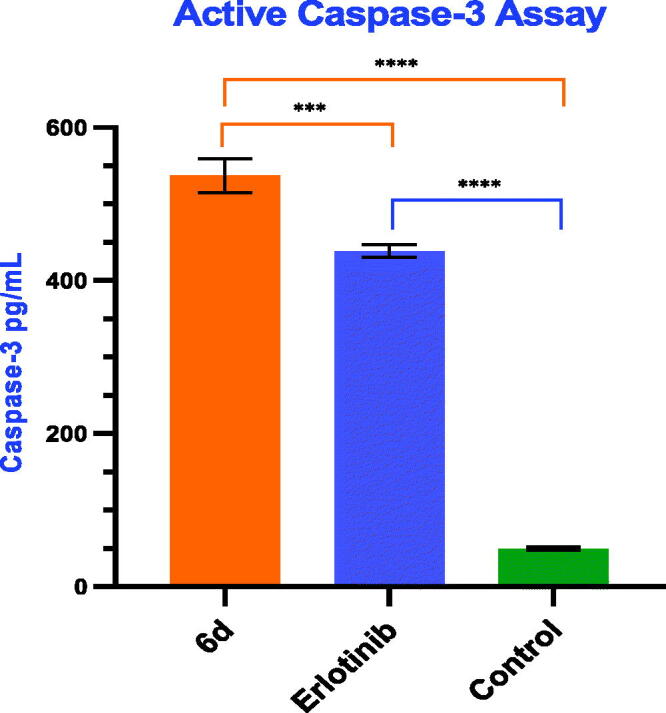
Caspase-3 activity in breast HS 578T cells: Effect of compound **6d** compared to erlotinib. Statistical significance was analysed by one-way ANOVA (***p < 0.001, ****p < 0.0001).

Apoptosis is triggered by the activation of caspases, particularly caspase-3, which is an initiator caspase responsible for apoptosis^49^. Therefore, the induction of apoptosis by compound **6d** was examined by the evaluation of caspase-3 activation in breast HS 578T cancer cell line. The effect of erlotinib as well as untreated cell line was used as positive and negative control, respectively. Compound **6d** and erlotinib significantly increased caspase-3 level by 10.75- and 8.78-fold, respectively when compared to negative control Figure 8. These results revealed that compound **6d** might induce apoptosis *via* a caspase-dependent mechanism.

### Molecular docking study

**Figure 9.**
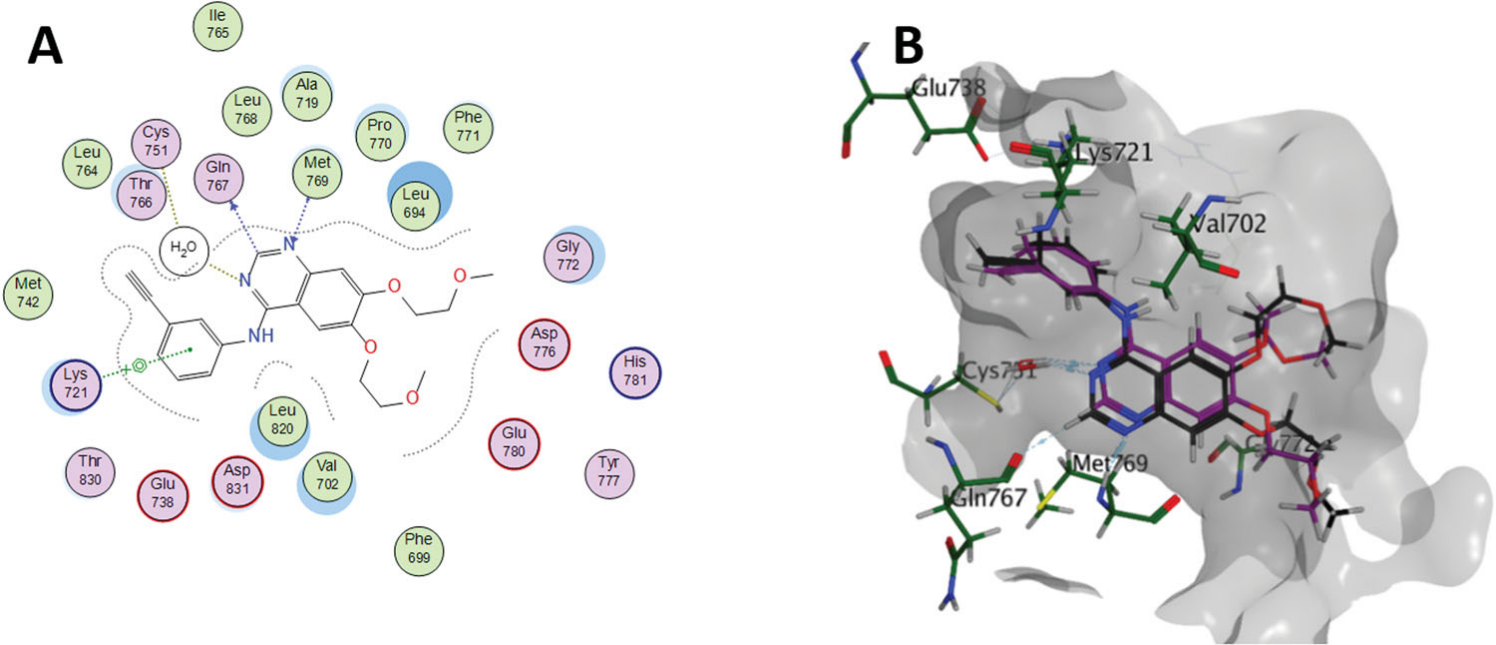
(A) 2D diagram of erlotinib interactions with EGFR binding pocket; (B) 3D overlay of the co-crystallised erlotinib (black) inhibitor and its re-docked pose (magenta) in the binding site of EGFR-TK showing minimum deviation of the docked pose from the co-crystallised one.

**Figure 10.**
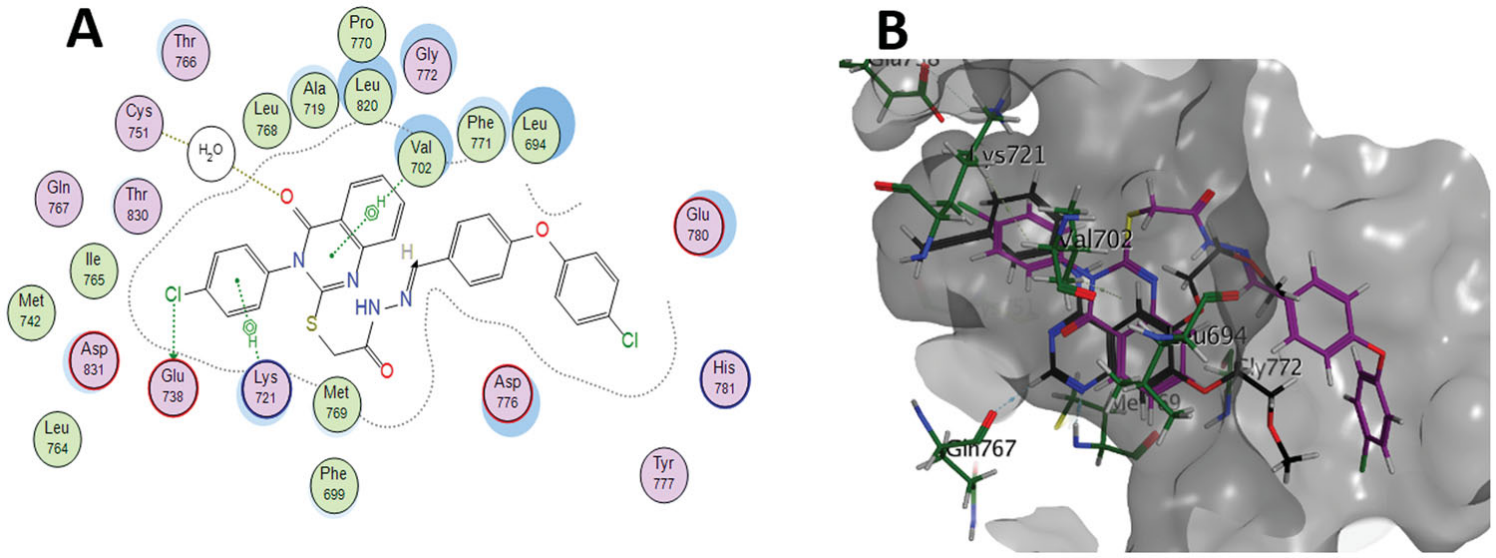
(A) 2D diagram and (B) 3D representation of molecular docking of compound **6d** (magenta) in the binding site of EGFR (PDB: 1M17).

Docking study was carried out for the most potent synthesised compounds into the EGFR protein complexed with erlotinib (PDB: 1M17)^50^ using MOE (2022.09) software^51^. The aim of this study was to investigate the interactions between the EGFR-TK and the highly active derivatives, **6a, 6b, 6d, 6f** and **6j**. The study began by redocking the co‐crystallised ligand, erlotinib, starting from a two‐dimensional structure and using the same protocol for preparation and analysis. The most active derivatives were then docked, and the resulted scores and the binding interactions are included along with the Lipinski parameters in Table 5. Erlotinib fits into the gorge of the active site of EGFR-TK and binds to Cys751 via a water bridge, the N1 atom of the erlotinib accepts a H-bond from the Met769 amide nitrogen. Erlotinib C-2 hydrogen is in close proximity and interacts with Gln767. The anilino ring is coplanar with the quinazoline ring and shows arene-cation interaction with Lys721 (Figure 9). Compounds **6a, 6b, 6d, 6f** and **6j** parameters were aligned with the Lipinski parameters, except for the logP parameter. Generally, compounds **6a, 6b, 6d, 6f** and **6j** were able to overlay as erlotinib with the quinazolinone ring inhabiting the same space of the quinazoline ring of erlotinib. The docking simulation showed that the least active compound **6b** is less binding to the active site with only 2 interactions: [HB (Cys751) and Arene-H (Val702)], while erlotinib has 4 interactions: [HB (Cys751), HB (Gln767), HB (Met769) and Arene-cation (Lys721)]. On the other hand, the docking of the most active compound **6d** (Figure 10) showed a comparable binding profile with the active site as the co-crystalized drug (erlotinib). Compound **6d** forms hydrogen bond with Cys751 via the water bridge and H-π interactions with Val702 which help stabilising the quinazolinone ring conformation. The *p*-chloro phenyl was able to overlay with the anilino ring of erlotinib and also interacts by H-π stacking with Lys721. The chlorine atom forms halogen bond^52^ with the carboxylate group of Glu738 which might be the rationale behind the higher *in vitro* EGFR enzymatic inhibition of **6d** when compared to its dechlorinated congener **6a**. (Docking poses of compounds **6a, 6b**, **6f** and **6j** are included in supplementary data)

**Table 5.** The docking score and Lipinski parameters of compounds **6a, 6b, 6d, 6f** and **6j.**

Compound number	Docking score (PDB: 1M17) (kcal/mol)	HBA (≤ 10)	HBD (≤ 5)	LogP (−4.0–5)	PSA (0–150) Å^2^	Interactions
**6a**	−8.27	5	1	6.66	83.4	HB (Cys751) Arene-H (Val702)
**6b**	−8.37	5	1	6.77	83.4	HB (Cys751) Arene-H (Val702)
**6d**	−8.07	5	1	7.32	83.4	HB (Cys751) Arene-H (Val702) Arene-H (Lys721) Halogen bond (Glu738)
**6f**	−7.55	6	1	6.67	92.6	HB (Lys721) Arene-H (Val702) Arene-H (Leu694) Halogen bond (Glu738)
**6j**	−7.98	5	1	5.86	77.4	HB (Cys751) Arene-H (Val702) Arene-H (Lys721) Halogen bond (Glu738)
**Erlotinib**	−8.68	6	1	2.26	74.7	HB (Cys751) HB (Gln767) HB (Met769) Arene-cation (Lys721)

## Conclusion

Nineteen quinazolin-4-one derivatives were synthesised to mimic the third generation EGFR TKIs. The antiproliferative activity were assessed for all the prepared compounds. The results showed the significance of the thioacetyl linker for the antiproliferative activity. The compounds showed promising results with **6d** exhibiting high inhibition of EGFR with IC_50_ = 0.069 ± 0.004 µM which is comparable with the positive control. In addition, **6d** demonstrated an excellent *in vitro* antiproliferative activity with mean growth inhibition of 80.23% and was chosen by the National Cancer Institute (NCI), Maryland, USA for further investigation in which compound **6d** exhibited potent and broad-spectrum antiproliferative activity against most of the tested cancer cell lines, with GI_50_ values in the range of 1.29–5.97 µM. DNA-flow cytometric analysis demonstrated the inhibition of cell proliferation by compound **6d** at G1/S phase. Docking study showed that compound **6d** interacts similarly to erlotinib and highlights the role of the halogen bond in enhancing the binding to the EGFR binding site for further developments.

## Supplementary Material

Supplemental Material
