# Supplementary material for: Design and synthesis of novel quinazolinone-based derivatives as EGFR inhibitors with antitumor activity
Source: J Enzyme Inhib Med Chem. 2022 Sep 22;37(1):2644–59. doi: 10.1080/14756366.2022.2118735 (PMC9518264; doi:10.1080/14756366.2022.2118735)

Supplementary data

Design and synthesis of novel quinazolinone-based derivatives as EGFR inhibitors with potential antitumor activity

Amr Sonousi[1,2,*], Rasha A. Hassan[1], Eman O. Osman[1], Amr M. Abdou[3] and Soha H. Emam[1]

1. Pharmaceutical Organic Chemistry Department, Faculty of Pharmacy, Cairo University, Cairo 11562, Egypt
2. University of Hertfordshire hosted by Global Academic Foundation, New Administrative Capital, Cairo, Egypt
3. Department of Microbiology and Immunology, National Research Centre, Dokki, Giza, 12622, Egypt

Correspondence to: Amr Sonousi,

Department of Pharmaceutical Organic Chemistry, Faculty of Pharmacy, Cairo University, Cairo, 11562, Egypt.

Address: 33 Kasr El-Aini Street, Cairo, Egypt.

Tel: 002023639307.   Fax: 002023635140.

University of Hertfordshire hosted by Global Academic Foundation, New Administrative Capital, Cairo, Egypt

E-mail: amr.motawi@pharma.cu.edu.eg

This PDF file includes the biological evaluation of anticancer activity by NCI against a panel of 60 cell lines, molecular docking into the active sites of EGFR enzyme, and spectral data (^{1}H NMR and ^{13}C NMR) of the newly synthesized quinazolinone derivatives.

Supplementary data

(Biological evaluation - Molecular docking validation - Chemistry)

1- Experimental

1.1 Biological evaluation

1.1.1. Measurement of anticancer activity against a panel of 60 cell lines.

Anticancer activity screening of the newly synthesized compounds was measured *in vitro* utilizing 60 different human cancer cell lines provided by US NCI according to previously reported standard procedure as follows:

- Cells were seeded into 96-well microtiter plates in a density of 5,000-1,000 cells per 100 µL/well. Cells were then incubated at 37°C, 5% CO_2, 95% air, and 100% relative humidity for 24 h before addition of experimental compounds. After 24 hr, two plates of each cell line were fixed in situ with trichloroacetic acid (TCA), to present a measurement of the cell population for each cell line at the time of compound exposure (Tz).
- Experimental compounds were solubilized in DMSO at 400-fold, the desired final maximum test concentration, and stored frozen before use. At the time of compound addition, an aliquot of frozen concentrate was thawed and diluted to twice the desired final maximum test concentration with complete medium containing 50 µg/mL gentamicin.
- Additional four, 10-fold or ½ log serial dilutions were made to provide a total of five compound concentrations plus control. Aliquots of 100 µL of these different compound dilutions were added to the appropriate microtiter wells containing 100 µL of the medium, resulting in the required final compound concentrations.
- Following compound addition, the plates were incubated for an additional 48 hr at 37°C, 5% CO_2, 95% air, and 100% relative humidity. For adherent cells, the assay is terminated by the addition of cold TCA. Cells were fixed in situ by the gentle addition of 50 µL of cold 50% (w/v) TCA (final concentration, 10% TCA and incubated for 60 min at 4°C. The supernatant was discarded, and the plates were washed five times with tap water and air-dried.
- Sulforhodamine B (SRB) solution (100 µL) at 0.4% (w/v) in 1% acetic acid was added to each well and plates were incubated for 10 min at room temperature. After staining, the unbound dye was removed by washing five times with 1% acetic acid and the plates were air-dried. The bound stain was subsequently solubilized with 10 mM Trizma base, and the absorbance was obtained using an automated plate reader at a wave-length of 515 nm. For suspension cells, we used the same methodology except that the assay was terminated by fixing settled cells at the bottom of the wells by gently adding 50 µL of 80% TCA (final concentration, 16% TCA). Using the seven absorbance measurements [time zero (Tz), control growth (C), and test growth in the presence of a compound at the five concentration levels (Ti)], the percentage growth was calculated at each of the compound concentrations levels. Percentage growth inhibition is calculated as follows:

$$[(Ti - Tz)/(C - Tz)] \times 100 \text{ for concentrations for which } Ti \geq Tz,$$

$$[(Ti - Tz)/Tz] \times 100 \text{ for concentrations for which } Ti > Tz$$

For each experimental agent. Growth inhibition of 50% (GI_{50}) is calculated from $[(Ti - Tz)/(C - Tz)] \times 100 = 50$, which is the compound concentration resulting in a 50% reduction in the net protein increase (as measured by SRB staining) in control cells during the compound incubation; however, if the effect is not reached or is exceeded, the value for that parameter is expressed as greater or less than the maximum or minimum concentration tested.

In vitro growth inhibition % (GI %) of the synthesized compounds **3a-g** against a panel of 60 tumor cell lines at 10 μM.

	**3a**	**3b**	**3c**	**3d**	**3e**	**3f**	**3g**
**Subpanel**							
**Leukemia**							
K-562	--	--	24.8	--	--	--	--
SR	--	--	16.0	--	--	--	--
**NSC lung cancer**							--
HOP-92	--		--	--	--	--	16.01
NCI-H226	--		15.23	--	--	--	--
**Colon cancer**							
No inhibition on all colon cell lines							
**CNS cancer**							
No inhibition on all CNS cancer cell lines							
**Melanoma**							
No inhibition on all melanoma cell lines							
**Ovarian cancer**							
No inhibition on all ovarian cancer cell lines							
**Renal cancer**							
RXF 393	--		--	NT	--	--	25.11
UO-31	--	--	21.64	--	--	--	20.99
**Prostate cancer**							
No inhibition on all prostate cancer cell lines							
**Breast cancer**							
No inhibition on all breast cancer cell lines							
**Mean inhibition**	**<0**	**<0**	1.6	**<0**	**<0**	**<0**	**<0**

-- Growth inhibition % produced by the compound is below 15%.

1.1.2. *In vitro* cell based EGFR inhibitory assay

In vitro EGFR inhibitory activities of compounds **6a, 6b, 6d, 6f, 6j,** doxorubicin and erlotinib were evaluated using serial dilutions (1.0, 0.1, 0.01, 0.001 µM) against EGFR (KDR) Kinase Assay Kit Catalog # 40325 according to manufacturer's instructions. In brief, the master mixture was produced (25 µL per well) and poured into each well. Each well received 5 µL of inhibitor solution designated as "Test Inhibitor". The "Positive Control" and "Blank" groups received 5 µL of the same solution without the inhibitor (Inhibitor buffer). In order to prepare 3 mL of kinase buffer, 600 µL of kinase buffer were combined with 2400 µL of water. The blank wells received 20 µL of kinase buffer. The amount of EGFR required for the test was measured and the enzyme was diluted to 1 ng/ µL with kinase buffer. 20 µL of diluted EGFR enzyme was added to the wells designated as "Test Inhibitor Control" and "Positive Control" to start the reaction and the mixtures were incubated at 30 °C for 45 minutes. After the 45 minutes, each well received 50 µL of Kinase-Glo Max reagent and the plate was incubated at room temperature for 15 minutes. The luminescence was measured with a microplate reader.

1.1.3. Cell cycle analysis

Flow cytometry was used to analyse the cell cycle using ab139418 propidium iodide flow cytometry kit/BD (Abcam, Cambridge, UK), as directed by the manufacturer guidelines. HS 578T cells were treated with compound **6d** at its IC_{50} concentrations (2.17 µM) for 24 h. The cells were washed twice with ice-cold phosphate buffer saline (PBS) and collected by centrifugation. The cells were then fixed using ice-cold 66% (v/v) ethanol, washed with PBS, and re-suspended with 0.1 mg/mL RNase to digest cellular RNA and thus minimize stained RNA in the background. The cells were next stained with PI, a fluorescent molecule that may bind to nucleic acid, at a concentration of 40 mg/mL. In cells, PI attaches to DNA in proportion to its amount. Because the DNA content of cells at different stages of the cell cycle differs, the fluorescence intensity can be used to assess the stage of cell growth. FacsCalibur (BD Biosciences, USA) was used to estimate cell fluorescence, which was then examined using Cell-Quest software (Becton Dickinson). Cell cycle analysis of HS 578T cells without any treatment was used as control.

1.1.4. Apoptosis assay

According to the manufacturer's instructions, flow cytometry was used to analyze apoptosis using Annexin V-FITC and propidium iodide double-staining apoptosis detection kit (Biovision, USA). Annexin V is a phosphatidylserine (PS) binding protein with a high affinity. After beginning apoptosis, the latter is a cell membrane component that translocate from the inner face of the plasma membrane to the cell surface. PS can be detected on the cell surface using a fluorescent Annexin V conjugate. HS 578T cells ($5x10^5$) were exposed to compound **6d** at its IC_{50} concentrations, and then subsequently incubated for 24 hours. After that, the cells were centrifuged and resuspended in 500 mL of binding buffer. Annexin V-FITC and PI double staining was accomplished by mixing 5 µL of Annexin V-FITC with 5 µL of PI. The cells were then

incubated for 15 minutes in the dark at room temperature. FacsCalibur was used to assess cell fluorescence after incubation (BD Biosciences, USA). The results were represented using dot-plot graphs.

1.1.5. Caspase-3 enzyme assay

The level of the apoptotic marker caspase-3 was measured using Invitrogen ELISA Kit Catalog # KHO1091. The procedure of the used kit was performed according to the manufacturer's instructions. Let all components to reach room temperature before use. Gently combine all liquid reagents prior to use. Add 100 µL of the standard diluent buffer to the zero standard wells. Add 100 µL of standards and controls or diluted samples to the appropriate microtiter wells. Incubate for 2 h at room temperature. Pipette 100 µL of Caspase-3 (Active) detection antibody solution into each well. Incubate for 1 h at room temperature. Add 100 µL anti-rabbit IgG HRP working solution to each well. Prepare the working dilution and incubate for 30 minutes at room temperature. Add 100 µL of stabilized chromogen to each well. The liquid in the wells will begin to turn blue. Incubate for 30 minutes at room temperature and in the dark. Add 100 µL of stop solution to each well. The solution in the wells should change from blue to yellow. Read the absorbance of each well at 450 nm. Read the plate within 2 h after adding the stop solution. Use a curve fitting software to generate the standard curve. Read the concentrations for unknown samples and controls from the standard curve.

2- Molecular modeling

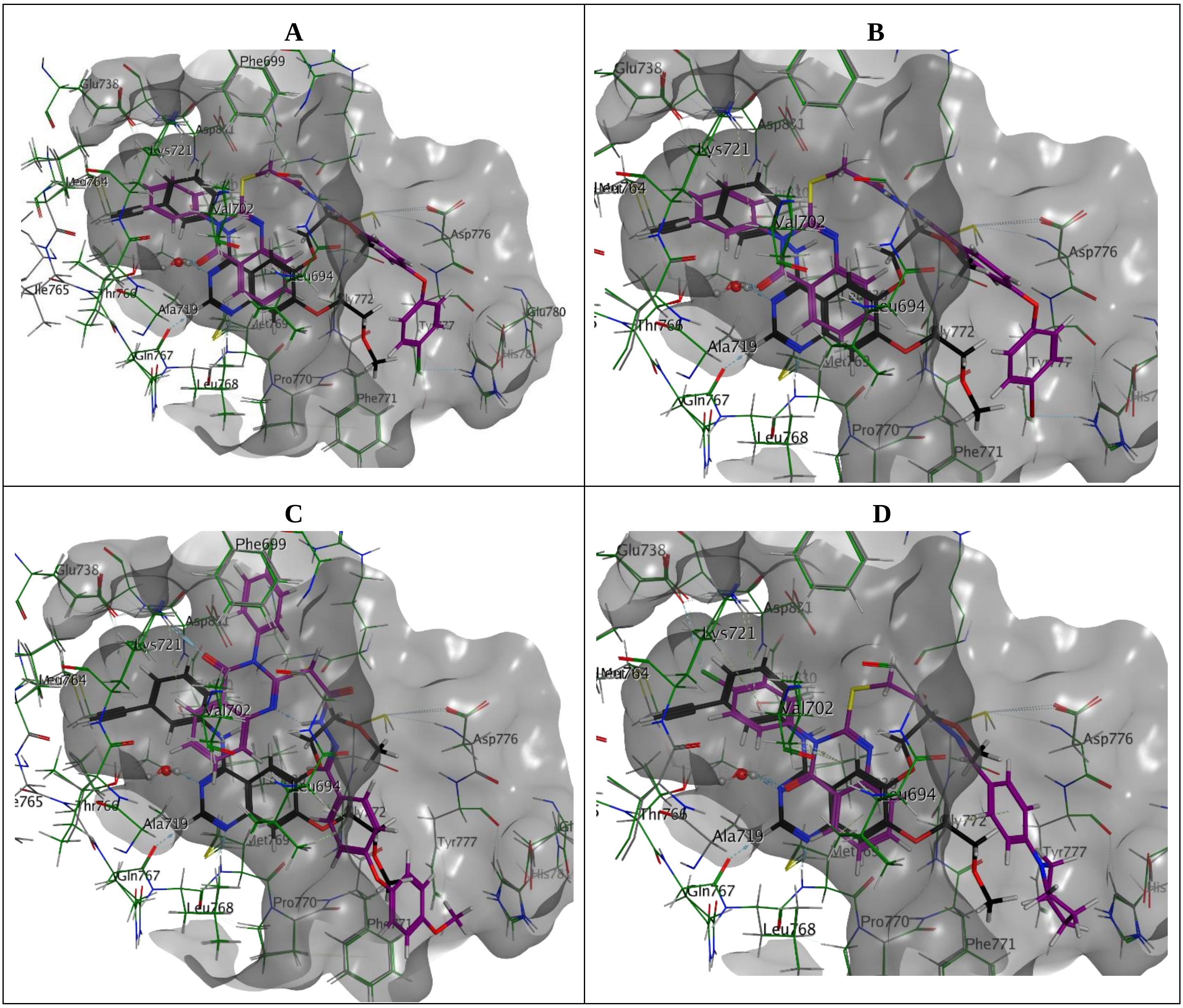

Figure 1. 3D representation of molecular docking of compounds A) **6a**; B) **6b**; C) **6f**; and D) **6j** in the binding site of EGFR (PDB: 1M17).

3- ^{1}H NMR and [13] C NMR spectra of the new derivatives.

Compound 3a

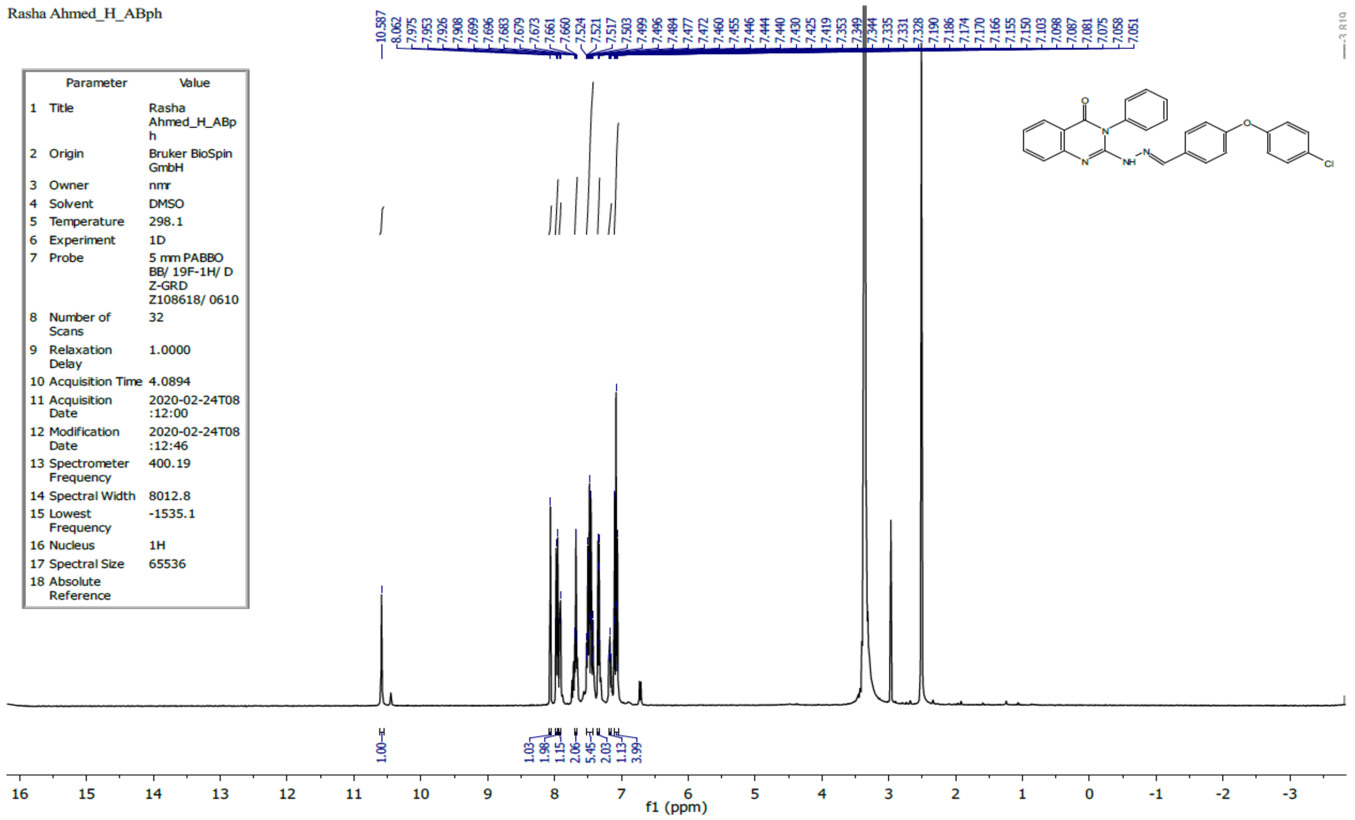

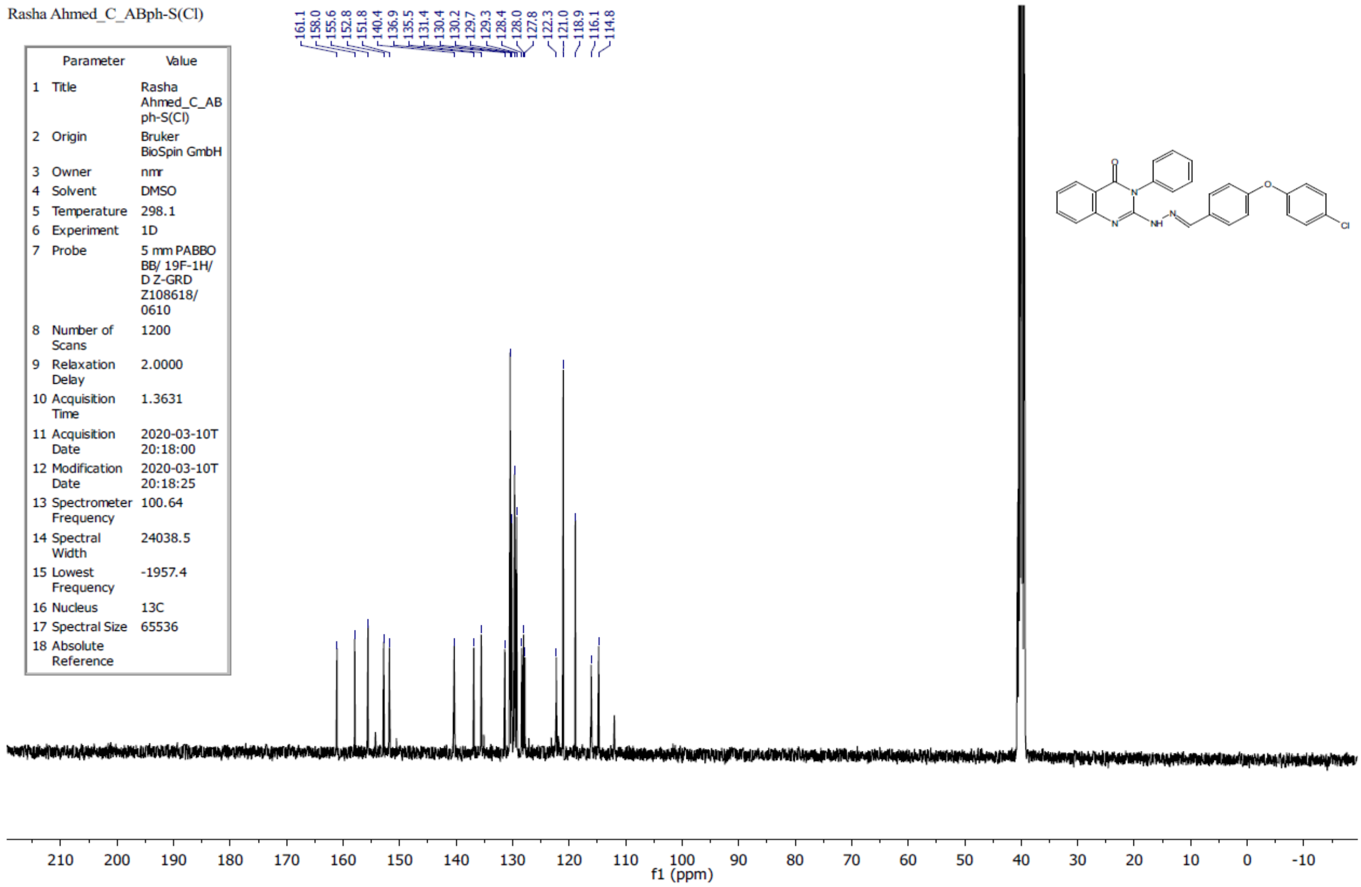

Compound 3b

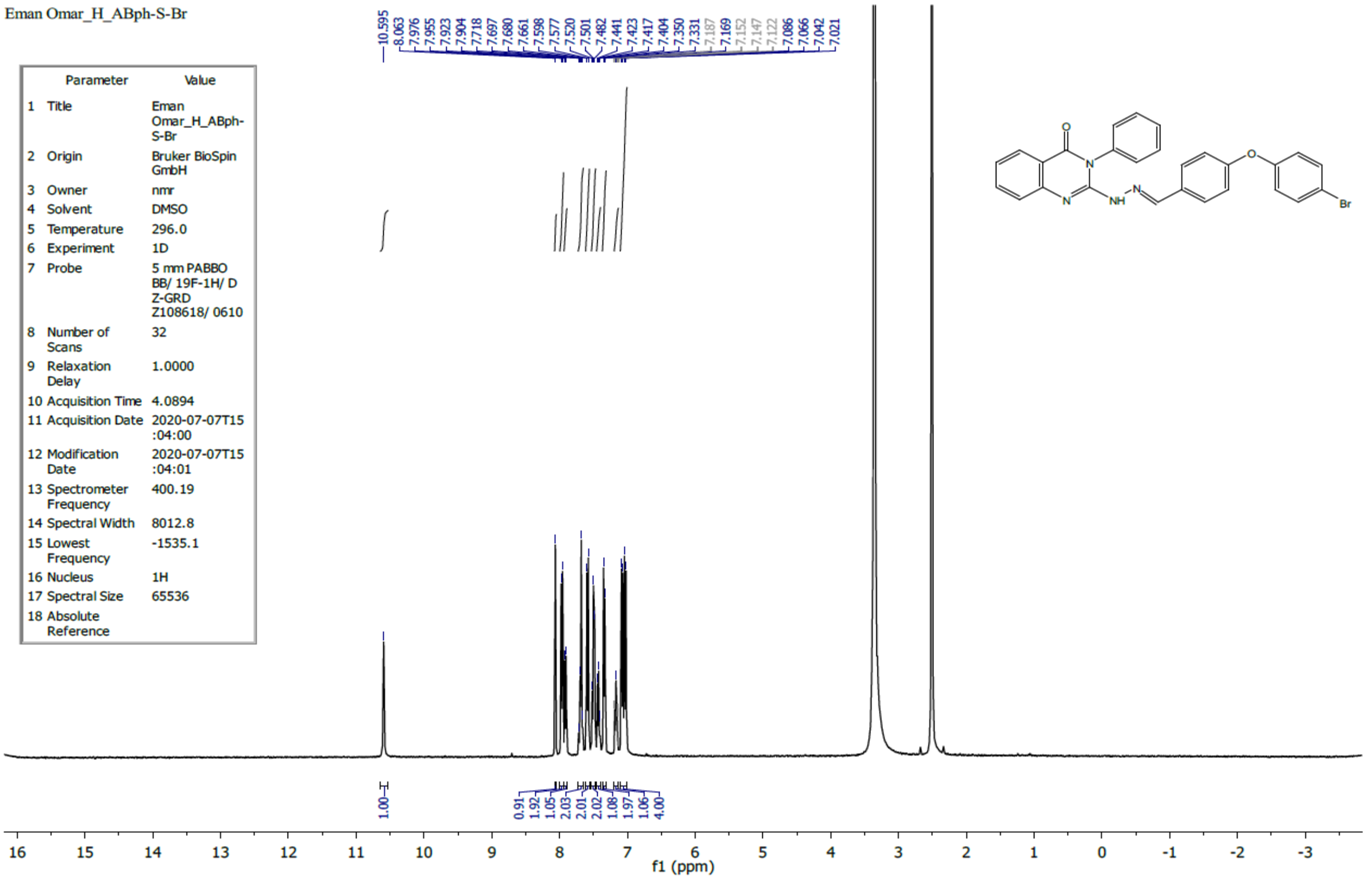

Eman Omar_H_ABph-S-Br
Parameter | Value
1 Title | Eman Omar_H_ABph-S-Br
2 Origin | Bruker BioSpin GmbH
3 Owner | nmr
4 Solvent | DMSO
5 Temperature | 296.0
6 Experiment | 1D
7 Probe | 5 mm PABBO BB/ 19F-1H/ D Z-GRD Z108618/ 0610
8 Number of Scans | 32
9 Relaxation Delay | 1.0000
10 Acquisition Time | 4.0894
11 Acquisition Date | 2020-07-07T15:04:00
12 Modification Date | 2020-07-07T15:04:01
13 Spectrometer Frequency | 400.19
14 Spectral Width | 8012.8
15 Lowest Frequency | -1535.1
16 Nucleus | 1H
17 Spectral Size | 65536
18 Absolute Reference
10.595
8.063 7.976 7.955 7.923 7.904 7.718 7.697 7.680 7.661 7.598 7.577 7.520 7.501 7.482 7.441 7.423 7.417 7.404 7.350 7.331 7.187 7.169 7.152 7.147 7.122 7.086 7.066 7.042 7.021
1.00
0.91 1.92 1.05 2.03 2.01 2.02 1.08 1.97 1.06 4.00
O
N
N
NH
N
O
Br
f1 (ppm)

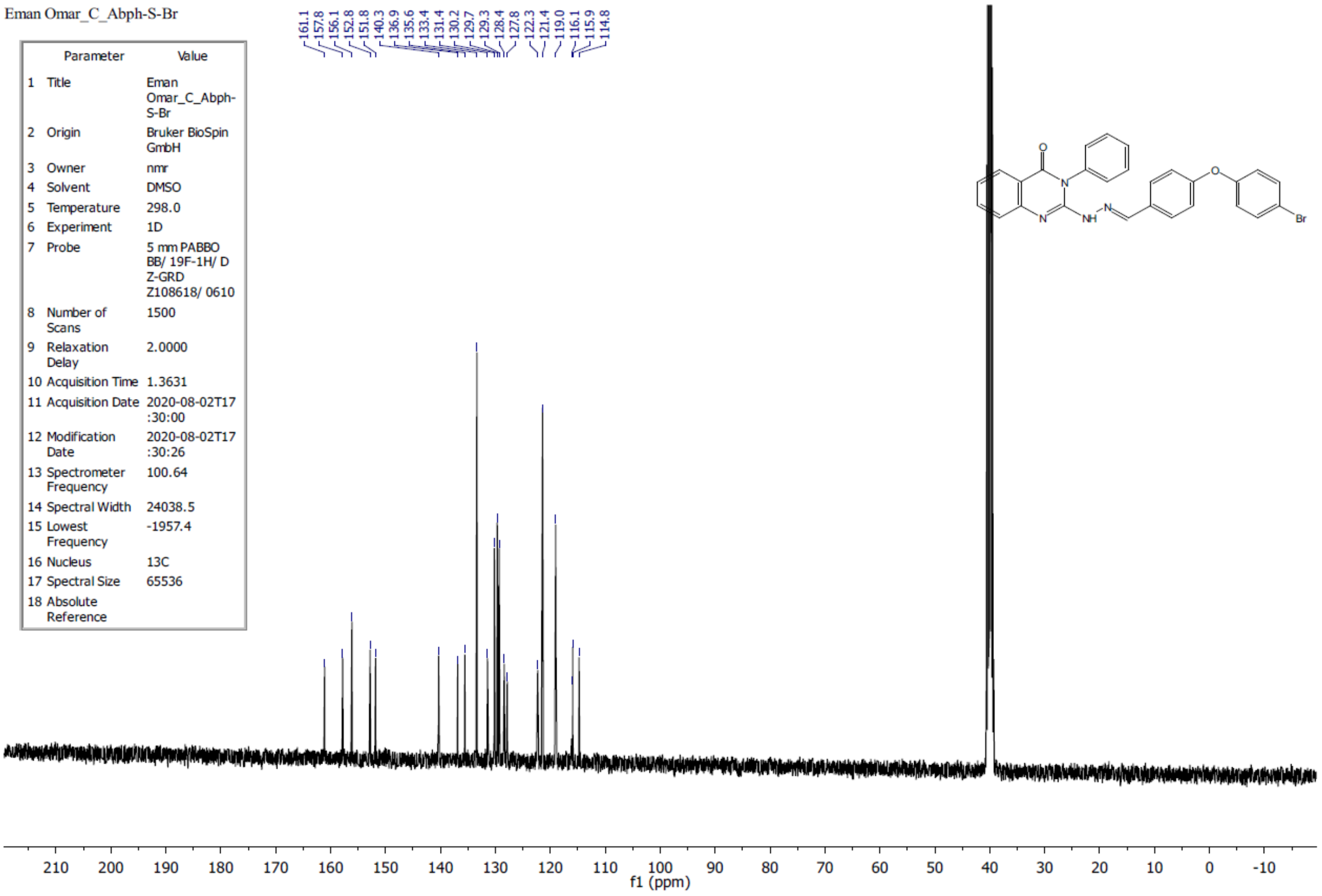

Eman Omar_C_Abph-S-Br
Parameter | Value
1 Title | Eman Omar_C_Abph-S-Br
2 Origin | Bruker BioSpin GmbH
3 Owner | nmr
4 Solvent | DMSO
5 Temperature | 298.0
6 Experiment | 1D
7 Probe | 5 mm PABBO BB/ 19F-1H/ D Z-GRD Z108618/ 0610
8 Number of Scans | 1500
9 Relaxation Delay | 2.0000
10 Acquisition Time | 1.3631
11 Acquisition Date | 2020-08-02T17:30:00
12 Modification Date | 2020-08-02T17:30:26
13 Spectrometer Frequency | 100.64
14 Spectral Width | 24038.5
15 Lowest Frequency | -1957.4
16 Nucleus | 13C
17 Spectral Size | 65536
18 Absolute Reference
161.1 157.8 156.1 152.8 151.8 140.3 136.9 135.6 133.4 131.4 130.2 129.7 129.3 128.4 127.8 122.3 121.4 119.0 116.1 115.9 114.8
O
N
N
NH
N
O
Br
f1 (ppm)

Compound 3c

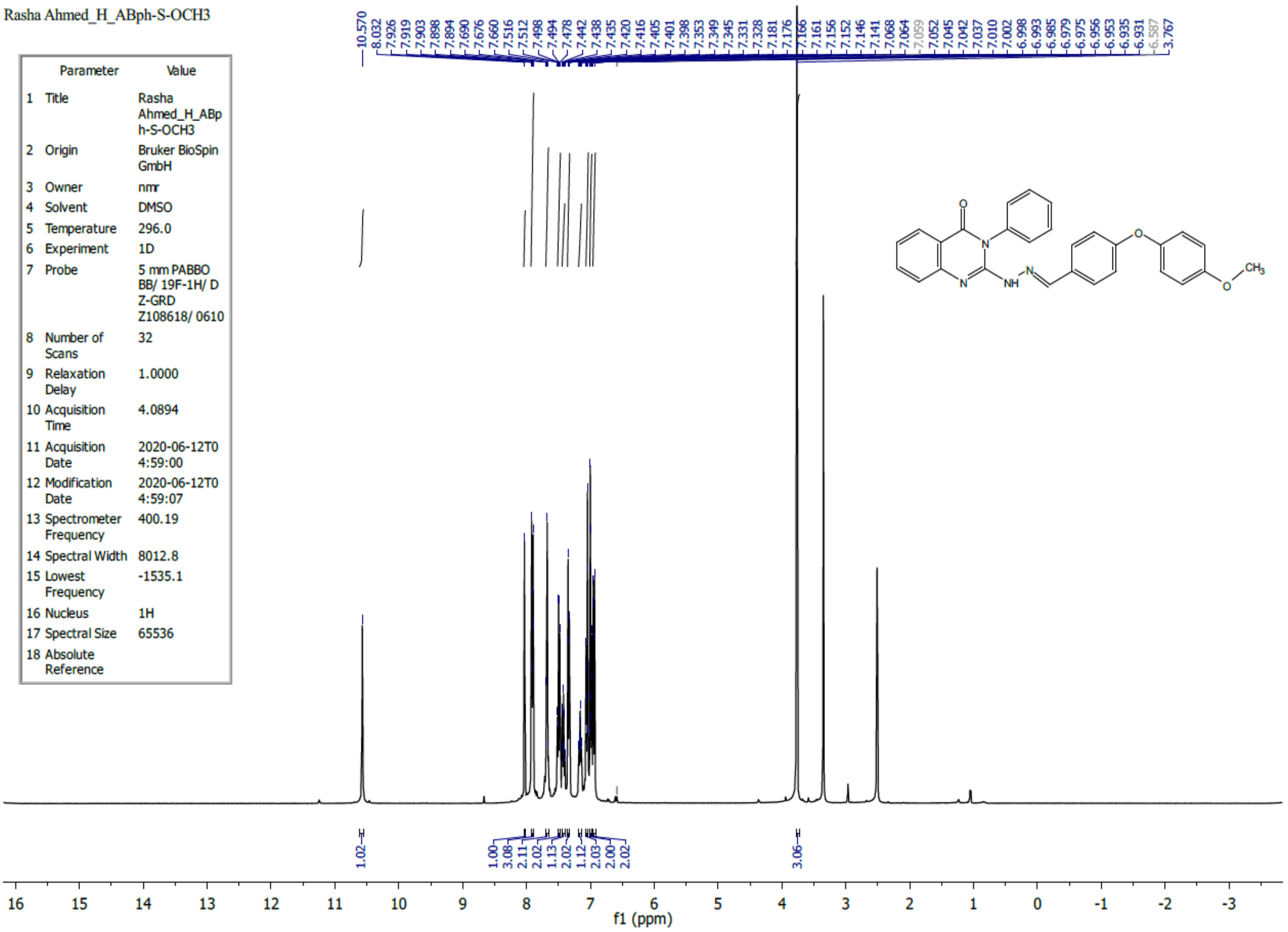
Rasha Ahmed_H_ABph-S-OCH3
Parameter | Value
1 Title | Rasha Ahmed_H_ABph-S-OCH3
2 Origin | Bruker BioSpin GmbH
3 Owner | nmr
4 Solvent | DMSO
5 Temperature | 296.0
6 Experiment | 1D
7 Probe | 5 mm PABBO BB/ 19F-1H/ D Z-GRD Z108618/ 0610
8 Number of Scans | 32
9 Relaxation Delay | 1.0000
10 Acquisition Time | 4.0894
11 Acquisition Date | 2020-06-12T04:59:00
12 Modification Date | 2020-06-12T04:59:07
13 Spectrometer Frequency | 400.19
14 Spectral Width | 8012.8
15 Lowest Frequency | -1535.1
16 Nucleus | 1H
17 Spectral Size | 65536
18 Absolute Reference
10.570 8.032 7.926 7.919 7.903 7.898 7.894 7.690 7.676 7.660 7.516 7.512 7.498 7.494 7.478 7.442 7.438 7.435 7.420 7.416 7.405 7.401 7.398 7.353 7.349 7.345 7.331 7.328 7.181 7.176 7.166 7.161 7.156 7.152 7.146 7.141 7.068 7.064 7.059 7.052 7.045 7.042 7.037 7.010 7.002 6.998 6.993 6.985 6.979 6.975 6.956 6.953 6.935 6.931 6.587 3.767
1.02 1.00 3.08 2.11 2.02 1.13 2.02 1.12 2.03 2.00 2.02 3.06
O N N NH N O O CH3
16 15 14 13 12 11 10 9 8 7 6 5 4 3 2 1 0 -1 -2 -3
f1 (ppm)

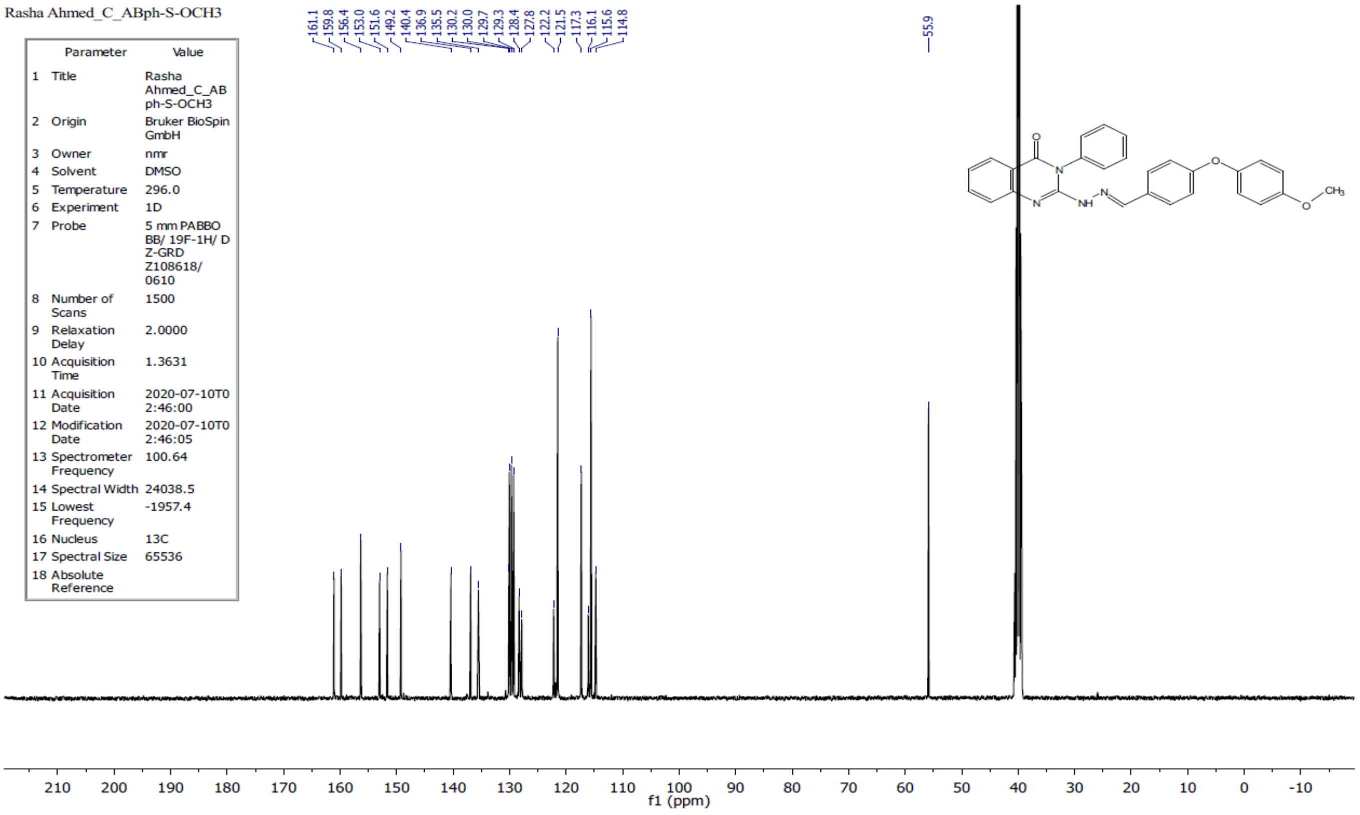
Rasha Ahmed_C_ABph-S-OCH3
Parameter | Value
1 Title | Rasha Ahmed_C_ABph-S-OCH3
2 Origin | Bruker BioSpin GmbH
3 Owner | nmr
4 Solvent | DMSO
5 Temperature | 296.0
6 Experiment | 1D
7 Probe | 5 mm PABBO BB/ 19F-1H/ D Z-GRD Z108618/ 0610
8 Number of Scans | 1500
9 Relaxation Delay | 2.0000
10 Acquisition Time | 1.3631
11 Acquisition Date | 2020-07-10T02:46:00
12 Modification Date | 2020-07-10T02:46:05
13 Spectrometer Frequency | 100.64
14 Spectral Width | 24038.5
15 Lowest Frequency | -1957.4
16 Nucleus | 13C
17 Spectral Size | 65536
18 Absolute Reference
161.1 159.8 156.4 153.0 151.6 149.2 140.4 136.9 135.5 130.2 130.0 129.7 129.3 128.4 127.8 122.2 121.5 117.3 116.1 115.6 114.8
55.9
O N N NH N O O CH3
210 200 190 180 170 160 150 140 130 120 110 100 90 80 70 60 50 40 30 20 10 0 -10
f1 (ppm)

Compound 3d

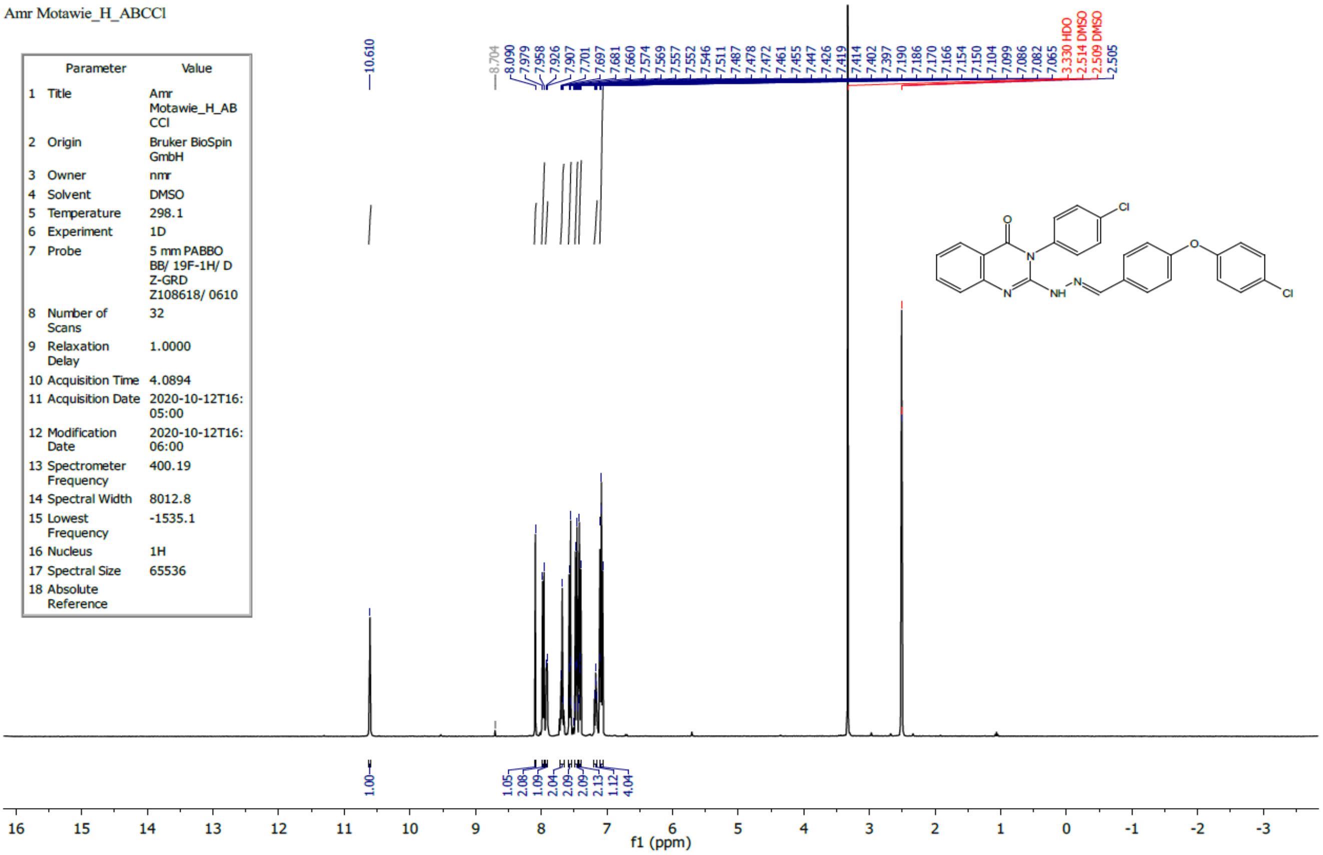
Amr Motawie_H_ABCCl
Parameter Value
1 Title Amr Motawie_H_ABCCl
2 Origin Bruker BioSpin GmbH
3 Owner nmr
4 Solvent DMSO
5 Temperature 298.1
6 Experiment 1D
7 Probe 5 mm PABBO BB/ 19F-1H/ D Z-GRD Z108618/ 0610
8 Number of Scans 32
9 Relaxation Delay 1.0000
10 Acquisition Time 4.0894
11 Acquisition Date 2020-10-12T16:05:00
12 Modification Date 2020-10-12T16:06:00
13 Spectrometer Frequency 400.19
14 Spectral Width 8012.8
15 Lowest Frequency -1535.1
16 Nucleus 1H
17 Spectral Size 65536
18 Absolute Reference
10.610
8.704
3.330 HDO
2.514 DMSO
2.509 DMSO
2.505
O
N
Cl
N
NH
N
O
Cl
f1 (ppm)

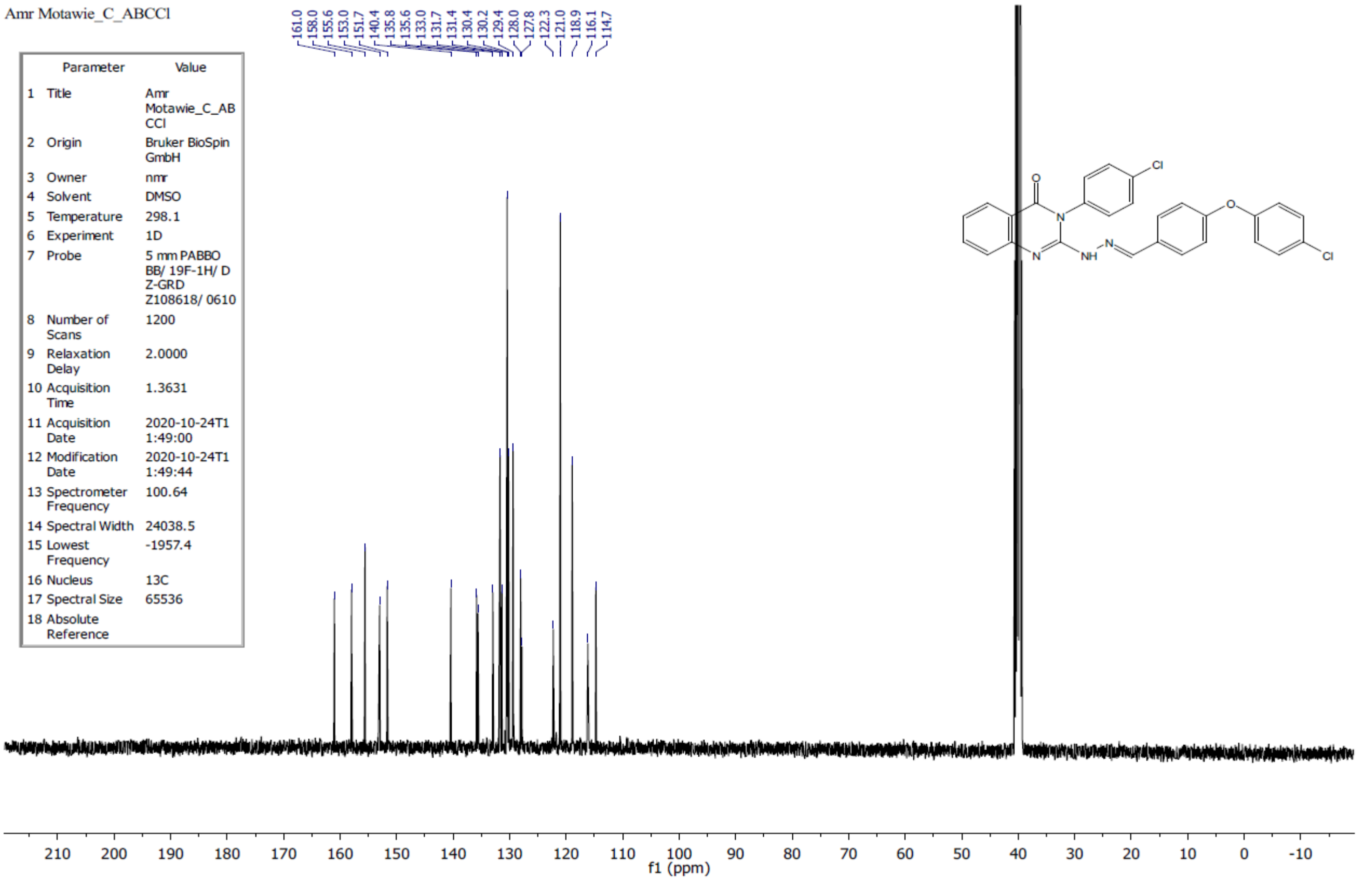
Amr Motawie_C_ABCCl
Parameter Value
1 Title Amr Motawie_C_ABCCl
2 Origin Bruker BioSpin GmbH
3 Owner nmr
4 Solvent DMSO
5 Temperature 298.1
6 Experiment 1D
7 Probe 5 mm PABBO BB/ 19F-1H/ D Z-GRD Z108618/ 0610
8 Number of Scans 1200
9 Relaxation Delay 2.0000
10 Acquisition Time 1.3631
11 Acquisition Date 2020-10-24T11:49:00
12 Modification Date 2020-10-24T11:49:44
13 Spectrometer Frequency 100.64
14 Spectral Width 24038.5
15 Lowest Frequency -1957.4
16 Nucleus 13C
17 Spectral Size 65536
18 Absolute Reference
161.0
158.0
155.6
153.0
151.7
140.4
135.8
135.6
133.0
131.7
131.4
130.4
130.2
129.4
128.0
127.8
122.3
121.0
118.9
116.1
114.7
O
N
Cl
N
NH
N
O
Cl
f1 (ppm)

Compound 3e

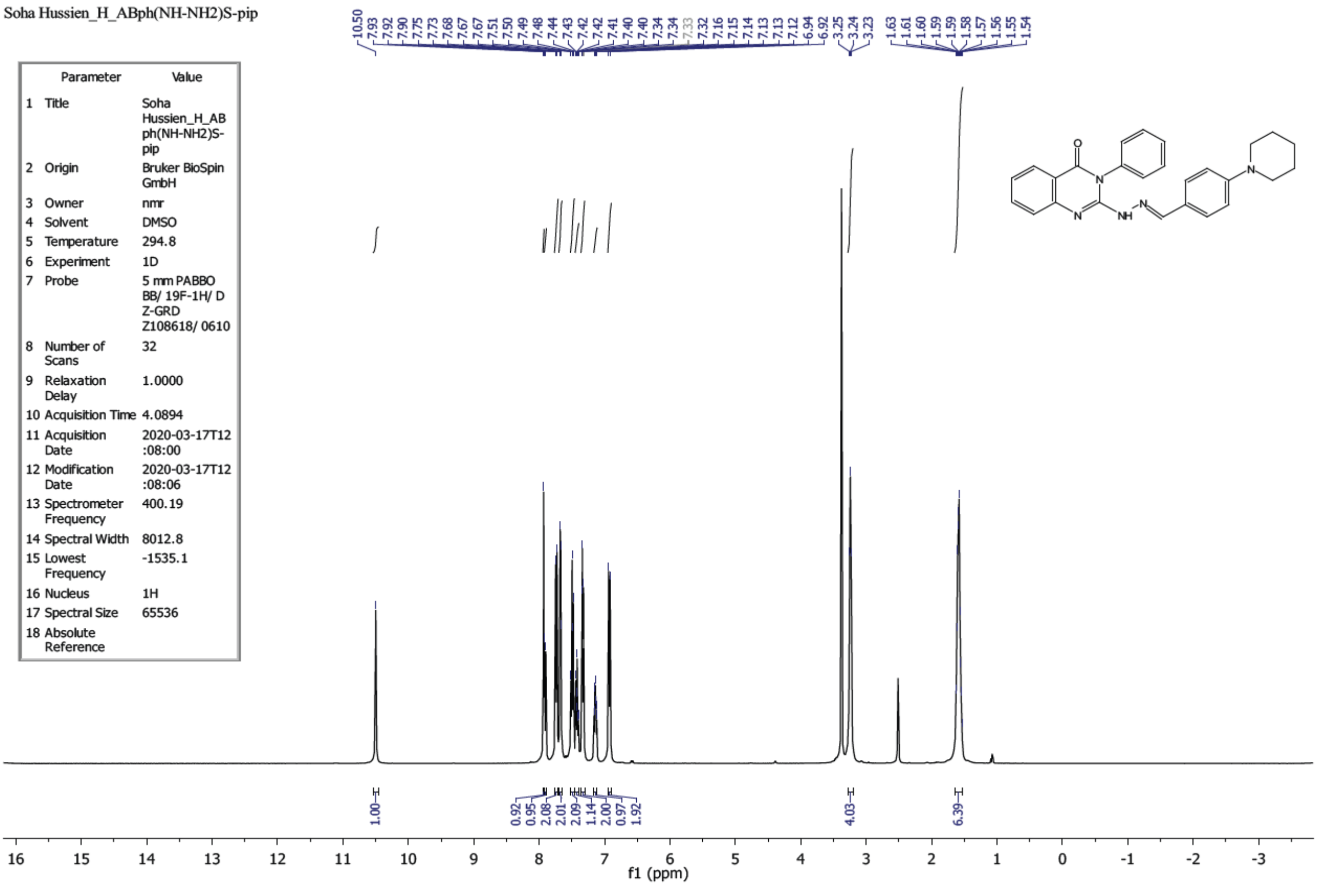
Soha Hussien_H_ABph(NH-NH2)S-pip
Parameter Value
1 Title Soha Hussien_H_ABph(NH-NH2)S-pip
2 Origin Bruker BioSpin GmbH
3 Owner nmr
4 Solvent DMSO
5 Temperature 294.8
6 Experiment 1D
7 Probe 5 mm PABBO BB/ 19F-1H/ D Z-GRD Z108618/ 0610
8 Number of Scans 32
9 Relaxation Delay 1.0000
10 Acquisition Time 4.0894
11 Acquisition Date 2020-03-17T12:08:00
12 Modification Date 2020-03-17T12:08:06
13 Spectrometer Frequency 400.19
14 Spectral Width 8012.8
15 Lowest Frequency -1535.1
16 Nucleus 1H
17 Spectral Size 65536
18 Absolute Reference
10.50 7.93 7.92 7.90 7.75 7.73 7.68 7.67 7.67 7.51 7.50 7.49 7.48 7.44 7.43 7.42 7.42 7.41 7.40 7.40 7.34 7.34 7.33 7.32 7.16 7.15 7.14 7.13 7.13 7.12 6.94 6.92 3.25 3.24 3.23 1.63 1.61 1.60 1.59 1.59 1.58 1.57 1.56 1.55 1.54
O N N NH N N
1.00 0.92 0.95 2.08 2.01 2.09 1.14 2.00 0.97 1.92 4.03 6.39
16 15 14 13 12 11 10 9 8 7 6 5 4 3 2 1 0 -1 -2 -3
f1 (ppm)

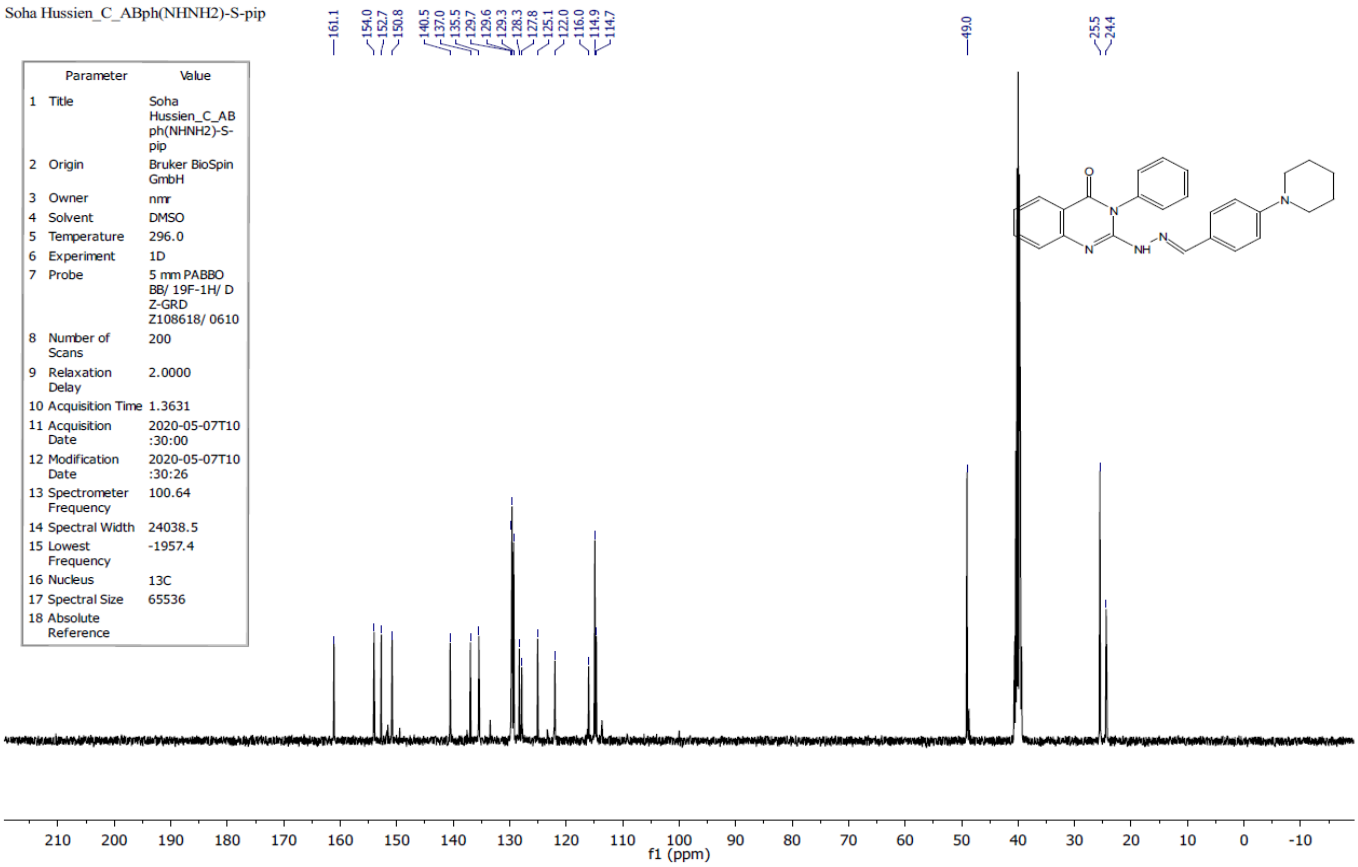
Soha Hussien_C_ABph(NHNH2)-S-pip
Parameter Value
1 Title Soha Hussien_C_ABph(NHNH2)-S-pip
2 Origin Bruker BioSpin GmbH
3 Owner nmr
4 Solvent DMSO
5 Temperature 296.0
6 Experiment 1D
7 Probe 5 mm PABBO BB/ 19F-1H/ D Z-GRD Z108618/ 0610
8 Number of Scans 200
9 Relaxation Delay 2.0000
10 Acquisition Time 1.3631
11 Acquisition Date 2020-05-07T10:30:00
12 Modification Date 2020-05-07T10:30:26
13 Spectrometer Frequency 100.64
14 Spectral Width 24038.5
15 Lowest Frequency -1957.4
16 Nucleus 13C
17 Spectral Size 65536
18 Absolute Reference
161.1 154.0 152.7 150.8 140.5 137.0 135.5 129.7 129.6 129.3 128.3 127.8 125.1 122.0 116.0 114.9 114.7 49.0 25.5 24.4
O N N NH N N
210 200 190 180 170 160 150 140 130 120 110 100 90 80 70 60 50 40 30 20 10 0 -10
f1 (ppm)

Compound 3f

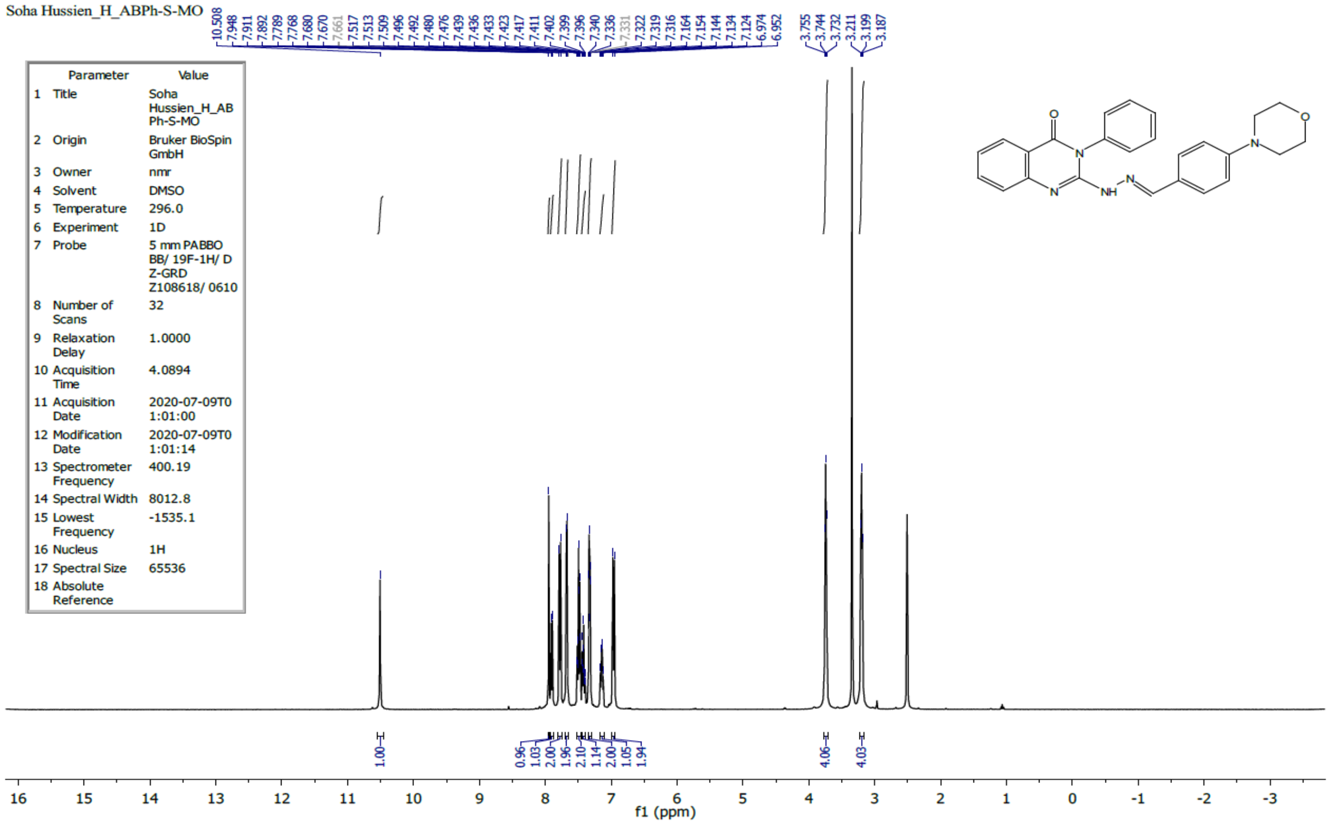

Soha Hussien_H_ABPh-S-MO
10.508
7.948
7.911
7.892
7.789
7.768
7.680
7.670
7.661
7.517
7.513
7.509
7.496
7.492
7.480
7.476
7.439
7.436
7.433
7.423
7.417
7.411
7.402
7.399
7.396
7.340
7.336
7.331
7.322
7.319
7.316
7.164
7.154
7.144
7.134
7.124
6.974
6.952
3.755
3.744
3.732
3.211
3.199
3.187
Parameter Value
1 Title Soha Hussien_H_ABPh-S-MO
2 Origin Bruker BioSpin GmbH
3 Owner nmr
4 Solvent DMSO
5 Temperature 296.0
6 Experiment 1D
7 Probe 5 mm PABBO BB/ 19F-1H/ D Z-GRD Z108618/ 0610
8 Number of Scans 32
9 Relaxation Delay 1.0000
10 Acquisition Time 4.0894
11 Acquisition Date 2020-07-09T01:01:00
12 Modification Date 2020-07-09T01:01:14
13 Spectrometer Frequency 400.19
14 Spectral Width 8012.8
15 Lowest Frequency -1535.1
16 Nucleus 1H
17 Spectral Size 65536
18 Absolute Reference
O
N
N
NH
N
N
O
1.00
0.96
1.03
2.00
1.96
2.10
1.14
2.00
1.05
1.94
4.06
4.03
16 15 14 13 12 11 10 9 8 7 6 5 4 3 2 1 0 -1 -2 -3
f1 (ppm)

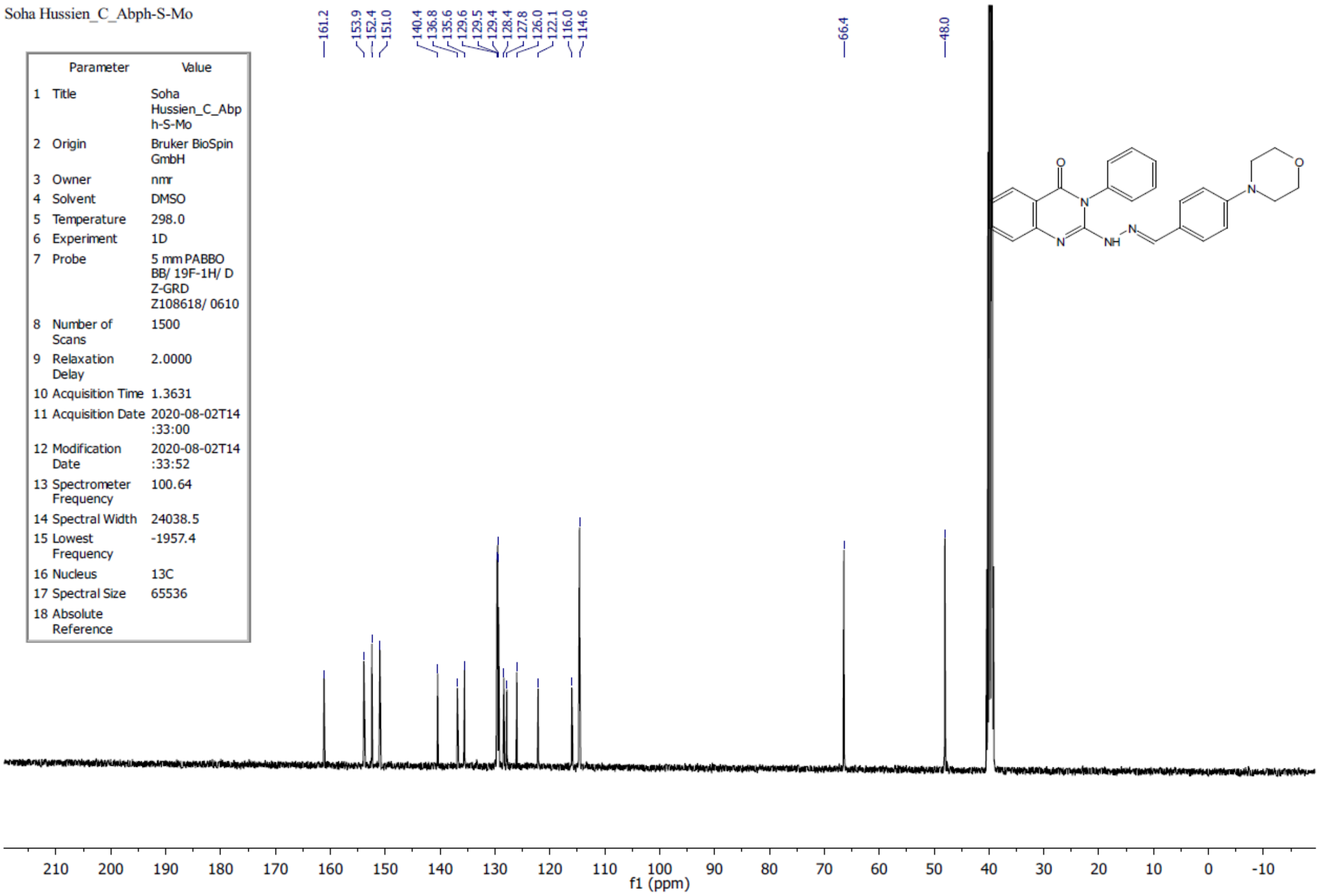

Soha Hussien_C_Abph-S-Mo
161.2
153.9
152.4
151.0
140.4
136.8
135.6
129.6
129.5
129.4
128.4
127.8
126.0
122.1
116.0
114.6
66.4
48.0
Parameter Value
1 Title Soha Hussien_C_Abph-S-Mo
2 Origin Bruker BioSpin GmbH
3 Owner nmr
4 Solvent DMSO
5 Temperature 298.0
6 Experiment 1D
7 Probe 5 mm PABBO BB/ 19F-1H/ D Z-GRD Z108618/ 0610
8 Number of Scans 1500
9 Relaxation Delay 2.0000
10 Acquisition Time 1.3631
11 Acquisition Date 2020-08-02T14:33:00
12 Modification Date 2020-08-02T14:33:52
13 Spectrometer Frequency 100.64
14 Spectral Width 24038.5
15 Lowest Frequency -1957.4
16 Nucleus 13C
17 Spectral Size 65536
18 Absolute Reference
O
N
N
NH
N
N
O
210 200 190 180 170 160 150 140 130 120 110 100 90 80 70 60 50 40 30 20 10 0 -10
f1 (ppm)

Compound 3g

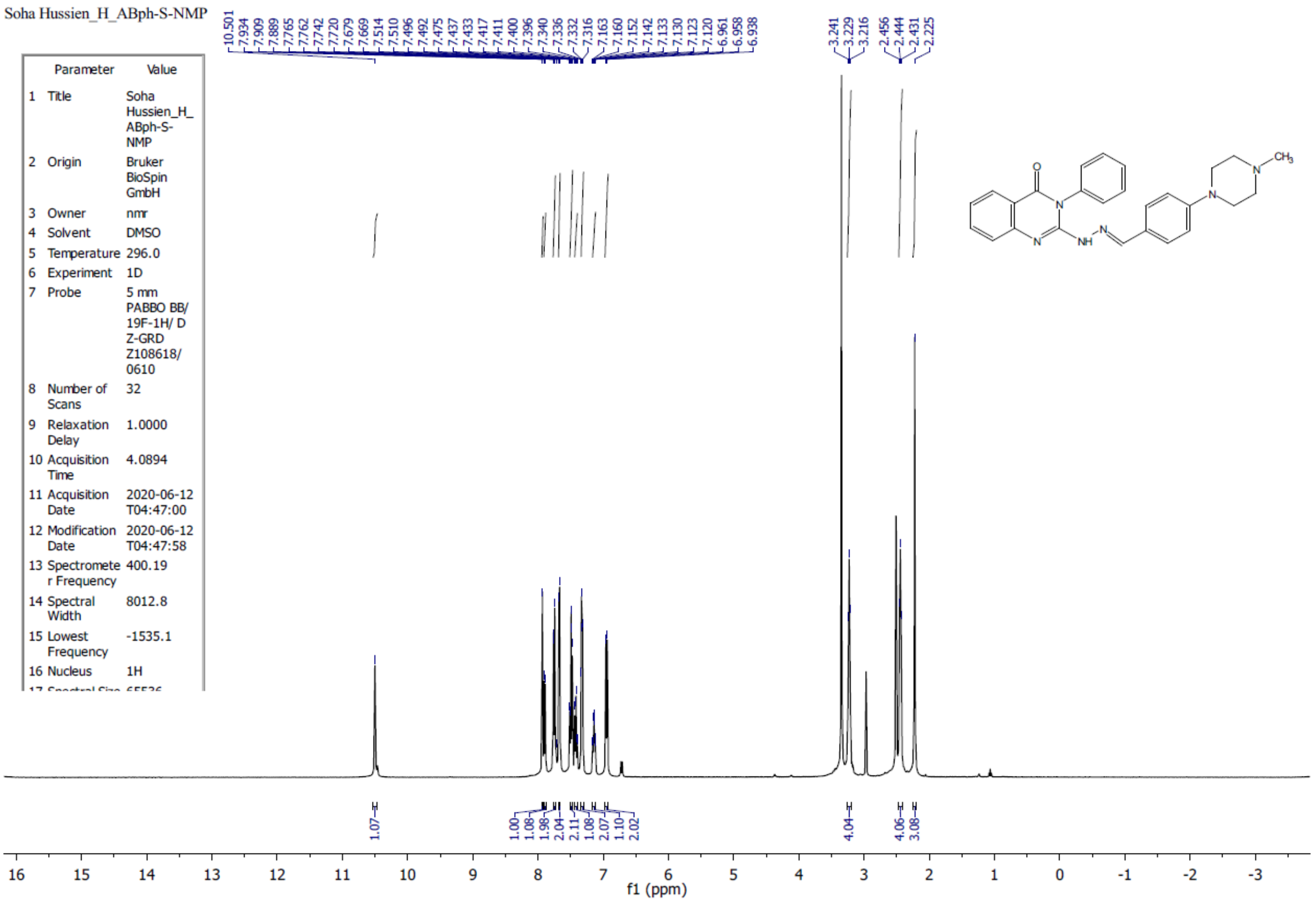
Soha Hussien_H_ABph-S-NMP
10.501
7.934
7.909
7.889
7.765
7.762
7.742
7.720
7.679
7.669
7.514
7.510
7.496
7.492
7.475
7.437
7.433
7.417
7.411
7.400
7.396
7.340
7.336
7.332
7.316
7.163
7.160
7.152
7.142
7.133
7.130
7.123
7.120
6.961
6.958
6.938
3.241
3.229
3.216
2.456
2.444
2.431
2.225
Parameter Value
1 Title Soha Hussien_H_ABph-S-NMP
2 Origin Bruker BioSpin GmbH
3 Owner nmr
4 Solvent DMSO
5 Temperature 296.0
6 Experiment 1D
7 Probe 5 mm PABBO BB/ 19F-1H/ D Z-GRD Z108618/ 0610
8 Number of Scans 32
9 Relaxation Delay 1.0000
10 Acquisition Time 4.0894
11 Acquisition Date 2020-06-12 T04:47:00
12 Modification Date 2020-06-12 T04:47:58
13 Spectrometer Frequency 400.19
14 Spectral Width 8012.8
15 Lowest Frequency -1535.1
16 Nucleus 1H
O
N
N
NH
N
N
N
CH3
1.07
1.00
1.08
1.98
2.04
2.11
1.08
2.07
1.10
2.02
4.04
4.06
3.08
f1 (ppm)

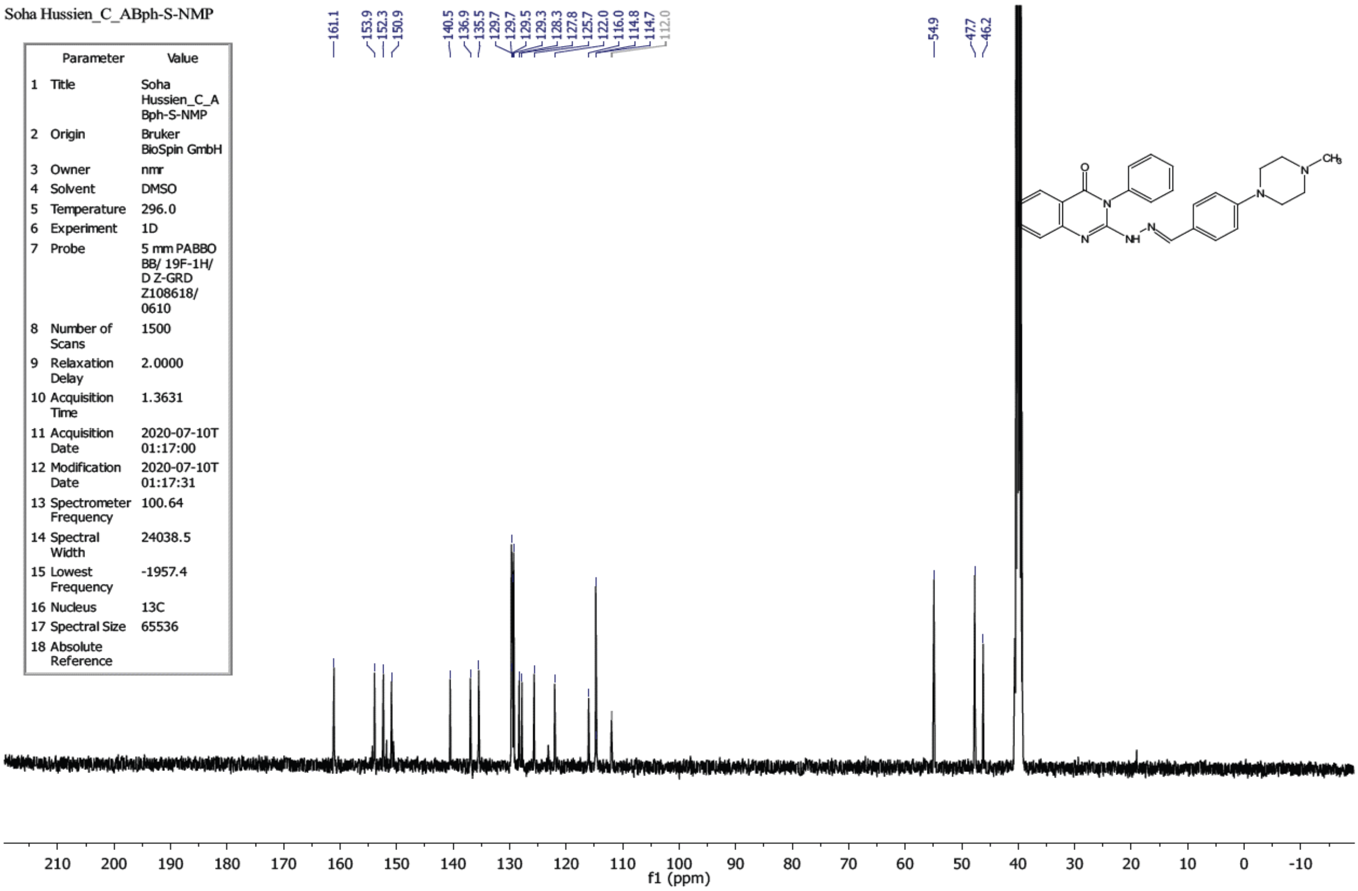
Soha Hussien_C_ABph-S-NMP
161.1
153.9
152.3
150.9
140.5
136.9
135.5
129.7
129.7
129.5
129.3
128.3
127.8
125.7
122.0
116.0
114.8
114.7
112.0
54.9
47.7
46.2
Parameter Value
1 Title Soha Hussien_C_ABph-S-NMP
2 Origin Bruker BioSpin GmbH
3 Owner nmr
4 Solvent DMSO
5 Temperature 296.0
6 Experiment 1D
7 Probe 5 mm PABBO BB/ 19F-1H/ D Z-GRD Z108618/ 0610
8 Number of Scans 1500
9 Relaxation Delay 2.0000
10 Acquisition Time 1.3631
11 Acquisition Date 2020-07-10T 01:17:00
12 Modification Date 2020-07-10T 01:17:31
13 Spectrometer Frequency 100.64
14 Spectral Width 24038.5
15 Lowest Frequency -1957.4
16 Nucleus 13C
17 Spectral Size 65536
18 Absolute Reference
O
N
N
NH
N
N
N
CH3
f1 (ppm)

Compound 6a

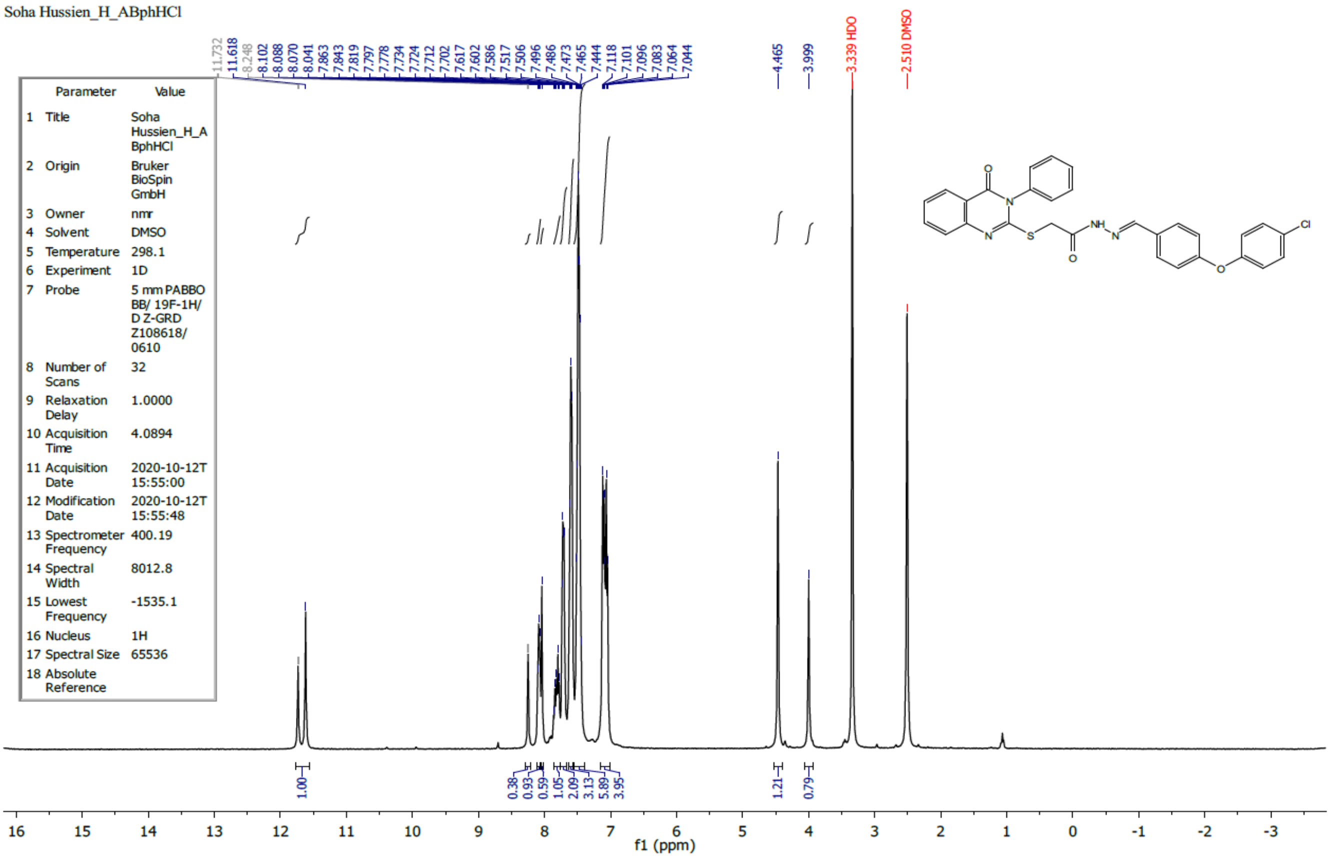
Soha Hussien_H_ABphHCl
11.732 11.618 8.248 8.102 8.088 8.070 8.041 7.863 7.843 7.819 7.797 7.778 7.734 7.724 7.712 7.702 7.617 7.602 7.586 7.517 7.506 7.496 7.486 7.473 7.465 7.444 7.118 7.101 7.096 7.083 7.064 7.044
4.465
3.999
3.339 HDO
2.510 DMSO
Parameter Value
1 Title Soha Hussien_H_ABphHCl
2 Origin Bruker BioSpin GmbH
3 Owner nmr
4 Solvent DMSO
5 Temperature 298.1
6 Experiment 1D
7 Probe 5 mm PABBO BB/ 19F-1H/ D Z-GRD Z108618/ 0610
8 Number of Scans 32
9 Relaxation Delay 1.0000
10 Acquisition Time 4.0894
11 Acquisition Date 2020-10-12T 15:55:00
12 Modification Date 2020-10-12T 15:55:48
13 Spectrometer Frequency 400.19
14 Spectral Width 8012.8
15 Lowest Frequency -1535.1
16 Nucleus 1H
17 Spectral Size 65536
18 Absolute Reference
1.00
0.38 0.93 0.59 1.05 2.09 3.13 5.89 3.95
1.21
0.79
f1 (ppm)

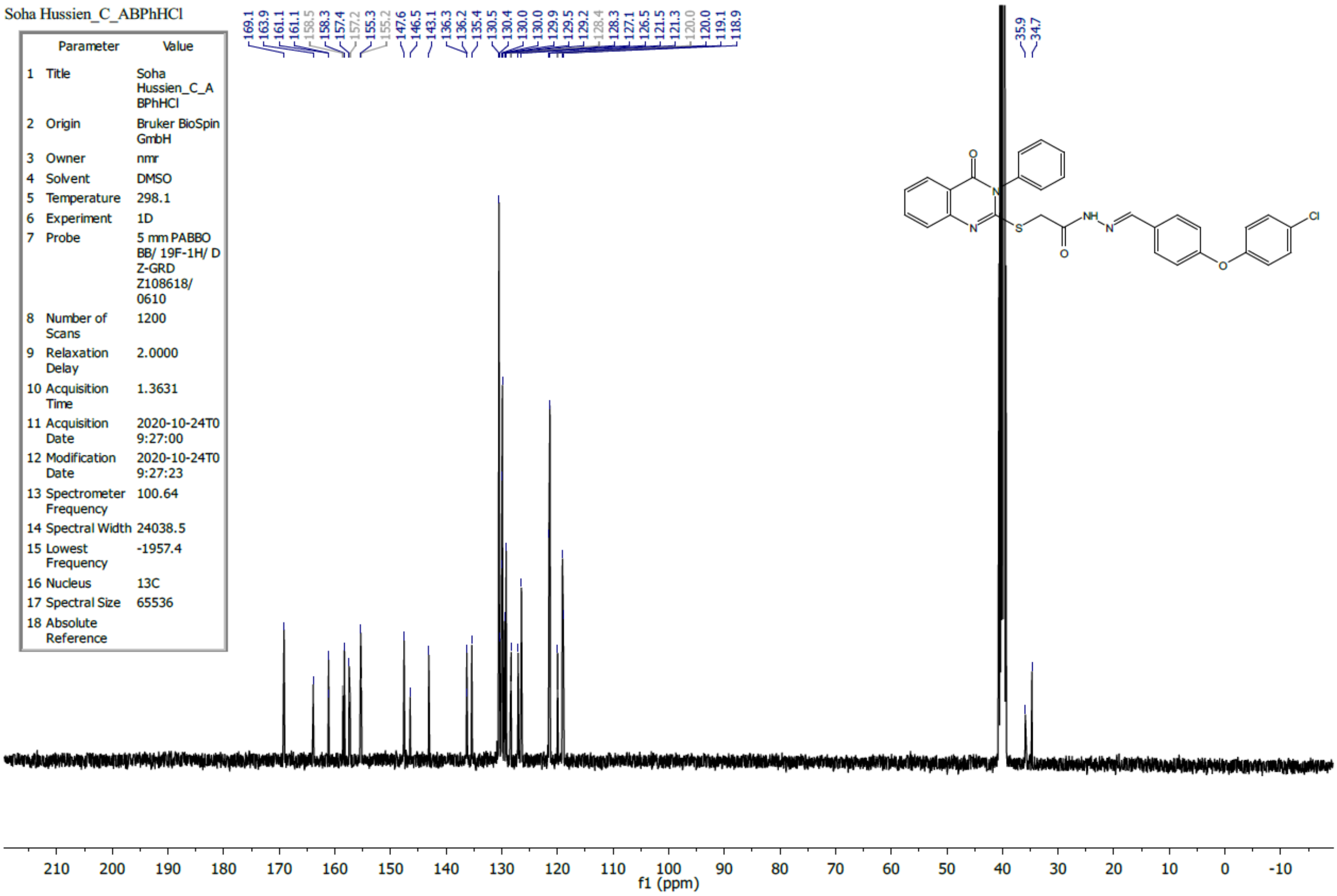
Soha Hussien_C_ABPhHCl
169.1 163.9 161.1 161.1 158.5 158.3 157.4 157.2 155.3 155.2 147.6 146.5 143.1 136.3 136.2 135.4 130.5 130.4 130.0 130.0 129.9 129.5 129.2 128.4 128.3 127.1 126.5 121.5 121.3 120.0 120.0 119.1 118.9
35.9 34.7
Parameter Value
1 Title Soha Hussien_C_ABPhHCl
2 Origin Bruker BioSpin GmbH
3 Owner nmr
4 Solvent DMSO
5 Temperature 298.1
6 Experiment 1D
7 Probe 5 mm PABBO BB/ 19F-1H/ D Z-GRD Z108618/ 0610
8 Number of Scans 1200
9 Relaxation Delay 2.0000
10 Acquisition Time 1.3631
11 Acquisition Date 2020-10-24T09:27:00
12 Modification Date 2020-10-24T09:27:23
13 Spectrometer Frequency 100.64
14 Spectral Width 24038.5
15 Lowest Frequency -1957.4
16 Nucleus 13C
17 Spectral Size 65536
18 Absolute Reference
f1 (ppm)

Compound 6b

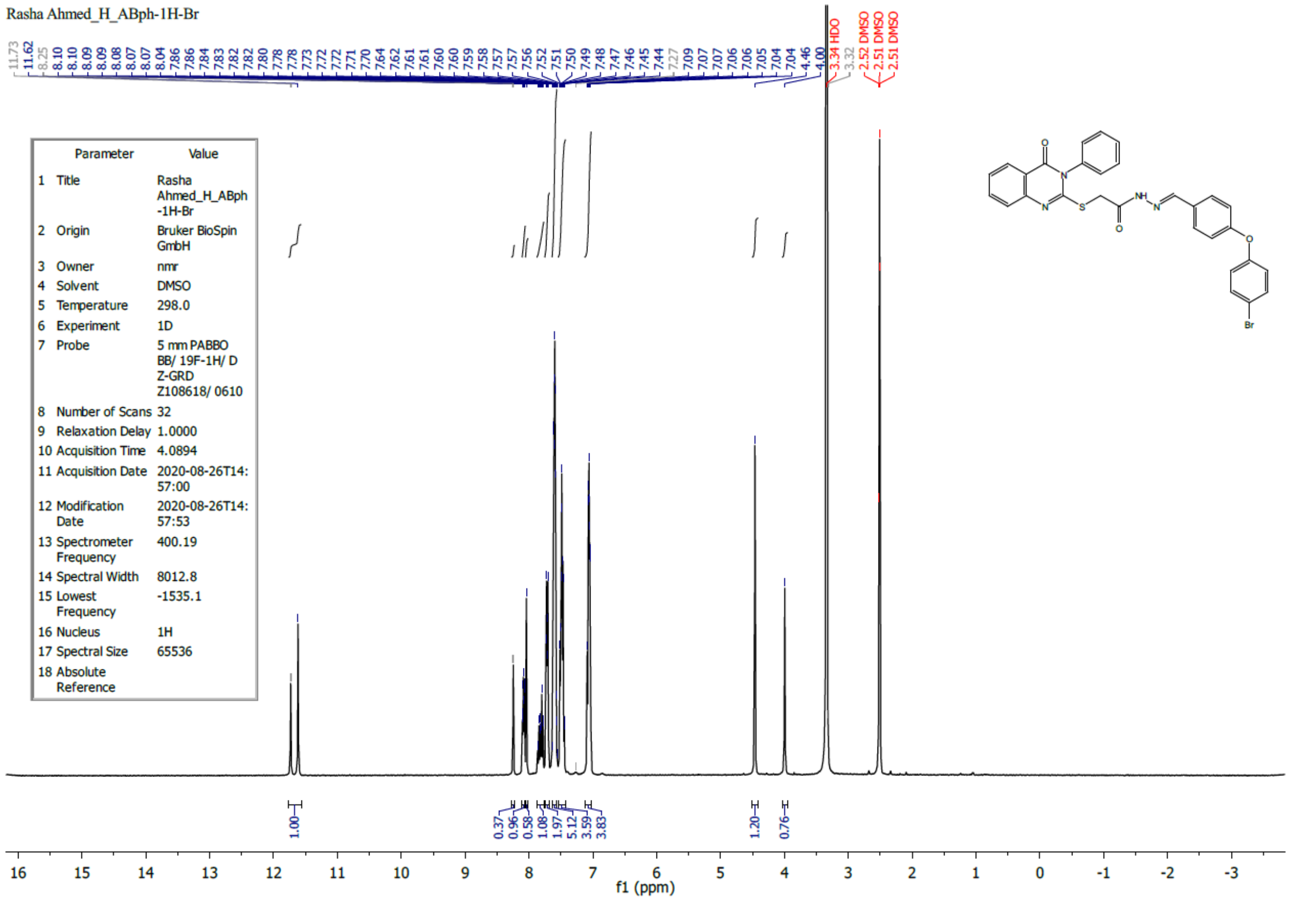

Rasha Ahmed_H_ABph-1H-Br
Parameter Value
1 Title Rasha Ahmed_H_ABph-1H-Br
2 Origin Bruker BioSpin GmbH
3 Owner nmr
4 Solvent DMSO
5 Temperature 298.0
6 Experiment 1D
7 Probe 5 mm PABBO BB/ 19F-1H/ D Z-GRD Z108618/ 0610
8 Number of Scans 32
9 Relaxation Delay 1.0000
10 Acquisition Time 4.0894
11 Acquisition Date 2020-08-26T14:57:00
12 Modification Date 2020-08-26T14:57:53
13 Spectrometer Frequency 400.19
14 Spectral Width 8012.8
15 Lowest Frequency -1535.1
16 Nucleus 1H
17 Spectral Size 65536
18 Absolute Reference
3.34 HDO
2.52 DMSO
2.51 DMSO
2.51 DMSO
f1 (ppm)

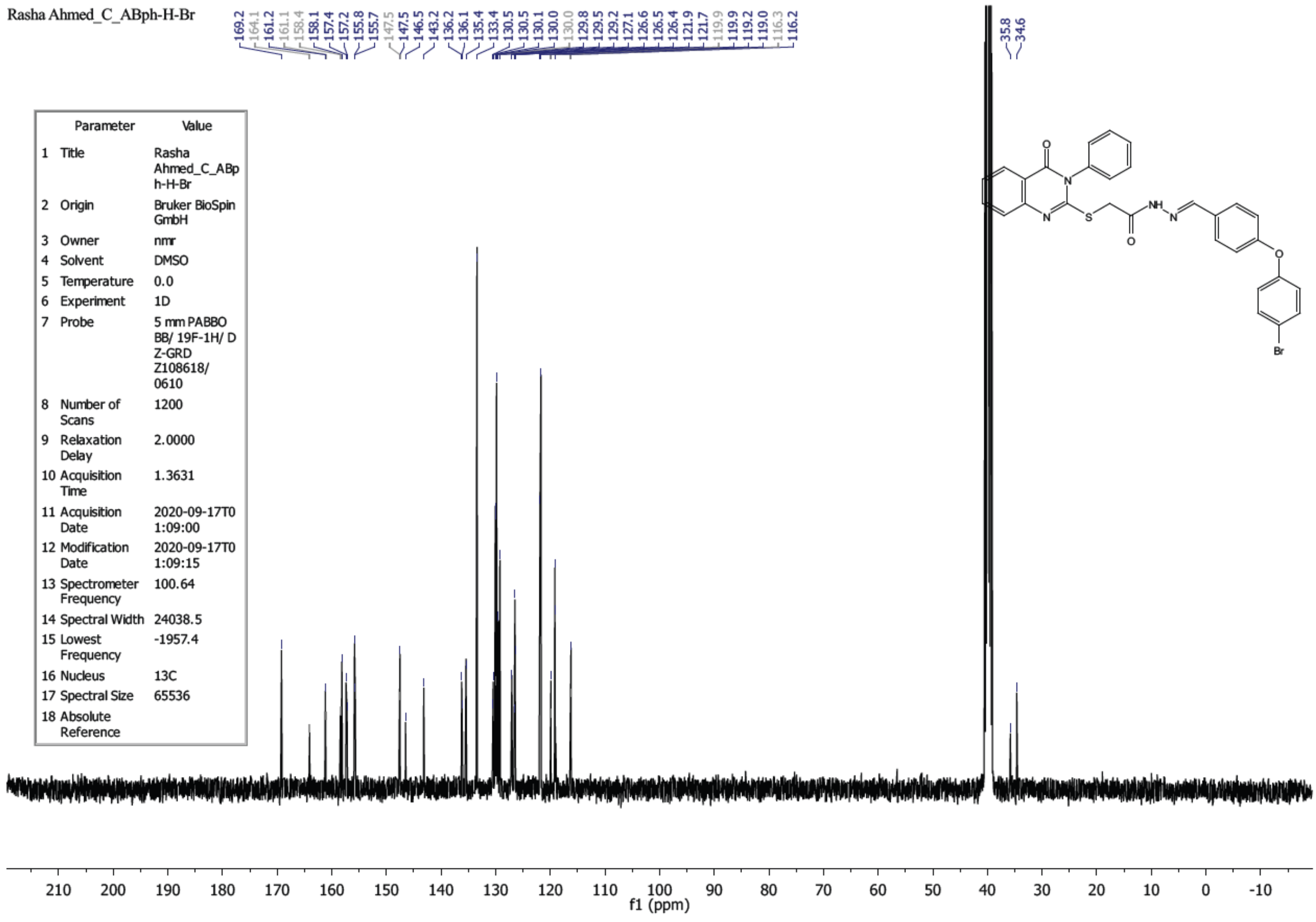

Rasha Ahmed_C_ABph-H-Br
Parameter Value
1 Title Rasha Ahmed_C_ABph-H-Br
2 Origin Bruker BioSpin GmbH
3 Owner nmr
4 Solvent DMSO
5 Temperature 0.0
6 Experiment 1D
7 Probe 5 mm PABBO BB/ 19F-1H/ D Z-GRD Z108618/ 0610
8 Number of Scans 1200
9 Relaxation Delay 2.0000
10 Acquisition Time 1.3631
11 Acquisition Date 2020-09-17T01:09:00
12 Modification Date 2020-09-17T01:09:15
13 Spectrometer Frequency 100.64
14 Spectral Width 24038.5
15 Lowest Frequency -1957.4
16 Nucleus 13C
17 Spectral Size 65536
18 Absolute Reference
f1 (ppm)

Compound 6c

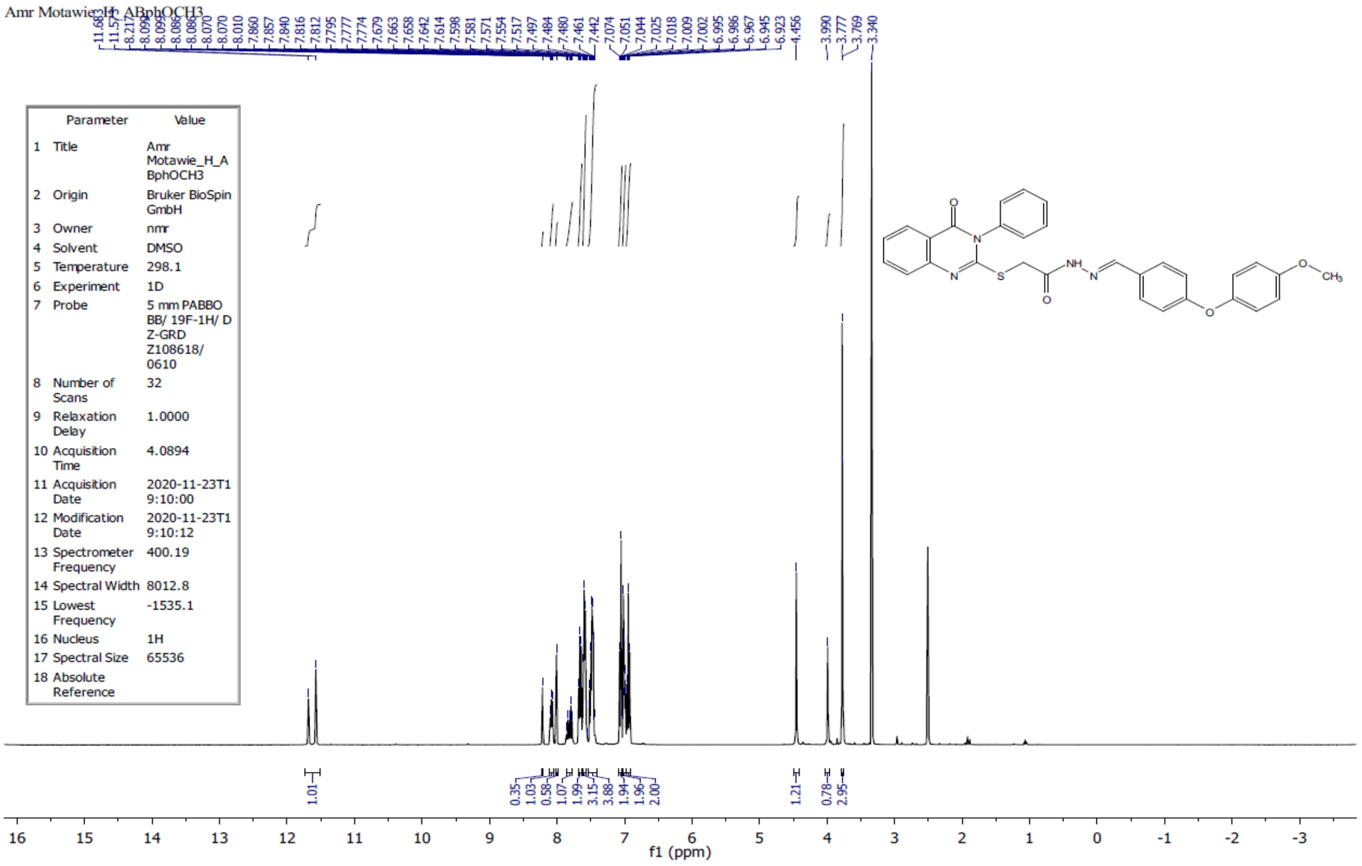
Amr Motawie_H_ABphOCH3
11.681 11.575 8.217 8.099 8.095 8.086 8.086 8.070 8.070 8.010 7.860 7.857 7.840 7.816 7.812 7.795 7.777 7.774 7.679 7.663 7.658 7.642 7.614 7.598 7.581 7.571 7.554 7.517 7.497 7.484 7.480 7.461 7.442 7.074 7.051 7.044 7.025 7.018 7.009 7.002 6.995 6.986 6.967 6.945 6.923 4.456 3.990 3.777 3.769 3.340
Parameter Value
1 Title Amr Motawie_H_ABphOCH3
2 Origin Bruker BioSpin GmbH
3 Owner nmr
4 Solvent DMSO
5 Temperature 298.1
6 Experiment 1D
7 Probe 5 mm PABBO BB/ 19F-1H/ D Z-GRD Z108618/ 0610
8 Number of Scans 32
9 Relaxation Delay 1.0000
10 Acquisition Time 4.0894
11 Acquisition Date 2020-11-23T19:10:00
12 Modification Date 2020-11-23T19:10:12
13 Spectrometer Frequency 400.19
14 Spectral Width 8012.8
15 Lowest Frequency -1535.1
16 Nucleus 1H
17 Spectral Size 65536
18 Absolute Reference
1.01 0.35 1.03 0.58 1.07 1.99 3.15 3.88 1.94 1.96 2.00 1.21 0.78 2.95
16 15 14 13 12 11 10 9 8 7 6 5 4 3 2 1 0 -1 -2 -3
f1 (ppm)

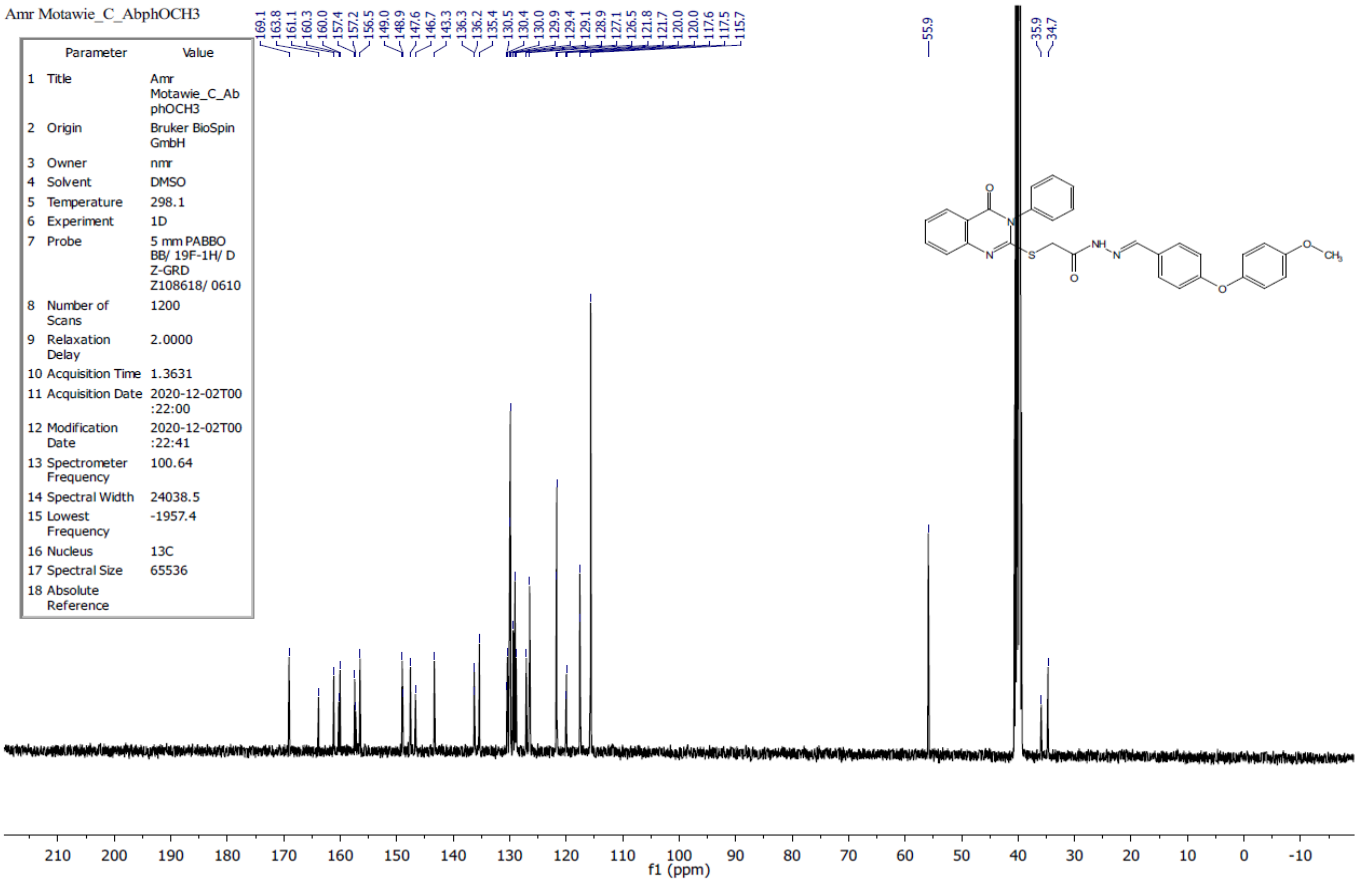
Amr Motawie_C_AbphOCH3
169.1 163.8 161.1 160.3 160.0 157.4 157.2 156.5 149.0 148.9 147.6 146.7 143.3 136.3 136.2 135.4 130.5 130.4 130.0 129.9 129.4 129.1 128.9 127.1 126.5 121.8 121.7 120.0 120.0 117.6 117.5 115.7
55.9
35.9 34.7
Parameter Value
1 Title Amr Motawie_C_AbphOCH3
2 Origin Bruker BioSpin GmbH
3 Owner nmr
4 Solvent DMSO
5 Temperature 298.1
6 Experiment 1D
7 Probe 5 mm PABBO BB/ 19F-1H/ D Z-GRD Z108618/ 0610
8 Number of Scans 1200
9 Relaxation Delay 2.0000
10 Acquisition Time 1.3631
11 Acquisition Date 2020-12-02T00:22:00
12 Modification Date 2020-12-02T00:22:41
13 Spectrometer Frequency 100.64
14 Spectral Width 24038.5
15 Lowest Frequency -1957.4
16 Nucleus 13C
17 Spectral Size 65536
18 Absolute Reference
210 200 190 180 170 160 150 140 130 120 110 100 90 80 70 60 50 40 30 20 10 0 -10
f1 (ppm)

Compound 6d

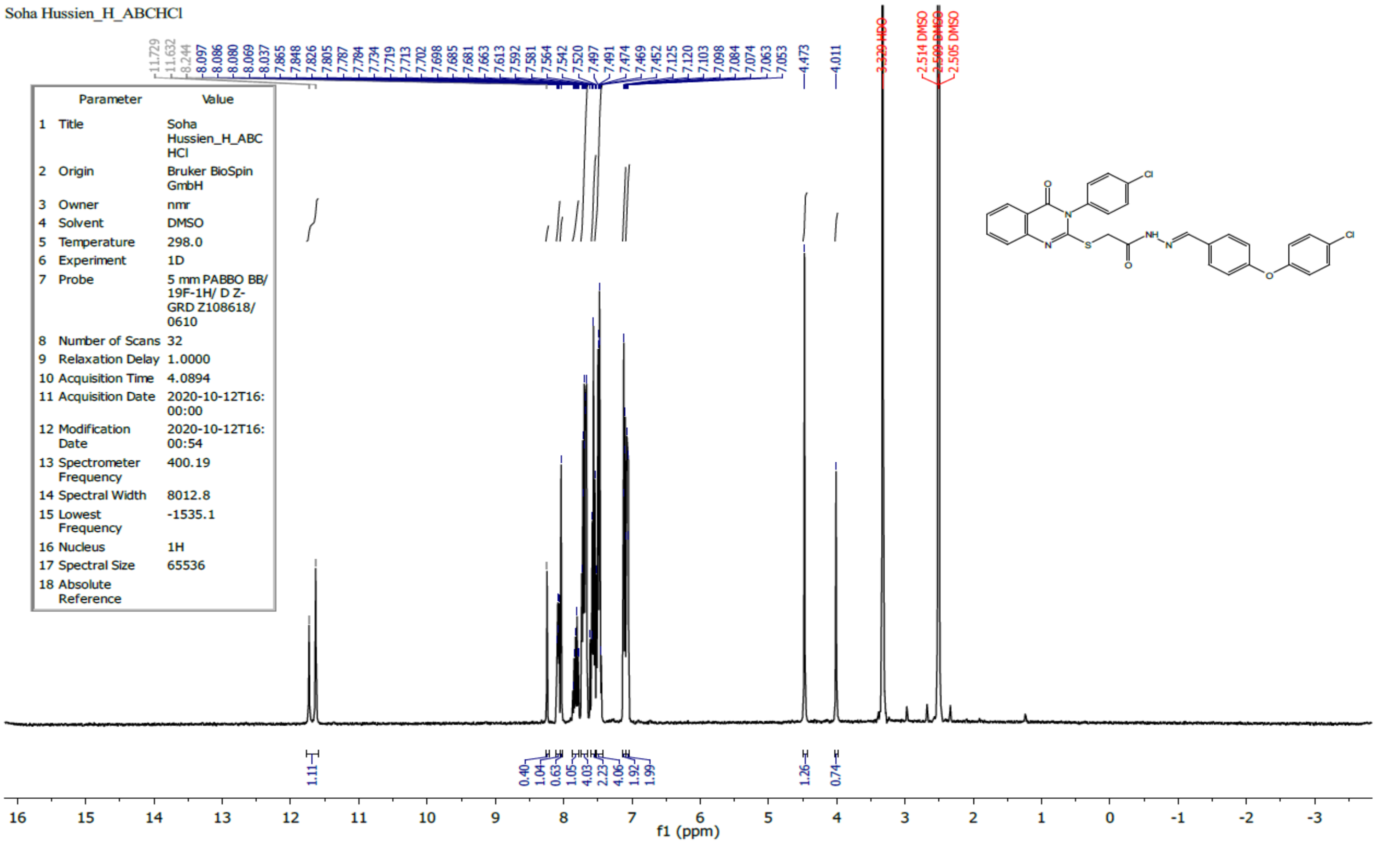
Soha Hussien_H_ABCHCl
11.729
11.632
8.244
8.097
8.086
8.080
8.069
8.037
7.865
7.848
7.826
7.805
7.787
7.784
7.734
7.719
7.713
7.702
7.698
7.685
7.681
7.663
7.613
7.592
7.581
7.564
7.542
7.520
7.497
7.491
7.474
7.469
7.452
7.125
7.120
7.103
7.098
7.084
7.074
7.063
7.053
4.473
4.011
3.320 HDO
2.514 DMSO
2.509 DMSO
2.505 DMSO
Parameter
Value
1 Title
Soha Hussien_H_ABCHCl
2 Origin
Bruker BioSpin GmbH
3 Owner
nmr
4 Solvent
DMSO
5 Temperature
298.0
6 Experiment
1D
7 Probe
5 mm PABBO BB/ 19F-1H/ D Z-GRD Z108618/ 0610
8 Number of Scans
32
9 Relaxation Delay
1.0000
10 Acquisition Time
4.0894
11 Acquisition Date
2020-10-12T16:00:00
12 Modification Date
2020-10-12T16:00:54
13 Spectrometer Frequency
400.19
14 Spectral Width
8012.8
15 Lowest Frequency
-1535.1
16 Nucleus
1H
17 Spectral Size
65536
18 Absolute Reference
Cl
O
N
N
S
NH
N
O
O
Cl
1.11
0.40
1.04
0.63
1.05
4.03
2.23
4.06
1.92
1.99
1.26
0.74
16
15
14
13
12
11
10
9
8
7
6
5
4
3
2
1
0
-1
-2
-3
f1 (ppm)

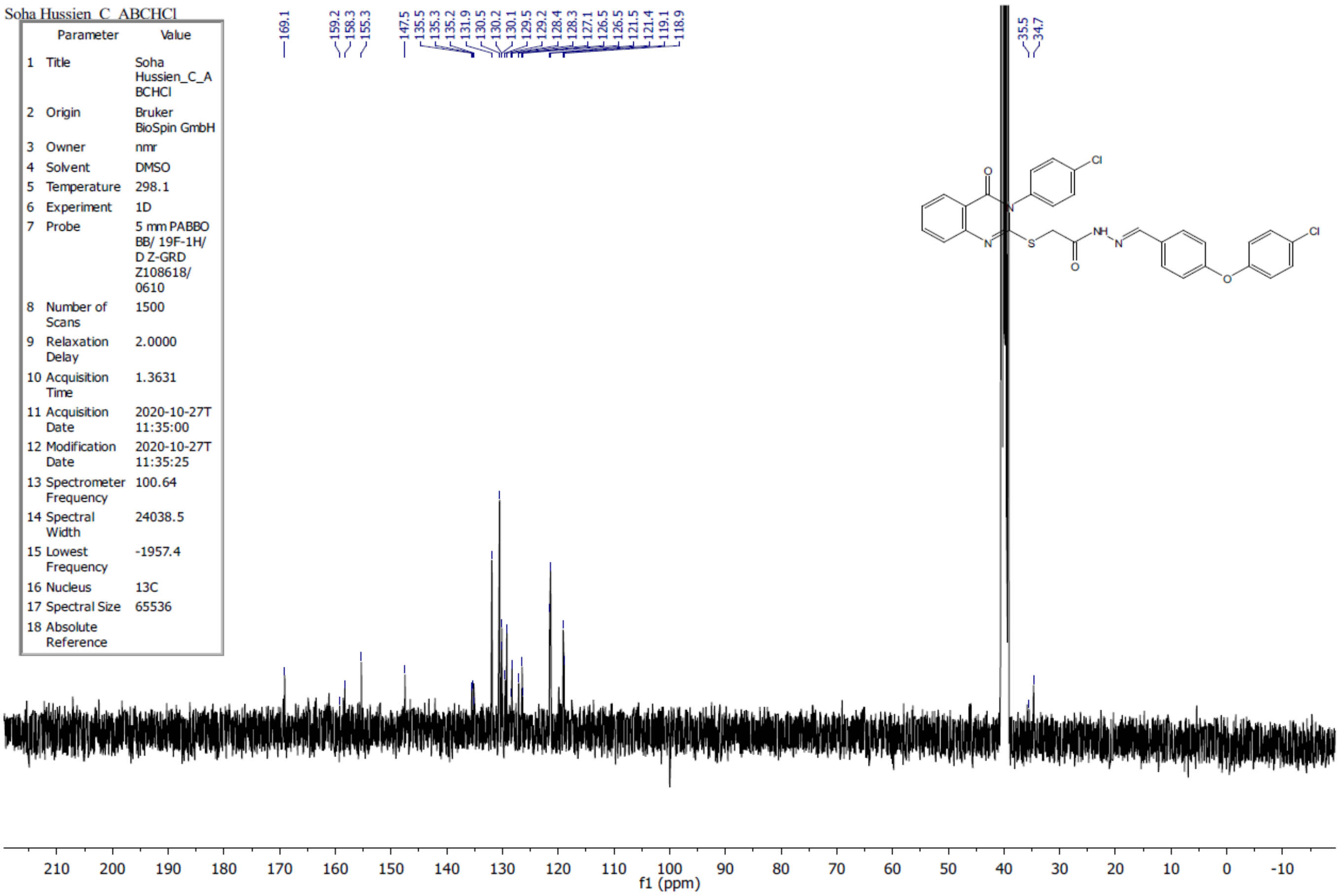
Soha Hussien_C_ABCHCl
169.1
159.2
158.3
155.3
147.5
135.5
135.3
135.2
131.9
130.5
130.2
130.1
129.5
129.2
128.4
128.3
127.1
126.5
126.5
121.5
121.4
119.1
118.9
35.5
34.7
Parameter
Value
1 Title
Soha Hussien_C_ABCHCl
2 Origin
Bruker BioSpin GmbH
3 Owner
nmr
4 Solvent
DMSO
5 Temperature
298.1
6 Experiment
1D
7 Probe
5 mm PABBO BB/ 19F-1H/ D Z-GRD Z108618/ 0610
8 Number of Scans
1500
9 Relaxation Delay
2.0000
10 Acquisition Time
1.3631
11 Acquisition Date
2020-10-27T11:35:00
12 Modification Date
2020-10-27T11:35:25
13 Spectrometer Frequency
100.64
14 Spectral Width
24038.5
15 Lowest Frequency
-1957.4
16 Nucleus
13C
17 Spectral Size
65536
18 Absolute Reference
Cl
O
N
N
S
NH
N
O
O
Cl
210
200
190
180
170
160
150
140
130
120
110
100
90
80
70
60
50
40
30
20
10
0
-10
f1 (ppm)

Compound 6e

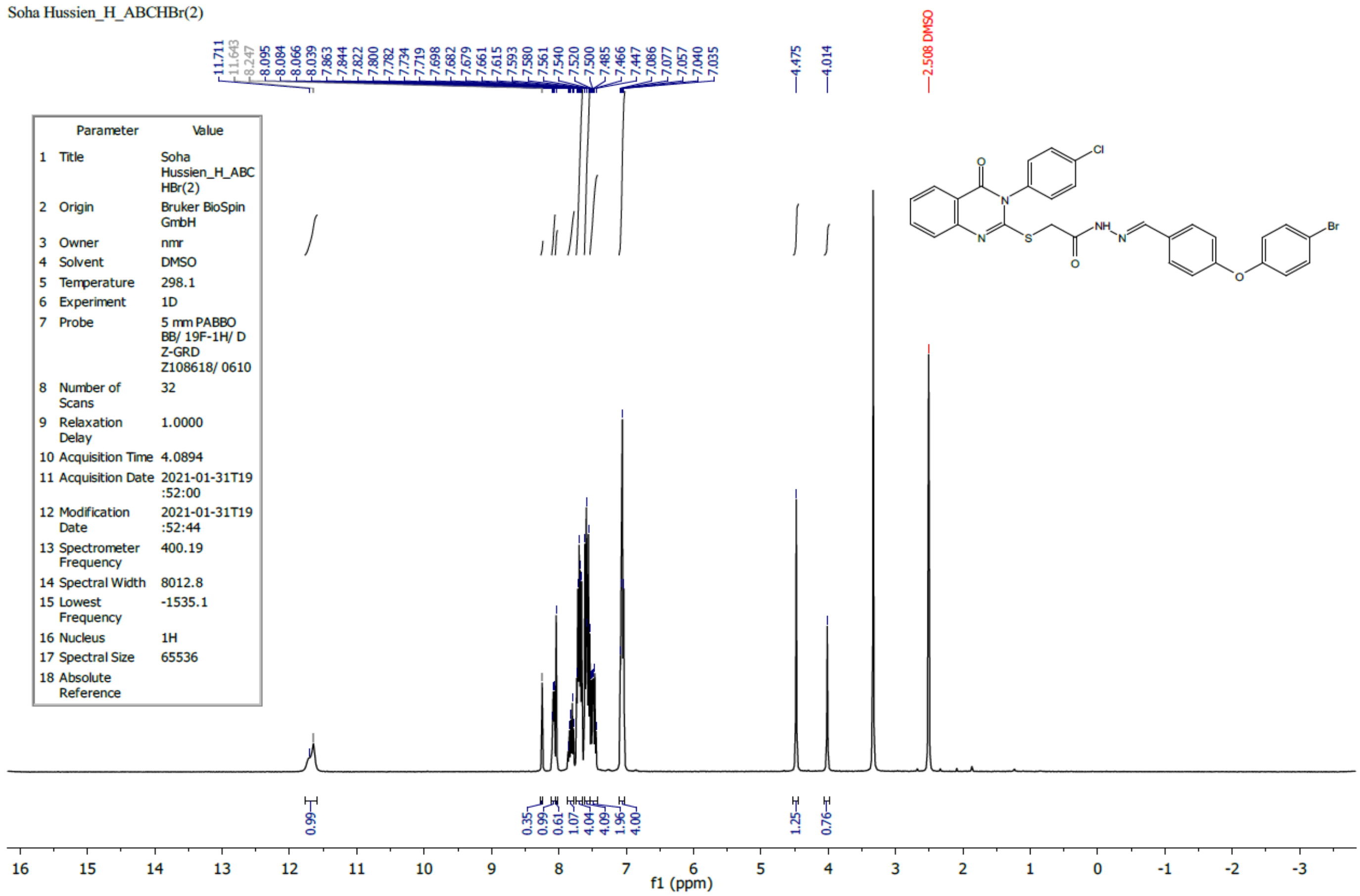

Soha Hussien_H_ABCHBr(2)
11.711
11.643
8.247
8.095
8.084
8.066
8.039
7.863
7.844
7.822
7.800
7.782
7.734
7.719
7.698
7.682
7.679
7.661
7.615
7.593
7.580
7.561
7.540
7.520
7.500
7.485
7.466
7.447
7.086
7.077
7.057
7.040
7.035
4.475
4.014
2.508 DMSO
Parameter Value
1 Title Soha Hussien_H_ABCHBr(2)
2 Origin Bruker BioSpin GmbH
3 Owner nmr
4 Solvent DMSO
5 Temperature 298.1
6 Experiment 1D
7 Probe 5 mm PABBO BB/ 19F-1H/ D Z-GRD Z108618/ 0610
8 Number of Scans 32
9 Relaxation Delay 1.0000
10 Acquisition Time 4.0894
11 Acquisition Date 2021-01-31T19:52:00
12 Modification Date 2021-01-31T19:52:44
13 Spectrometer Frequency 400.19
14 Spectral Width 8012.8
15 Lowest Frequency -1535.1
16 Nucleus 1H
17 Spectral Size 65536
18 Absolute Reference
O
N
N
S
Cl
NH
N
O
O
Br
0.99
0.35
0.99
0.61
1.07
4.04
4.09
1.96
4.00
1.25
0.76
f1 (ppm)

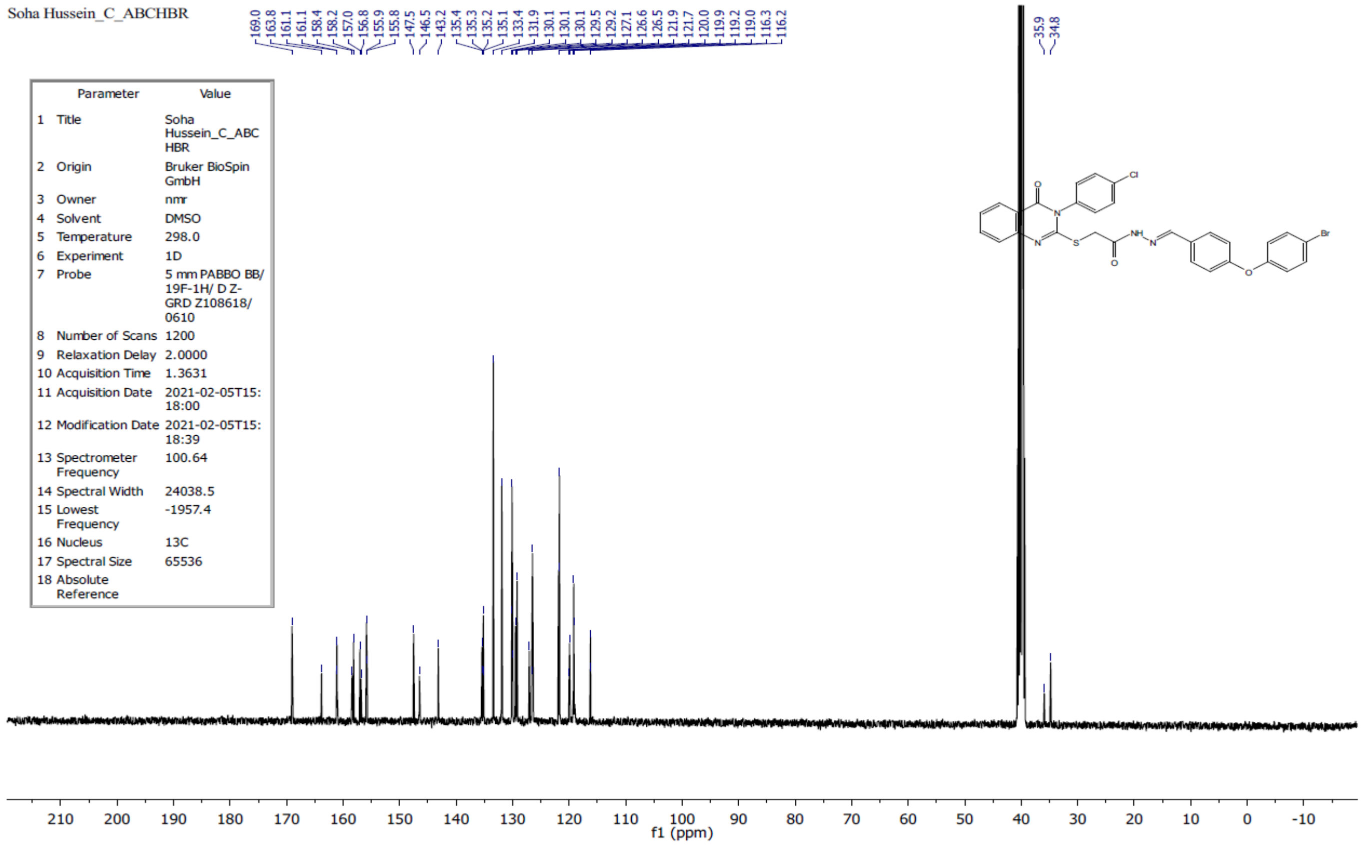

Soha Hussein_C_ABCHBR
169.0
163.8
161.1
161.1
158.4
158.2
157.0
156.8
155.9
155.8
147.5
146.5
143.2
135.4
135.3
135.2
135.1
133.4
131.9
130.1
130.1
130.1
130.1
129.5
129.2
127.1
126.6
126.5
121.9
121.7
120.0
119.9
119.2
119.0
116.3
116.2
35.9
34.8
Parameter Value
1 Title Soha Hussein_C_ABCHBR
2 Origin Bruker BioSpin GmbH
3 Owner nmr
4 Solvent DMSO
5 Temperature 298.0
6 Experiment 1D
7 Probe 5 mm PABBO BB/ 19F-1H/ D Z-GRD Z108618/ 0610
8 Number of Scans 1200
9 Relaxation Delay 2.0000
10 Acquisition Time 1.3631
11 Acquisition Date 2021-02-05T15:18:00
12 Modification Date 2021-02-05T15:18:39
13 Spectrometer Frequency 100.64
14 Spectral Width 24038.5
15 Lowest Frequency -1957.4
16 Nucleus 13C
17 Spectral Size 65536
18 Absolute Reference
f1 (ppm)

Compound 6f

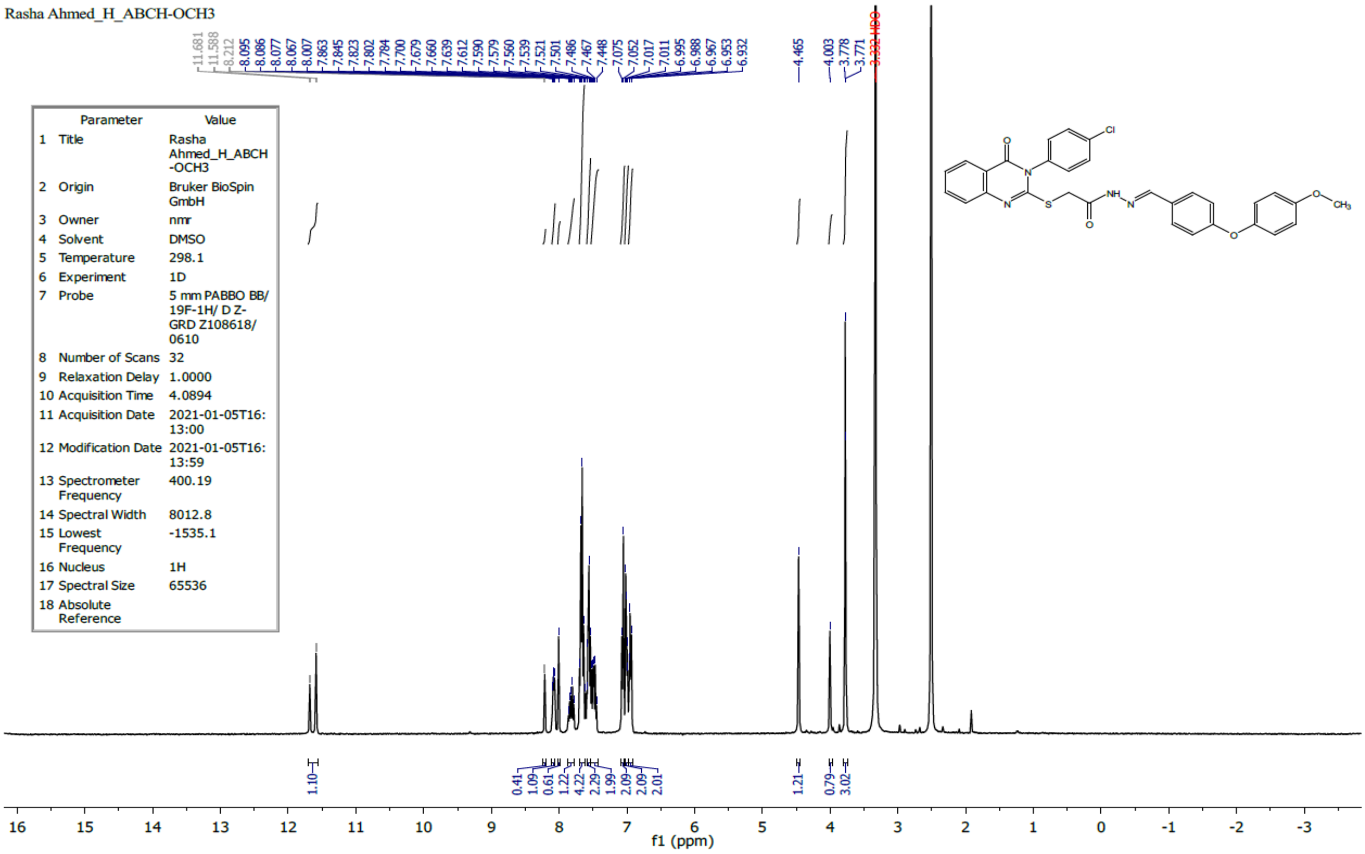
Rasha Ahmed_H_ABCH-OCH3
11.681
11.588
8.212
8.095
8.086
8.077
8.067
8.007
7.863
7.845
7.823
7.802
7.784
7.700
7.679
7.660
7.639
7.612
7.590
7.579
7.560
7.539
7.521
7.501
7.486
7.467
7.448
7.075
7.052
7.017
7.011
6.995
6.988
6.967
6.953
6.932
4.465
4.003
3.778
3.771
3.331 H2O
Parameter Value
1 Title Rasha Ahmed_H_ABCH-OCH3
2 Origin Bruker BioSpin GmbH
3 Owner nmr
4 Solvent DMSO
5 Temperature 298.1
6 Experiment 1D
7 Probe 5 mm PABBO BB/ 19F-1H/ D Z-GRD Z108618/ 0610
8 Number of Scans 32
9 Relaxation Delay 1.0000
10 Acquisition Time 4.0894
11 Acquisition Date 2021-01-05T16:13:00
12 Modification Date 2021-01-05T16:13:59
13 Spectrometer Frequency 400.19
14 Spectral Width 8012.8
15 Lowest Frequency -1535.1
16 Nucleus 1H
17 Spectral Size 65536
18 Absolute Reference
1.10
0.41
1.09
0.61
1.22
4.22
2.29
1.99
2.09
2.09
2.01
1.21
0.79
3.02
16 15 14 13 12 11 10 9 8 7 6 5 4 3 2 1 0 -1 -2 -3
f1 (ppm)
Cl
O
N
N
S
NH
N
O
O
O
CH3

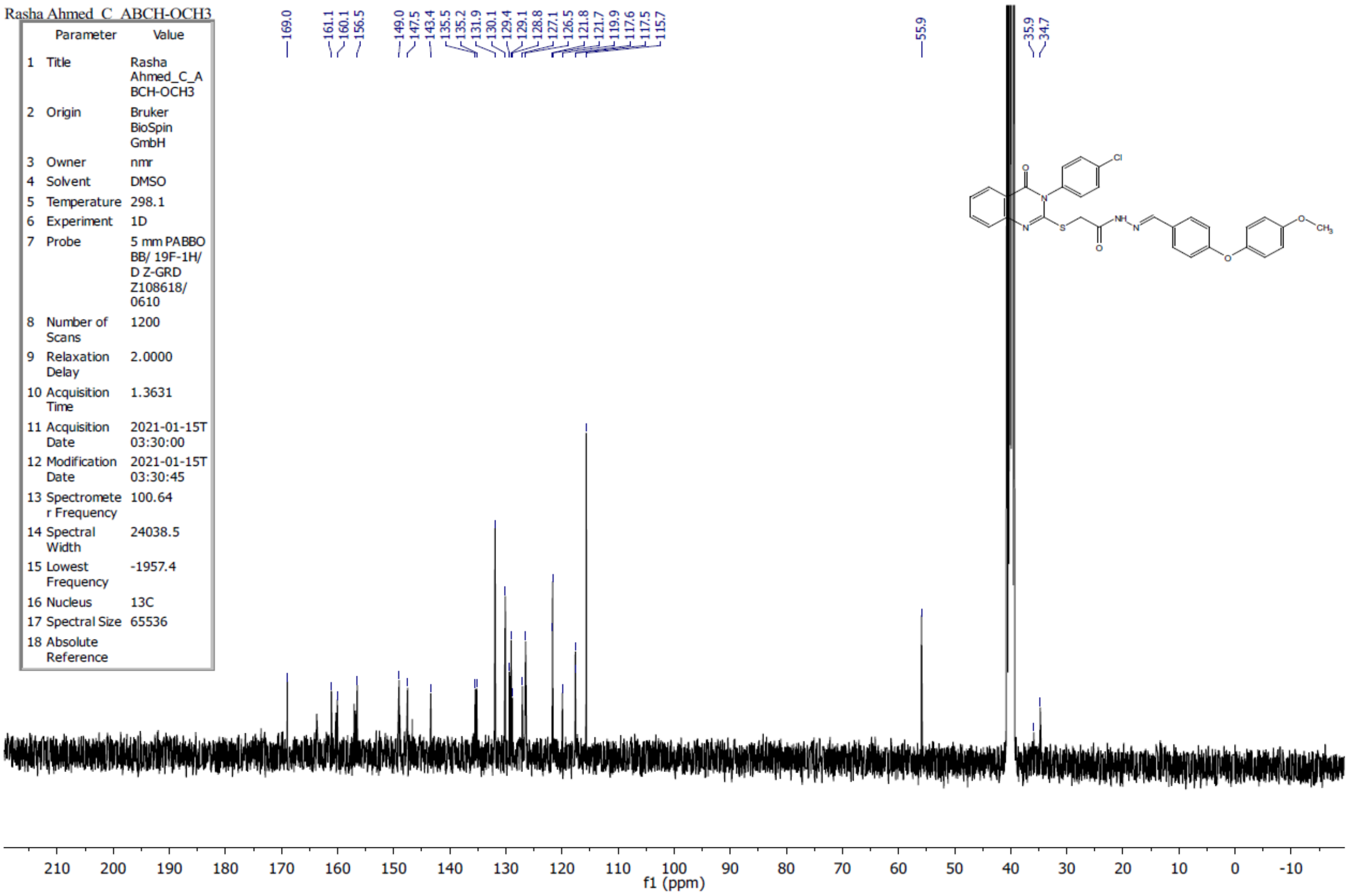
Rasha Ahmed_C_ABCH-OCH3
169.0
161.1
160.1
156.5
149.0
147.5
143.4
135.5
135.2
131.9
130.1
129.4
129.1
128.8
127.1
126.5
121.8
121.7
119.9
117.6
117.5
115.7
55.9
35.9
34.7
Parameter Value
1 Title Rasha Ahmed_C_ABCH-OCH3
2 Origin Bruker BioSpin GmbH
3 Owner nmr
4 Solvent DMSO
5 Temperature 298.1
6 Experiment 1D
7 Probe 5 mm PABBO BB/ 19F-1H/ D Z-GRD Z108618/ 0610
8 Number of Scans 1200
9 Relaxation Delay 2.0000
10 Acquisition Time 1.3631
11 Acquisition Date 2021-01-15T03:30:00
12 Modification Date 2021-01-15T03:30:45
13 Spectrometer Frequency 100.64
14 Spectral Width 24038.5
15 Lowest Frequency -1957.4
16 Nucleus 13C
17 Spectral Size 65536
18 Absolute Reference
210 200 190 180 170 160 150 140 130 120 110 100 90 80 70 60 50 40 30 20 10 0 -10
f1 (ppm)
Cl
O
N
N
S
NH
N
O
O
O
CH3

Compound 6g

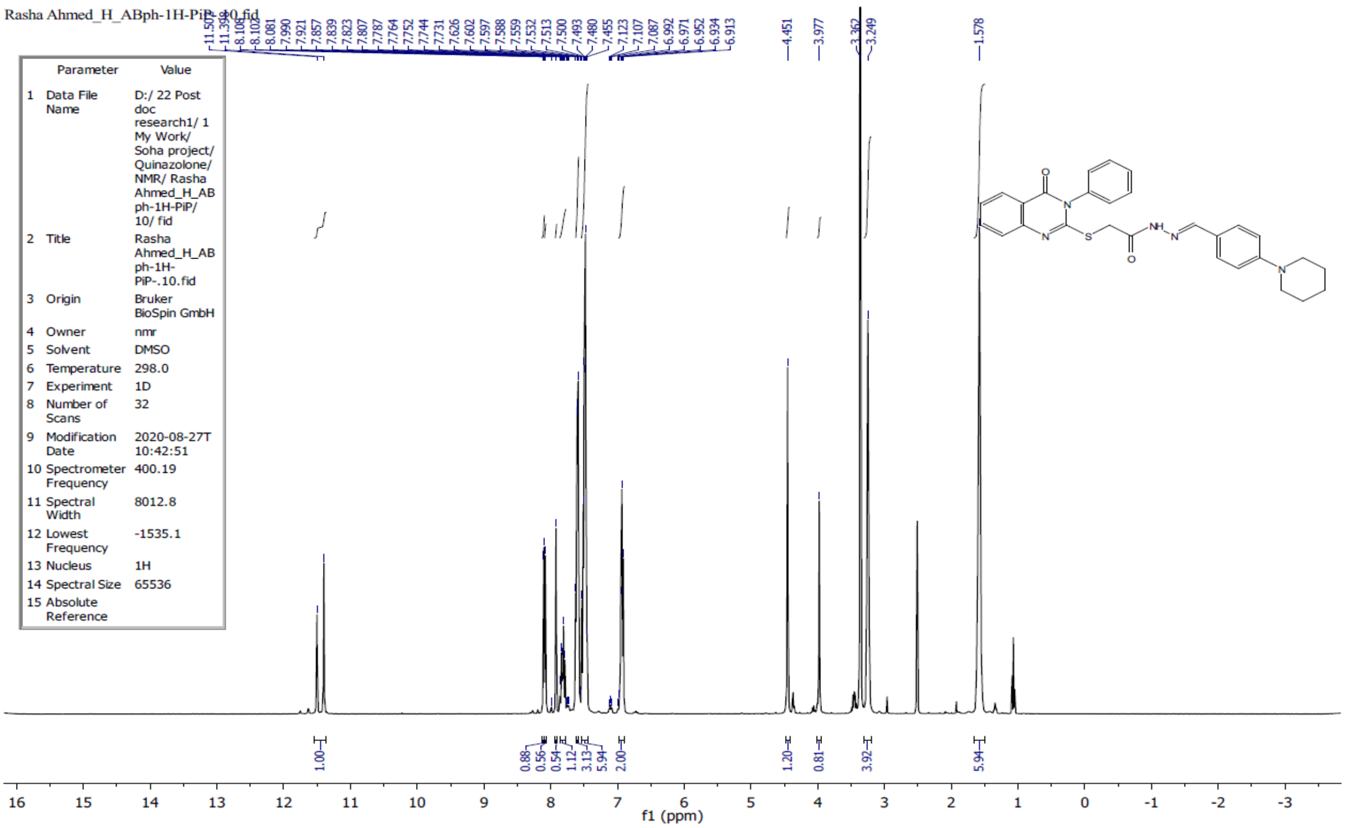
Rasha Ahmed_H_ABph-1H-PiP-10.fid
11.507 11.390 8.106 8.102 8.081 7.990 7.921 7.857 7.839 7.823 7.807 7.787 7.764 7.752 7.744 7.731 7.626 7.602 7.597 7.588 7.559 7.532 7.513 7.500 7.493 7.480 7.455 7.123 7.107 7.087 6.992 6.971 6.952 6.934 6.913
4.451 3.977 3.362 3.249 1.578
Parameter Value
1 Data File Name D:/ 22 Post doc research1/ 1 My Work/ Soha project/ Quinazolone/ NMR/ Rasha Ahmed_H_ABph-1H-PiP/ 10/ fid
2 Title Rasha Ahmed_H_ABph-1H-PiP-.10.fid
3 Origin Bruker BioSpin GmbH
4 Owner nmr
5 Solvent DMSO
6 Temperature 298.0
7 Experiment 1D
8 Number of Scans 32
9 Modification Date 2020-08-27T 10:42:51
10 Spectrometer Frequency 400.19
11 Spectral Width 8012.8
12 Lowest Frequency -1535.1
13 Nucleus 1H
14 Spectral Size 65536
15 Absolute Reference
1.00 0.88 0.56 0.54 1.12 3.13 5.94 2.00 1.20 0.81 3.92 5.94
f1 (ppm)

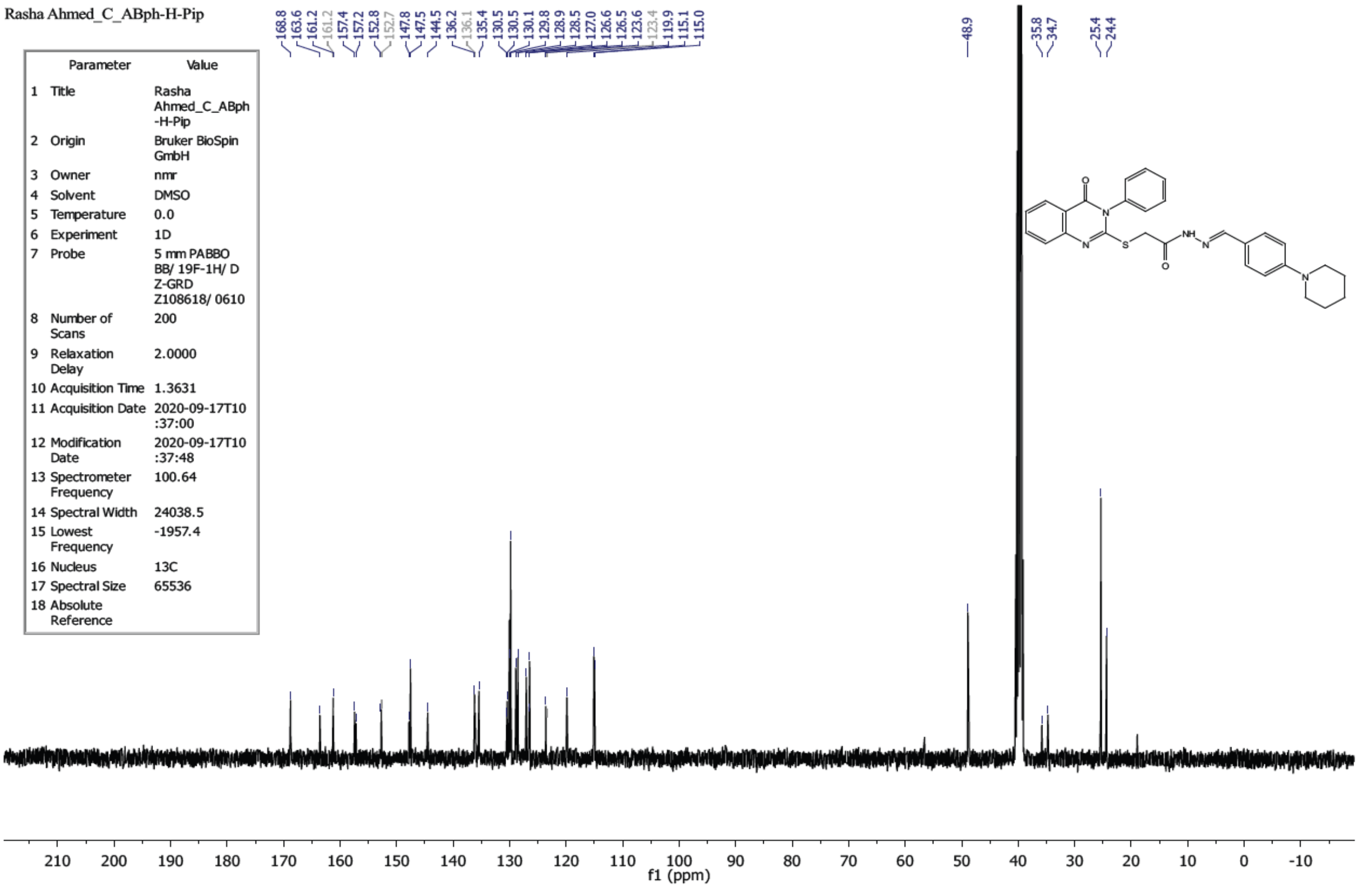
Rasha Ahmed_C_ABph-H-Pip
168.8 163.6 161.2 161.2 157.4 157.2 152.8 152.7 147.8 147.5 144.5 136.2 136.1 135.4 130.5 130.5 130.1 129.8 128.9 128.5 127.0 126.6 126.5 123.6 123.4 119.9 115.1 115.0
48.9 35.8 34.7 25.4 24.4
Parameter Value
1 Title Rasha Ahmed_C_ABph-H-Pip
2 Origin Bruker BioSpin GmbH
3 Owner nmr
4 Solvent DMSO
5 Temperature 0.0
6 Experiment 1D
7 Probe 5 mm PABBO BB/ 19F-1H/ D Z-GRD Z108618/ 0610
8 Number of Scans 200
9 Relaxation Delay 2.0000
10 Acquisition Time 1.3631
11 Acquisition Date 2020-09-17T10 :37:00
12 Modification Date 2020-09-17T10 :37:48
13 Spectrometer Frequency 100.64
14 Spectral Width 24038.5
15 Lowest Frequency -1957.4
16 Nucleus 13C
17 Spectral Size 65536
18 Absolute Reference
f1 (ppm)

Compound 6h

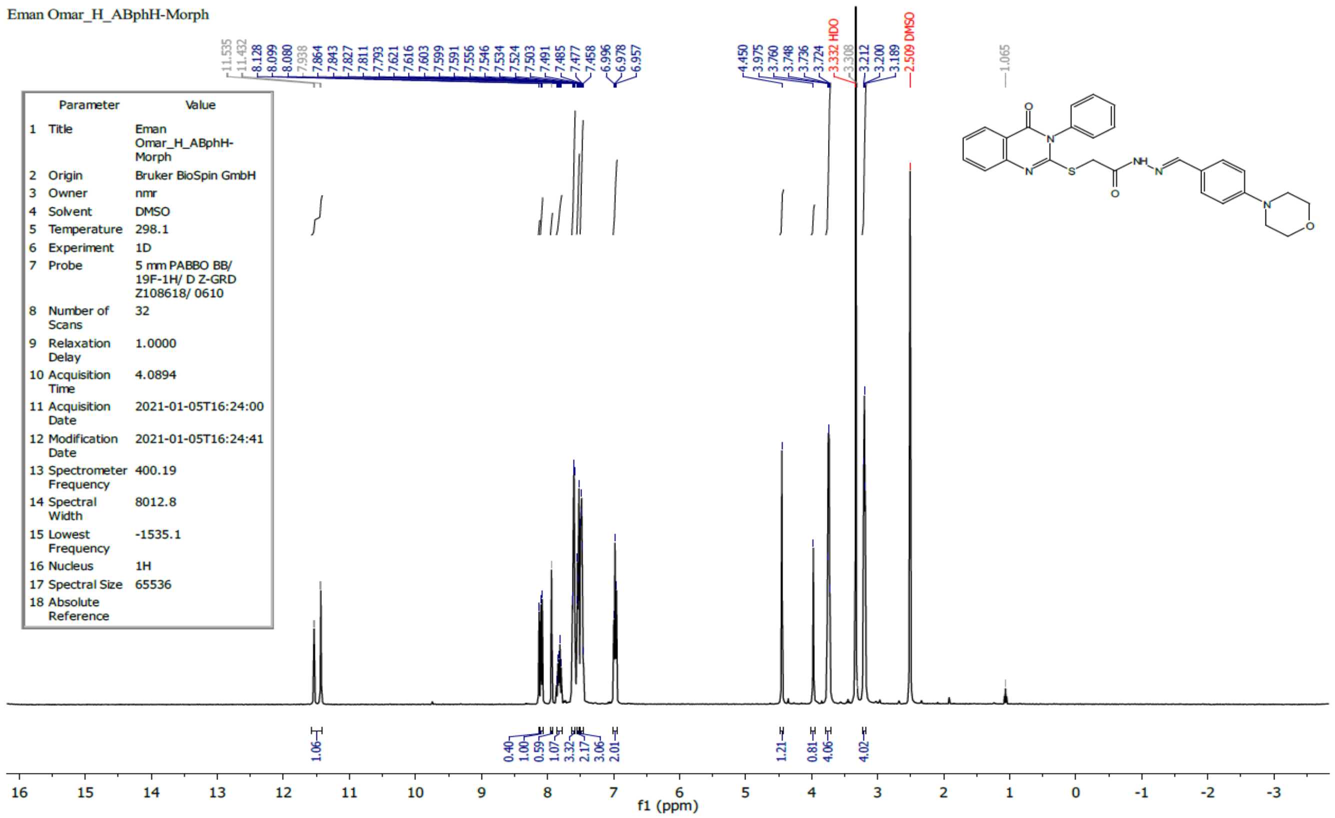
Eman Omar_H_ABphH-Morph
Parameter Value
1 Title Eman Omar_H_ABphH-Morph
2 Origin Bruker BioSpin GmbH
3 Owner nmr
4 Solvent DMSO
5 Temperature 298.1
6 Experiment 1D
7 Probe 5 mm PABBO BB/ 19F-1H/ D Z-GRD Z108618/ 0610
8 Number of Scans 32
9 Relaxation Delay 1.0000
10 Acquisition Time 4.0894
11 Acquisition Date 2021-01-05T16:24:00
12 Modification Date 2021-01-05T16:24:41
13 Spectrometer Frequency 400.19
14 Spectral Width 8012.8
15 Lowest Frequency -1535.1
16 Nucleus 1H
17 Spectral Size 65536
18 Absolute Reference
3.332 HDO
2.509 DMSO
f1 (ppm)

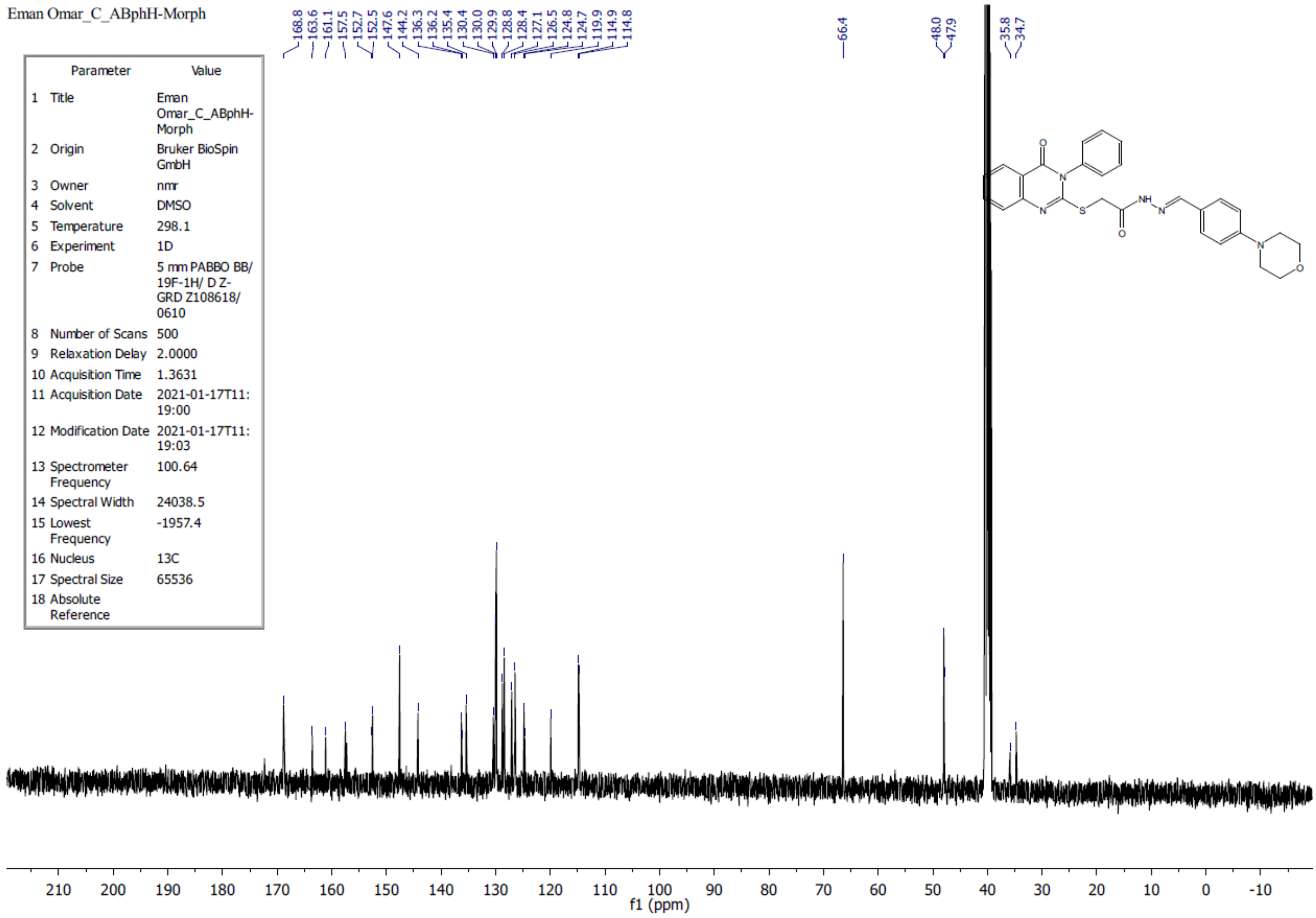
Eman Omar_C_ABphH-Morph
Parameter Value
1 Title Eman Omar_C_ABphH-Morph
2 Origin Bruker BioSpin GmbH
3 Owner nmr
4 Solvent DMSO
5 Temperature 298.1
6 Experiment 1D
7 Probe 5 mm PABBO BB/ 19F-1H/ D Z-GRD Z108618/ 0610
8 Number of Scans 500
9 Relaxation Delay 2.0000
10 Acquisition Time 1.3631
11 Acquisition Date 2021-01-17T11:19:00
12 Modification Date 2021-01-17T11:19:03
13 Spectrometer Frequency 100.64
14 Spectral Width 24038.5
15 Lowest Frequency -1957.4
16 Nucleus 13C
17 Spectral Size 65536
18 Absolute Reference
f1 (ppm)

Compound 6i

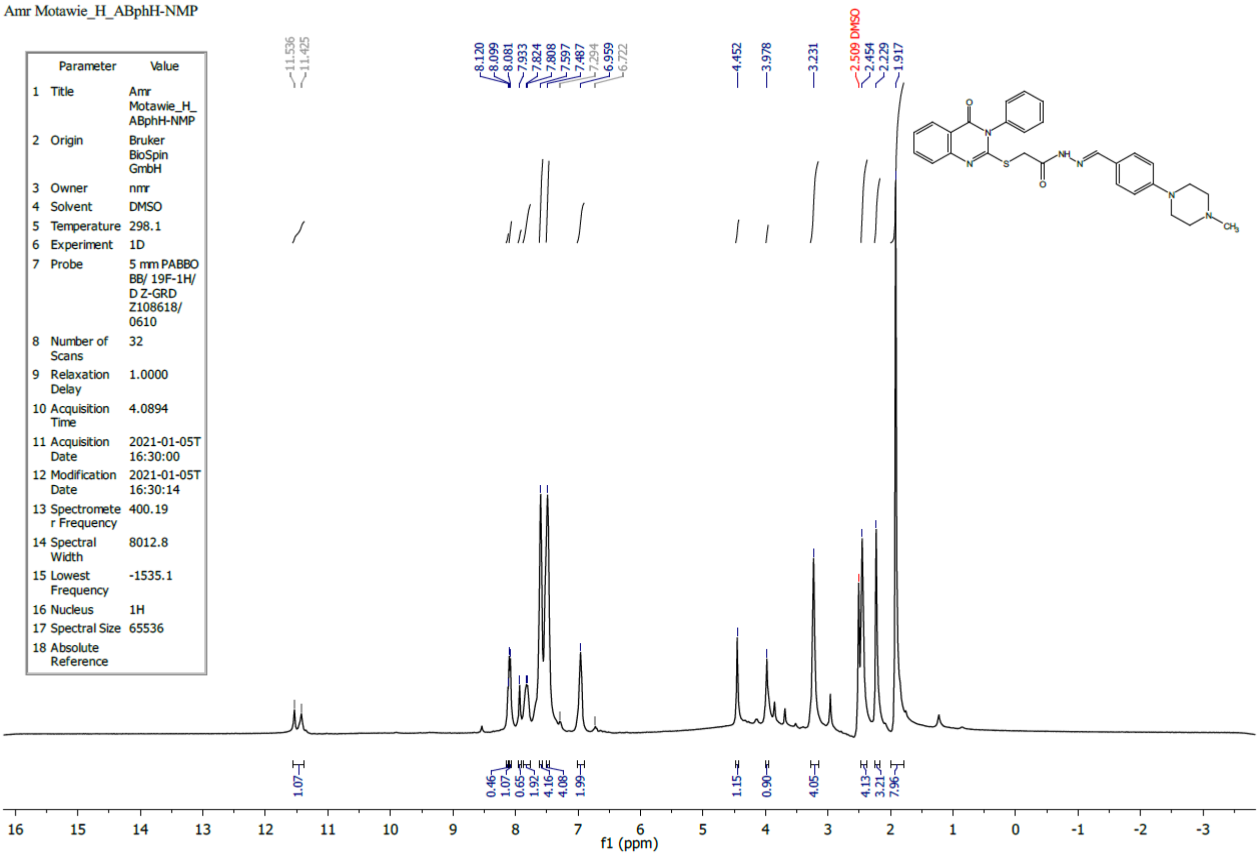

Amr Motawie_H_ABphH-NMP
Parameter Value
1 Title Amr Motawie_H_ABphH-NMP
2 Origin Bruker BioSpin GmbH
3 Owner nmr
4 Solvent DMSO
5 Temperature 298.1
6 Experiment 1D
7 Probe 5 mm PABBO BB/ 19F-1H/ D Z-GRD Z108618/ 0610
8 Number of Scans 32
9 Relaxation Delay 1.0000
10 Acquisition Time 4.0894
11 Acquisition Date 2021-01-05T 16:30:00
12 Modification Date 2021-01-05T 16:30:14
13 Spectrometer Frequency 400.19
14 Spectral Width 8012.8
15 Lowest Frequency -1535.1
16 Nucleus 1H
17 Spectral Size 65536
18 Absolute Reference
11.536
11.425
8.120
8.099
8.081
7.933
7.824
7.808
7.597
7.487
7.294
6.959
6.722
4.452
3.978
3.231
2.509 DMSO
2.454
2.229
1.917
1.07
0.46
1.07
0.65
1.92
4.16
4.08
1.99
1.15
0.90
4.05
4.13
3.21
7.96
f1 (ppm)

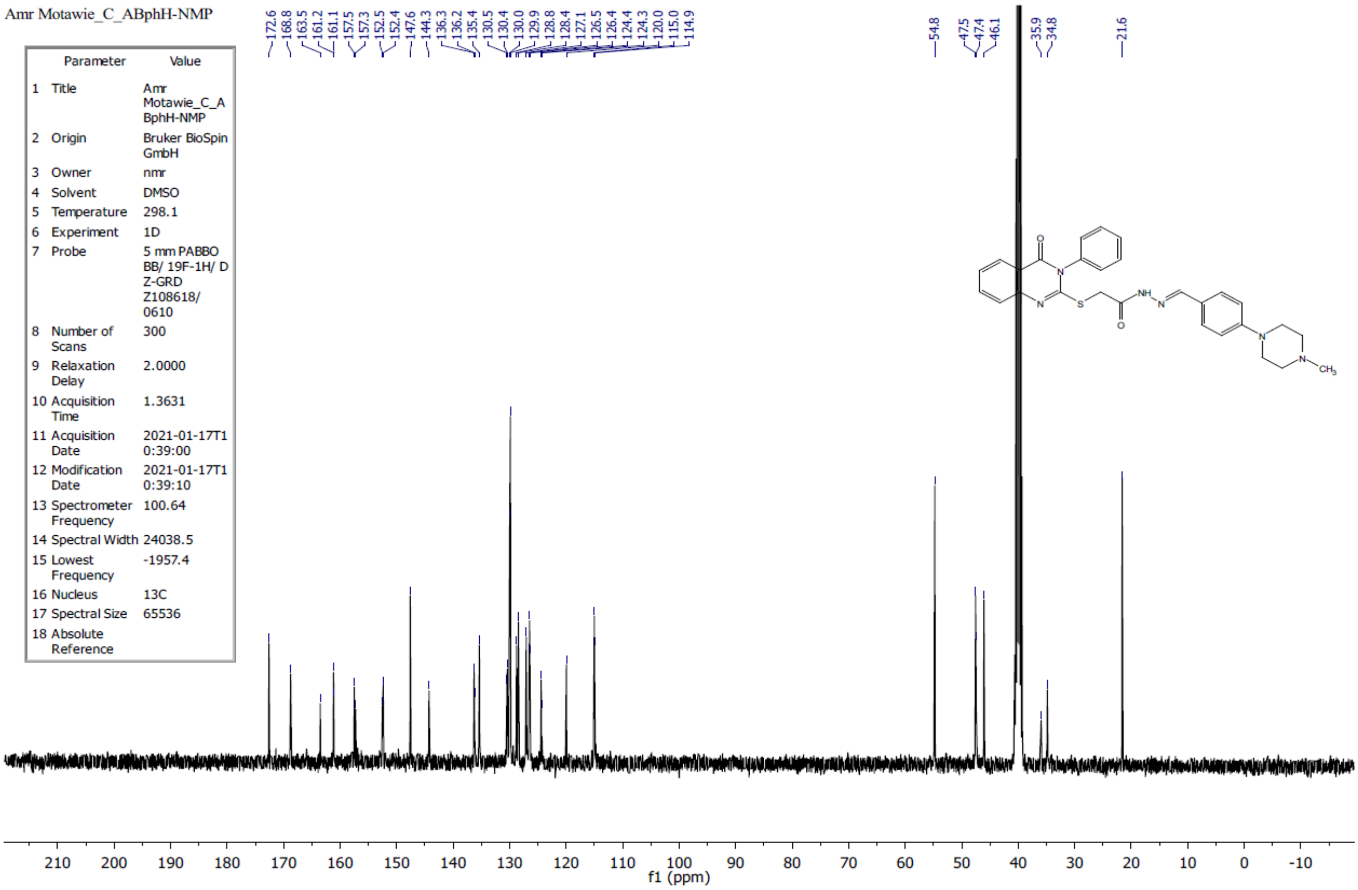

Amr Motawie_C_ABphH-NMP
Parameter Value
1 Title Amr Motawie_C_ABphH-NMP
2 Origin Bruker BioSpin GmbH
3 Owner nmr
4 Solvent DMSO
5 Temperature 298.1
6 Experiment 1D
7 Probe 5 mm PABBO BB/ 19F-1H/ D Z-GRD Z108618/ 0610
8 Number of Scans 300
9 Relaxation Delay 2.0000
10 Acquisition Time 1.3631
11 Acquisition Date 2021-01-17T10:39:00
12 Modification Date 2021-01-17T10:39:10
13 Spectrometer Frequency 100.64
14 Spectral Width 24038.5
15 Lowest Frequency -1957.4
16 Nucleus 13C
17 Spectral Size 65536
18 Absolute Reference
172.6
168.8
163.5
161.2
161.1
157.5
157.3
152.5
152.4
147.6
144.3
136.3
136.2
135.4
130.5
130.4
130.0
129.9
128.8
128.4
127.1
126.5
126.4
124.4
124.3
120.0
115.0
114.9
54.8
47.5
47.4
46.1
35.9
34.8
21.6
f1 (ppm)

Compound 6j

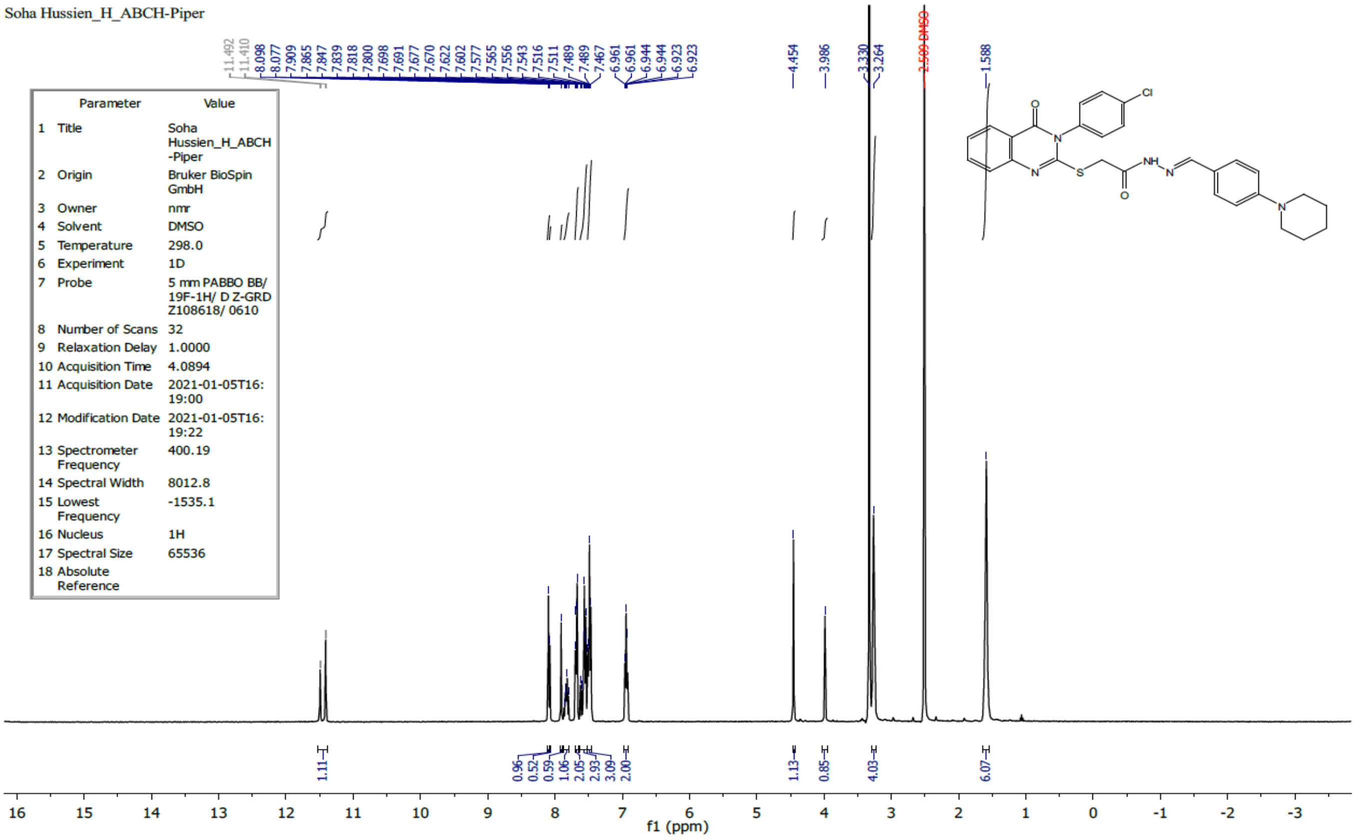

Soha Hussien_H_ABCH-Piper
Parameter Value
1 Title Soha Hussien_H_ABCH -Piper
2 Origin Bruker BioSpin GmbH
3 Owner nmr
4 Solvent DMSO
5 Temperature 298.0
6 Experiment 1D
7 Probe 5 mm PABBO BB/ 19F-1H/ D Z-GRD Z108618/ 0610
8 Number of Scans 32
9 Relaxation Delay 1.0000
10 Acquisition Time 4.0894
11 Acquisition Date 2021-01-05T16:19:00
12 Modification Date 2021-01-05T16:19:22
13 Spectrometer Frequency 400.19
14 Spectral Width 8012.8
15 Lowest Frequency -1535.1
16 Nucleus 1H
17 Spectral Size 65536
18 Absolute Reference
f1 (ppm)

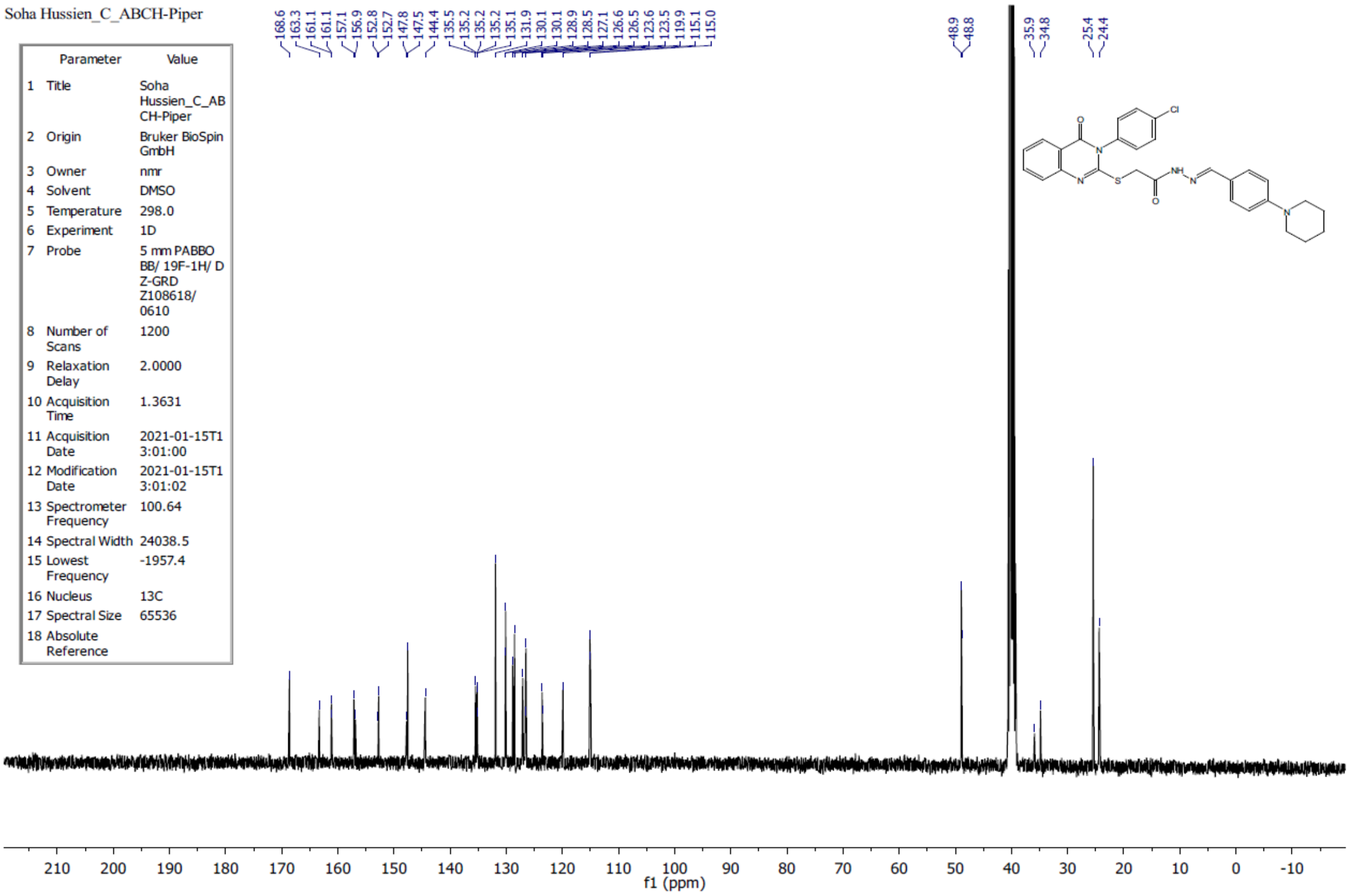

Soha Hussien_C_ABCH-Piper
Parameter Value
1 Title Soha Hussien_C_ABCH-Piper
2 Origin Bruker BioSpin GmbH
3 Owner nmr
4 Solvent DMSO
5 Temperature 298.0
6 Experiment 1D
7 Probe 5 mm PABBO BB/ 19F-1H/ D Z-GRD Z108618/ 0610
8 Number of Scans 1200
9 Relaxation Delay 2.0000
10 Acquisition Time 1.3631
11 Acquisition Date 2021-01-15T13:01:00
12 Modification Date 2021-01-15T13:01:02
13 Spectrometer Frequency 100.64
14 Spectral Width 24038.5
15 Lowest Frequency -1957.4
16 Nucleus 13C
17 Spectral Size 65536
18 Absolute Reference
f1 (ppm)

Compound 6k

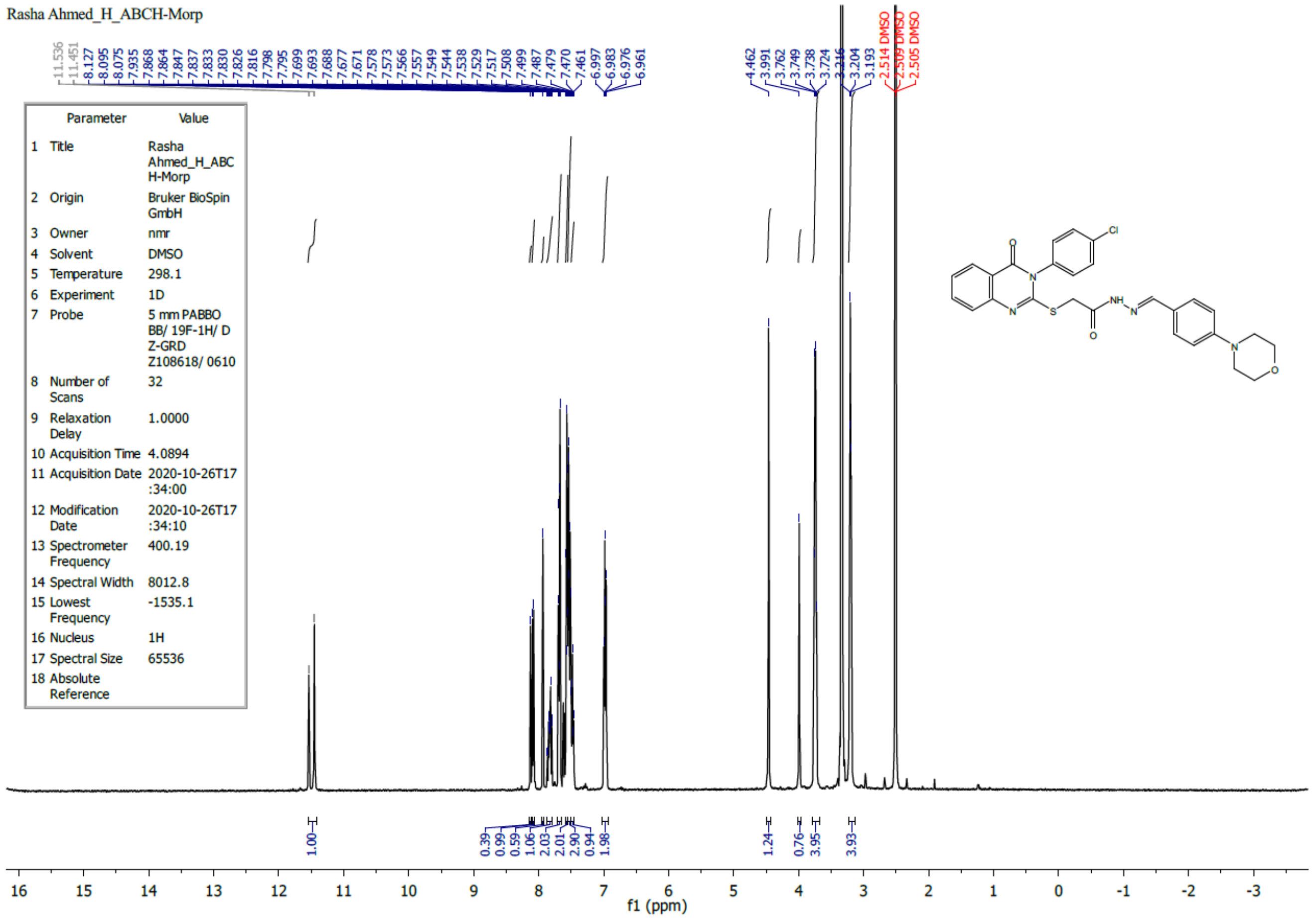

Rasha Ahmed_H_ABCH-Morp
11.536
11.451
8.127
8.095
8.075
7.935
7.868
7.864
7.847
7.837
7.833
7.830
7.826
7.816
7.798
7.795
7.699
7.693
7.688
7.677
7.671
7.578
7.573
7.566
7.557
7.549
7.544
7.538
7.529
7.517
7.508
7.499
7.487
7.479
7.470
7.461
6.997
6.983
6.976
6.961
4.462
3.991
3.762
3.749
3.738
3.724
3.216
3.204
3.193
2.514 DMSO
2.509 DMSO
2.505 DMSO
Parameter Value
1 Title Rasha Ahmed_H_ABCH-Morp
2 Origin Bruker BioSpin GmbH
3 Owner nmr
4 Solvent DMSO
5 Temperature 298.1
6 Experiment 1D
7 Probe 5 mm PABBO BB/ 19F-1H/ D Z-GRD Z108618/ 0610
8 Number of Scans 32
9 Relaxation Delay 1.0000
10 Acquisition Time 4.0894
11 Acquisition Date 2020-10-26T17:34:00
12 Modification Date 2020-10-26T17:34:10
13 Spectrometer Frequency 400.19
14 Spectral Width 8012.8
15 Lowest Frequency -1535.1
16 Nucleus 1H
17 Spectral Size 65536
18 Absolute Reference
1.00
0.39
0.99
0.59
1.06
2.03
2.01
2.90
0.94
1.98
1.24
0.76
3.95
3.93
f1 (ppm)

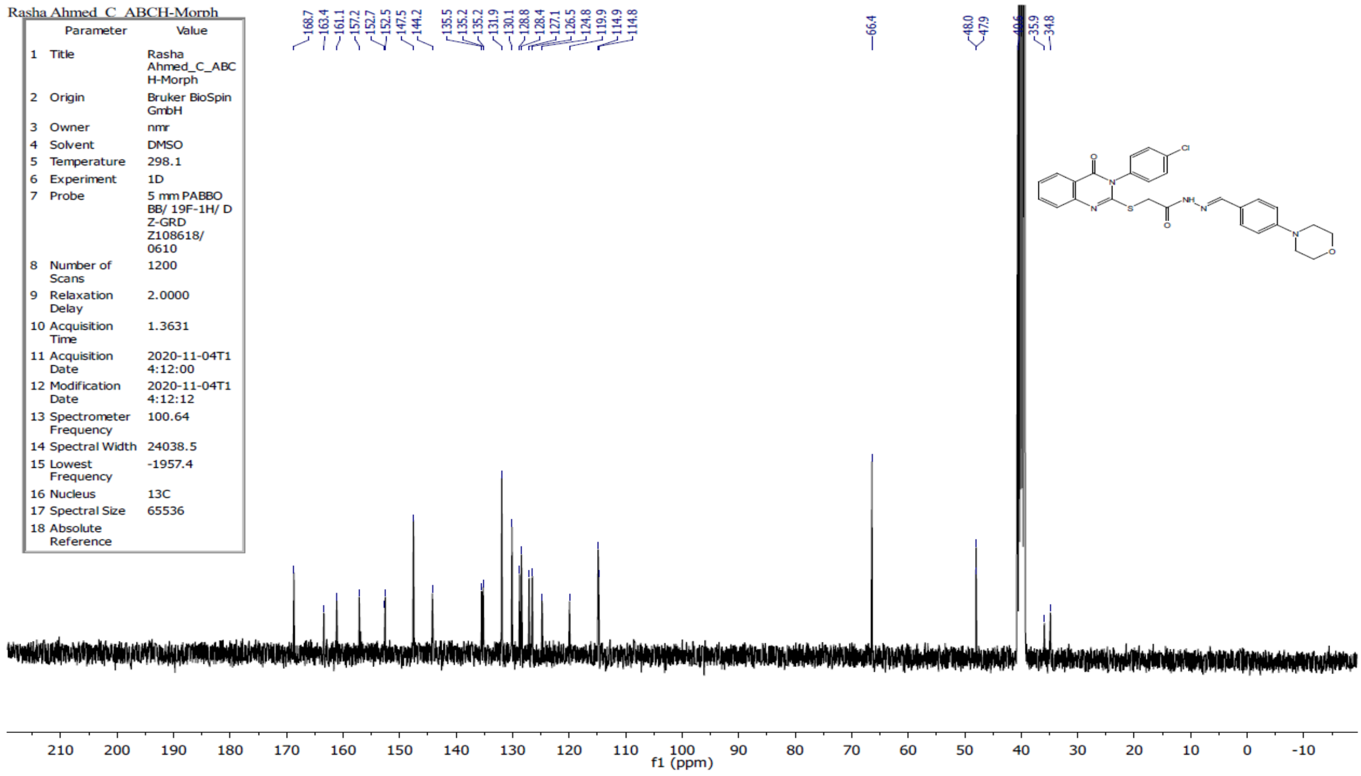

Rasha Ahmed_C_ABCH-Morph
168.7
163.4
161.1
157.2
152.7
152.5
147.5
144.2
135.5
135.2
135.2
131.9
130.1
128.8
128.4
127.1
126.5
124.8
119.9
114.9
114.8
66.4
48.0
47.9
40.6
35.9
34.8
Parameter Value
1 Title Rasha Ahmed_C_ABCH-Morph
2 Origin Bruker BioSpin GmbH
3 Owner nmr
4 Solvent DMSO
5 Temperature 298.1
6 Experiment 1D
7 Probe 5 mm PABBO BB/ 19F-1H/ D Z-GRD Z108618/ 0610
8 Number of Scans 1200
9 Relaxation Delay 2.0000
10 Acquisition Time 1.3631
11 Acquisition Date 2020-11-04T14:12:00
12 Modification Date 2020-11-04T14:12:12
13 Spectrometer Frequency 100.64
14 Spectral Width 24038.5
15 Lowest Frequency -1957.4
16 Nucleus 13C
17 Spectral Size 65536
18 Absolute Reference
f1 (ppm)

Compound 6l

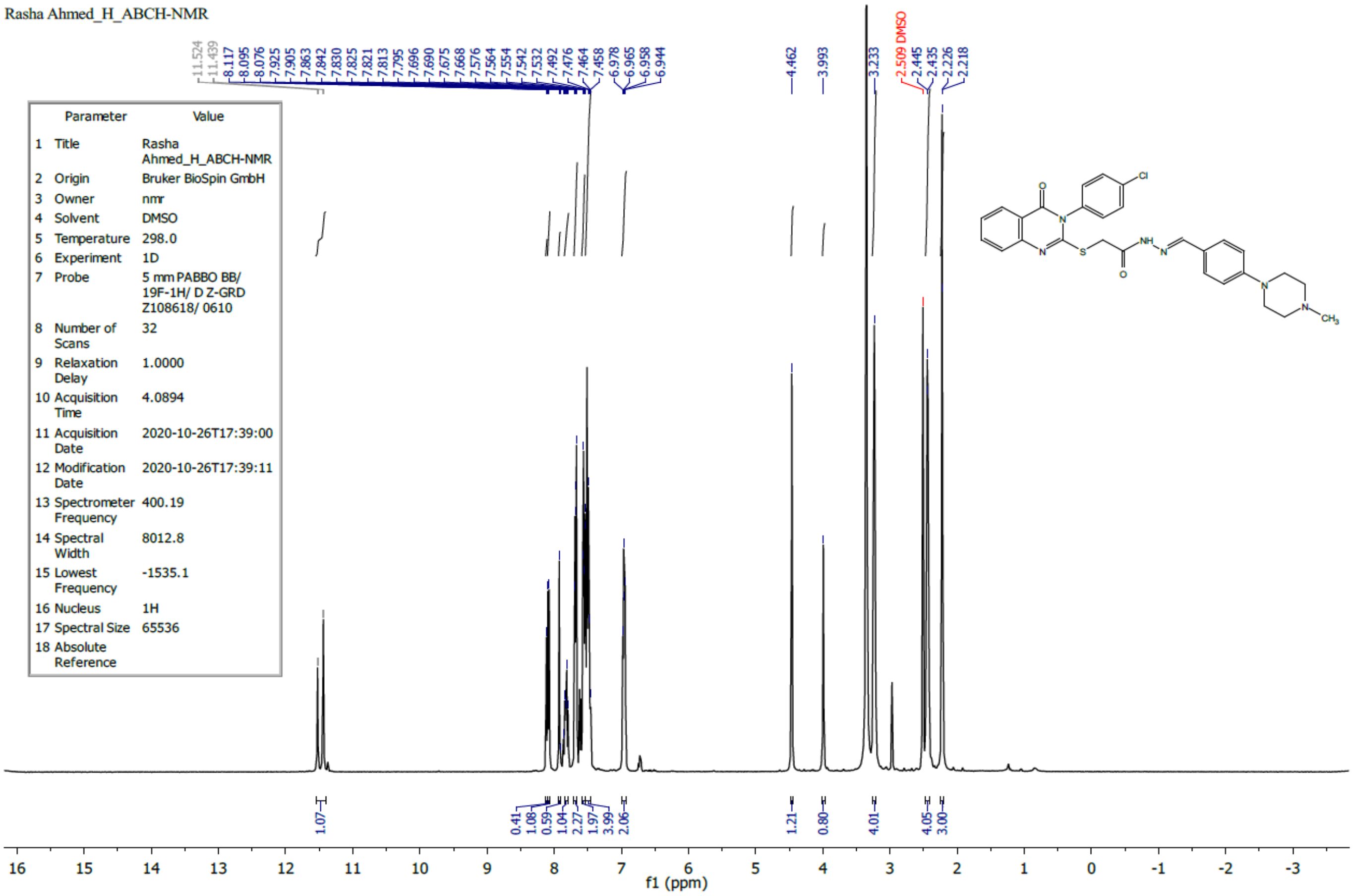

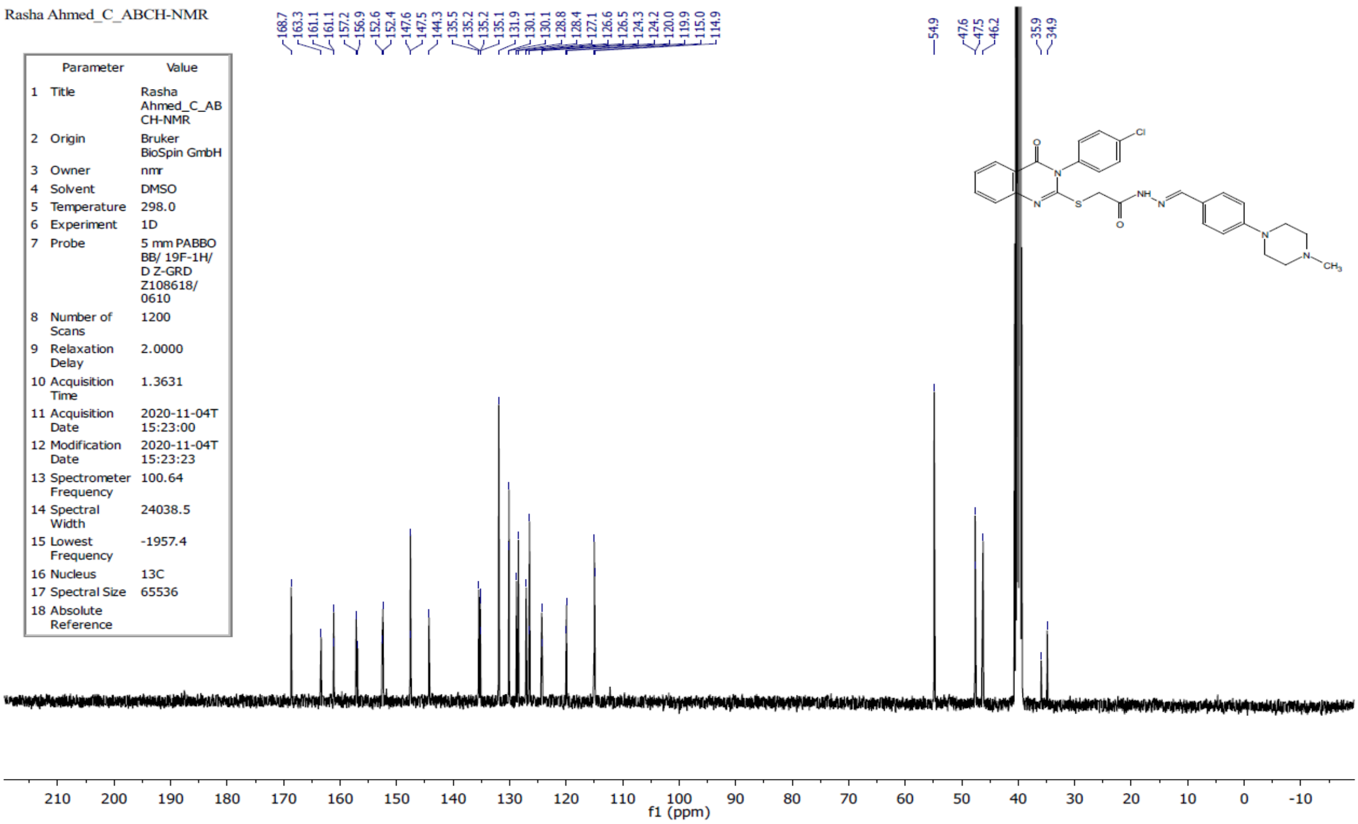

Supplement: Supplemental Material [file IENZ_A_2118735_SM7599.pdf]
